# Structure and inhibition of diaminopimelic acid epimerase by slow‐binding *α*‐methyl amino acids

**DOI:** 10.1002/pro.70139

**Published:** 2025-04-29

**Authors:** Tess Lamer, Pu Chen, Karizza Catenza, Ilia Perov, Bethan L., Yu‐Ting Hsiao, Tayla J. Van Oers, M. Joanne Lemieux, John C. Vederas

**Affiliations:** ^1^ Department of Chemistry University of Alberta Edmonton Alberta Canada; ^2^ Department of Biochemistry University of Alberta Edmonton Alberta Canada; ^3^ Li Ka Shing Institute of Virology, University of Alberta Edmonton Alberta Canada

**Keywords:** antibiotic, cofactor‐independent, crystal structure, DAP epimerase, DapF, diaminopimelic acid, enzyme mechanism, inhibitor, lysine biosynthesis, protein biochemistry

## Abstract

Cofactor‐independent racemases and epimerases produce D‐amino acids from their L‐isomers for a variety of biological processes. These enzymes operate via an unusual mechanism that relies on an active site cysteine thiolate (p*K*_a_ ~ 8.5) to deprotonate an amino acid *α*‐carbon (p*K*_a_ ~ 29) and are of interest not only because of their biocatalytic potential for D‐amino acid production, but also because many play key roles in biology and are antibiotic targets. However, obtaining crystal structures of these enzymes, especially in their closed, substrate‐ or inhibitor‐bound conformations, is difficult. In this work, we characterized diaminopimelic acid (DAP) epimerase from the cyanobacterium *Anabaena*. DAP epimerase has long been of interest as an antibiotic target as it converts L,L‐DAP to D,L‐DAP for lysine and peptidoglycan biosynthesis. We solved three crystal structures of this enzyme in its closed, inhibitor‐bound conformation, up to a resolution of 1.5 Å. Two structures show the enzyme covalently bound through its catalytic cysteine residues to previously reported aziridine‐based inhibitors. One structure unexpectedly shows the enzyme bound to a different compound, D,L‐*α*‐methylDAP, presumably produced as a synthetic byproduct. Stereoselective synthesis of L,L‐ and D,L‐*α*‐methylDAP followed by inhibition assays shows that these compounds are slow‐binding inhibitors of DAP epimerase. *α*‐MethylDAP inhibitors provide a more accessible alternative to aziridine‐based inhibitors to obtain crystal structures of DAP epimerase in its closed conformation. Comparisons of bacterial, cyanobacterial, and plant DAP epimerases provided here offer new insights into functional and structural differences between these enzymes.

## 1 INTRODUCTION

**FIGURE 1:**
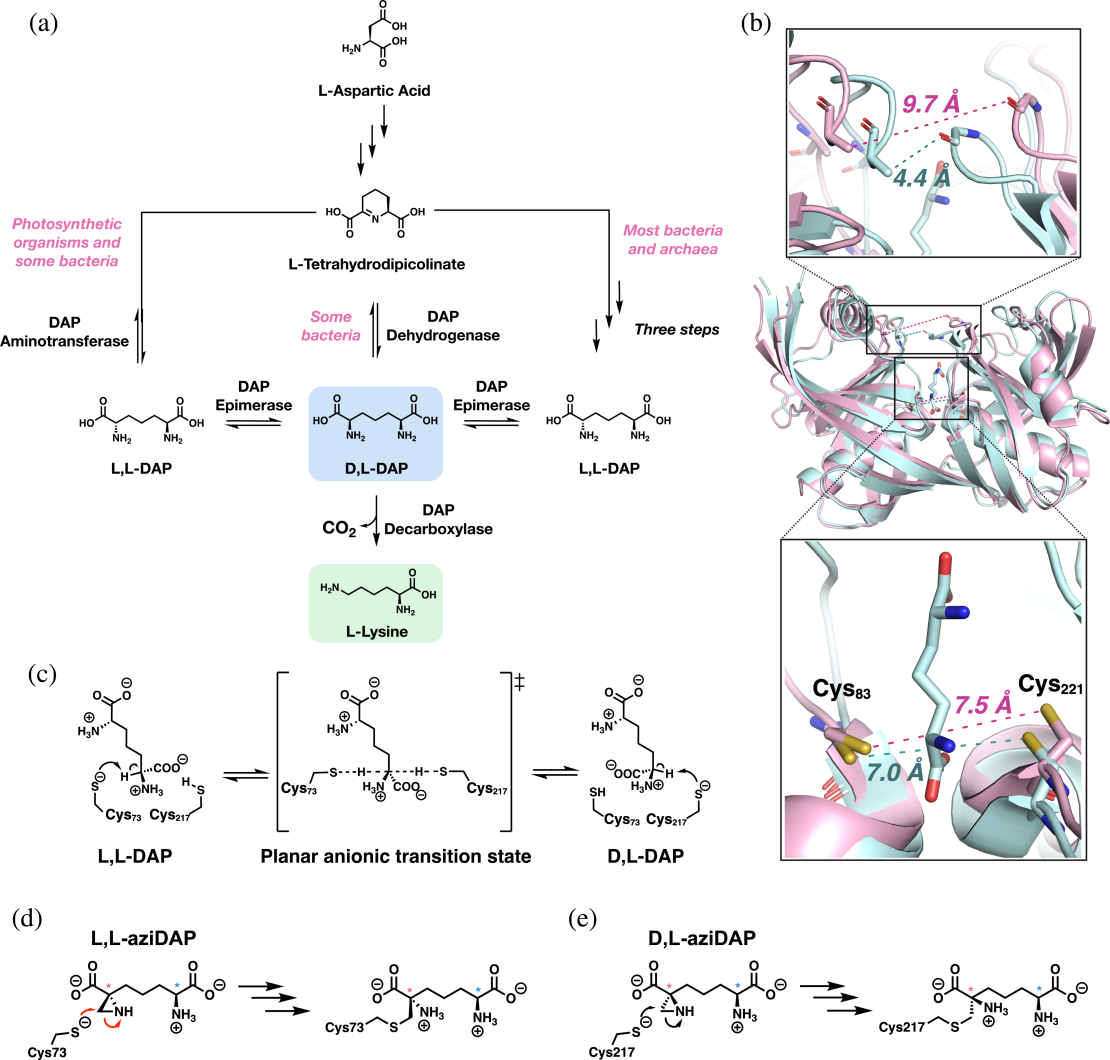
Role and mechanism of DAP epimerase. (a) Lysine biosynthetic pathway in most prokaryotes and photosynthetic organisms. (b) Crystal structures of DapF from *Corynebacterium glutamicum* (PDB ID 5M47) in its open, unbound (pink) and closed, D,L‐DAP‐bound (blue) conformations. Catalytic cysteine residues are shown in the bottom panel. (c) Chemical mechanism of *Haemophilus influenzae* DapF. (d) Abbreviated mechanism of covalent, irreversible inhibition of *H. influenzae* DapF by L,L‐aziDAP and (e) D,L‐aziDAP.

2,6‐Diaminopimelic acid (DAP) epimerase catalyzes epimerization of L,L‐DAP to D,L‐DAP—the penultimate step of lysine biosynthesis in most bacteria and photosynthetic organisms (Figure 1a) (Muduli et al., 2023). This enzyme is also important for bacterial peptidoglycan biosynthesis, as lysine and D,L‐DAP are used as the pentapeptide crosslinkers in most Gram‐positive and Gram‐negative species, respectively. DAP epimerase (DapF) is a critical enzyme in bacterial biology, absent in mammals, and has a unique structure and mechanism, making it a long‐standing target for antibiotic drug development (Cox et al., 2000; Hutton et al., 2007). DapF belongs to the class of amino acid racemase and epimerase enzymes that are cofactor‐independent (pyridoxal 5′‐phosphate (PLP)‐independent) (Fischer et al., 2019; Lloyd et al., 2021). Within the cofactor‐independent class of racemases and epimerases, there are two structural subclasses: the glutamate racemase‐like and the DapF‐like. Other cofactor‐independent enzymes with the DapF‐like fold include histidine racemase, proline racemase, 4‐hydroxyproline epimerase, *O‐*ureidoserine racemase, isoleucine epimerase, and 2,4‐diaminobutyric acid racemase, which are involved in diverse biological processes such as metallophore biosynthesis, antibiotic biosynthesis, pathogenicity of the human parasite *Trypanosoma cruzi*, and amino acid catabolism (Bearne, 2020; Fischer et al., 2019; Lamer et al., 2024; Lloyd et al., 2021; Luo et al., 2019; Strauch et al., 2015; Yamanaka et al., 2021). Like DapF, many of these enzymes are excellent drug targets because of their biological importance and lack of homologous enzymes in mammals.

The chemical mechanism of cofactor‐independent racemases and epimerases with the DapF‐like fold is unusual due to its reliance on seemingly mismatched p*K*_a_ values. Using DapF as an example, upon substrate binding, the enzyme undergoes a conformational change from its open, substrate‐unbound form to its closed, substrate‐bound form (Figure 1b) (Pillai et al., 2006, 2009; Sagong & Kim, 2017). This movement expels water from the active site pocket and seals the enzyme around its substrate, satisfying H‐bonding requirements of both molecules. The key catalytic residues are two active site cysteines that form an unusual thiol‐thiolate pair at physiological pH, situated on either side of the substrate *α*‐C. In a unique, reversible mechanism, the thiolate (p*K*_a_ ~ 8.5) functions as a general base to deprotonate the substrate's *α*‐C (p*K*_a_ ~ 29) (Richard et al., 2014), while the thiol acts as a general acid to supply a proton to the planar anionic transition state from the opposite side, resulting in epimerization of DAP (Figure 1c).

In the case of DapF, much effort has been spent synthesizing inhibitors as potential antibiotic drug leads (Table S1) (Abbott et al., 1994; Baumann et al., 1988; Caplan et al., 2000; Cox et al., 2002; Diaper et al., 2005; Gelb et al., 1990; Gerhart et al., 1990; Higgins et al., 1989; Lam et al., 1988; Song et al., 1994). Despite the wide variety of compounds explored, the best inhibitors reach *K*_i_ or IC_50_ values only in the low micromolar range. Obtaining crystal structures of these highly mobile enzymes to better understand their mechanism and design improved inhibitors is extremely challenging. Crystal structures of DapF and other cofactor‐independent racemases and epimerases in their open, substrate‐unbound conformation are often obtained via mutation of one of the catalytic cysteine residues to a serine residue, which hinders catalysis and aids crystallization of the enzyme in its open conformation (Table S2) (Buschiazzo et al., 2006; Lamer et al., 2024; Luo et al., 2019; Pillai et al., 2007). Furthermore, AlphaFold can now predict these enzymes in their open form with a high degree of accuracy. However, few insights into binding and chemical mechanism can be provided without a crystal structure of the enzyme in its closed, active (substrate/inhibitor‐bound) conformation. There is currently not a general strategy available to obtain structures of enzymes with the DapF‐like fold in their closed conformation, and a limited number of these structures have been solved (Pillai et al., 2006, 2009; Sagong & Kim, 2017). Elucidation of the closed, active conformation of DAP epimerase from *Haemophilus influenzae* was first achieved via chemical synthesis of pure stereoisomers of aziridine analogues of DAP: (2*S*,4*S*)‐ and (2*S*,4*R*)‐2‐(4‐amino‐4‐carboxybutyl)aziridine‐2‐carboxylic acid (L,L‐ and D,L‐aziDAP, respectively) (Diaper et al., 2005; Pillai et al., 2006). These covalent, irreversible inhibitors “lock” the enzyme in its closed conformation, thereby aiding the crystallization process and providing insight into binding and mechanism (Figure 1d–e). Only two other DapF enzymes (from the plant *Arabidopsis thaliana* and bacteria *Corynebacterium glutamicum*) have been crystallized in the closed conformation, bound by aziDAP inhibitors or the substrate D,L‐DAP, respectively (Table S2) (Pillai et al., 2009; Sagong & Kim, 2017).

Unfortunately, the synthesis of aziDAP inhibitors is challenging and not generalizable for studies of other enzymes with the DapF‐like fold. Furthermore, the lack of crystal structures of cofactor‐independent racemases and epimerases with the DapF‐like fold in their closed conformations has made it difficult to predict the role of new members of this enzyme class, which are typically annotated as putative DapF enzymes. The result has been a very slow increase in the characterization of new enzymes of this class, which are widespread in nature from all domains of life, and almost certainly with new types to be discovered. Additionally, the development of these enzymes as biocatalysts to produce valuable D‐amino acids has not yet been achieved, likely due to the challenge of reaction reversibility (Bearne, 2020, 2021). Further insights provided by structural and functional studies are required for enzyme engineering efforts to be justified.

We were initially interested in obtaining a crystal structure of a photosynthetic cyanobacterial DAP epimerase (from *Anabaena* sp. YBS01) in its closed conformation to compare previously solved structures of plant and bacterial DapF enzymes. Functionally, we found that plant and cyanobacterial DAP epimerases tolerated lanthionine as a substrate and had a similar kinetic profile, while the bacterial enzyme did not tolerate lanthionine and had slightly different kinetic parameters. We synthesized D,L‐ and L,L‐aziDAP inhibitors and crystallized *Anabaena* DapF with each, providing three crystal structures with resolutions up to 1.5 Å. Unexpectedly, the crystals obtained of D,L‐aziDAP bound to *Anabaena* DapF also contained monomers with the enzyme non‐covalently bound to a different compound, (2*R*,6*S*)‐2,6‐diamino‐2‐methylheptanedioic acid (D,L‐*α*‐methylDAP). This compound may have been produced as a synthetic byproduct. We proceeded to develop stereoselective syntheses of a series of *α*‐methylDAP analogues and found that these compounds are slow‐binding inhibitors of DapF. The straightforward synthesis of *α*‐methylDAP inhibitors compared to that of aziDAP inhibitors provides a new strategy to obtain crystal structures of DAP epimerase in its closed conformation, which are difficult to obtain.

## 2 RESULTS

### 2.1 Kinetic, sequence analysis, and functional characterization of DapF from *Anabaena* sp. YBS01

DAP epimerase from the cyanobacterium *Anabaena* sp. YBS01 (GenBank: CP034058.1), an organism isolated from soil in Meghalaya, India, was cloned, overexpressed, and purified from *Escherichia coli* BL21(DE3) with a C‐terminal hexahistidine tag. A coupled enzyme assay with DAP dehydrogenase from *Symbiobacterium thermophilum* IAM 14863 in the presence of L,L‐DAP and NADP^+^ was used to determine kinetic parameters of the DAP epimerase (Table 1). In this kinetic assay, L,L‐DAP is converted to D,L‐DAP by DapF, which is then consumed with NADP^+^ by DAP dehydrogenase to produce L‐tetrahydrodipicolinic acid and NADPH (Lam et al., 1988). NADPH production can be monitored by the rate of increasing absorption at 340 nm. The dehydrogenase is used in excess to the epimerase to ensure epimerization is the rate‐determining step. The N‐terminally hexahistidine‐tagged DAP dehydrogenase was cloned and overexpressed in *E. coli* according to a literature procedure (Gao et al., 2012). Kinetic parameters of *Anabaena* DapF were found to be comparable to other DAP epimerases, though they were most similar to that of *A. thaliana* (Table 1).

**TABLE 1:** Kinetic parameters of DapF enzymes with L,L‐DAP.

Organism	*K*_M_ (μM)	*k*_cat_ (s^−1^)	*k*_cat_/*K*_M_ (M^−1^ s^−1^)
*Anabaena* sp. YBS01	63	9.5	1.5 × 10^5^
*H. influenzae* (Koo & Blanchard, 1999)	700	128	1.8 × 10^5^
*E. coli* (Hor et al., 2013)	124	104	8.4 × 10^5^
*A. thaliana*	68	3.0	4.4 × 10^5^
*C. glutamicum* (Sagong & Kim, 2017)	1860	58	3.1 × 10^4^

Of other DapF enzymes that have crystal structures obtained in the closed, substrate−/inhibitor‐bound conformation (*H. influenzae*, *A. thaliana*, and *C. glutamicum*), *Anabaena* DapF has the highest sequence identity to that of *A. thaliana* (65%), and lower identity to *H. influenzae* (40%) and *C. glutamicum* (36%). Low sequence identity amongst cofactor‐independent epimerases and racemases with the same substrate is not uncommon; however, key active site residues previously suggested to form H‐bonds with DAP are generally conserved in *Anabaena* DapF (Figure S1).

A ^1^H‐NMR‐based assay was conducted in D_2_O to identify substrates of *Anabaena* DapF, which relies on the disappearance of a substrate's *α*‐H signal as it is replaced with deuterium by the epimerase enzyme. We were surprised to observe that in addition to D,L‐ and L,L‐DAP, D,L‐ and L,L‐lanthionine were also tolerated as substrates by this enzyme. Despite the seemingly minor change in the structure of the substrate between DAP and lanthionine (replacement of C*^γ^* with a sulfur atom), our group has previously observed that an oxa‐analogue of DAP (replacement of C*^γ^* with an oxygen atom) is a poor substrate of *H. influenzae* DapF. Turnover of this oxa‐analogue was only 26% of the initial velocity with L,L‐DAP (H. Liu et al., 2007). We were also surprised to find that while lanthionine has been previously tested as an inhibitor of DAP epimerase (Lam et al., 1988), to the best of our knowledge, it has not so far been tested and reported as a substrate of a purified DAP epimerase.

**FIGURE 2:**
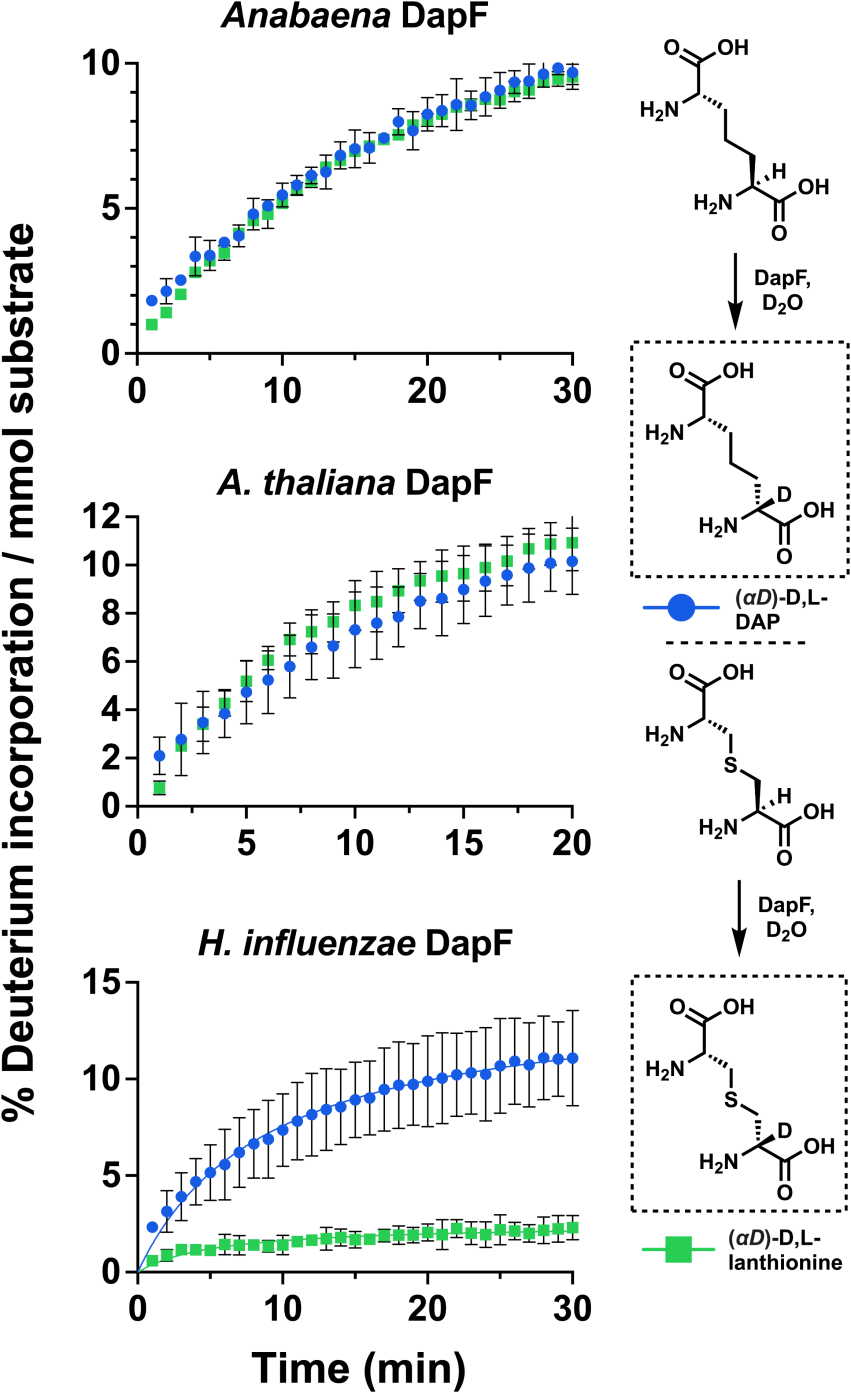
Tolerance of lanthionine as a substrate varies amongst DAP epimerases. The rate of decrease in the *α*‐H signal integration of L,L‐DAP or L,L‐lanthionine (rate of deuterium incorporation) over time was monitored using ^1^H‐NMR after DapF was added to a 5 mM solution of either substrate in a deuterated buffer. Experiments were performed in duplicate.

To directly compare epimerization rates of L,L‐lanthionine and L,L‐DAP by *Anabaena* DapF, 5 mM solutions of either substrate in D_2_O buffer were prepared, followed by the addition of DapF and monitoring of the rate of depletion of the *α*‐H signal over time with ^1^H‐NMR (Figure 2). For *Anabaena* DapF, the rate of deuterium incorporation was equal for lanthionine and DAP. We repeated this experiment with DapF from *A. thaliana* and observed the same equal rate of substrate turnover with this plant enzyme. However, for *H. influenzae* DapF, the initial velocity with L,L‐lanthionine was only ~20% that of L,L‐DAP, confirming the result previously observed with 4‐oxaDAP by our group. To confirm that lanthionine epimerization was indeed occurring (and not just deprotonation and replacement of the *α*‐H with a deuterium atom) circular dichroism experiments were conducted with each enzyme using L,L‐lanthionine as a substrate (Figure S2). Both *A. thaliana* and *Anabaena* DapF epimerized lanthionine, while *H. influenzae* DapF did not under the time frame observed. However, the bacterial enzyme was able to epimerize a 5 mM solution of L,L‐DAP, indicating that the enzyme was active and providing further evidence that the ability to epimerize lanthionine is not universal for DAP epimerases.

### 2.2 Crystal structures of *Anabaena* DapF covalently bound to D,L‐ and L,L‐aziDAP

**FIGURE 3:**
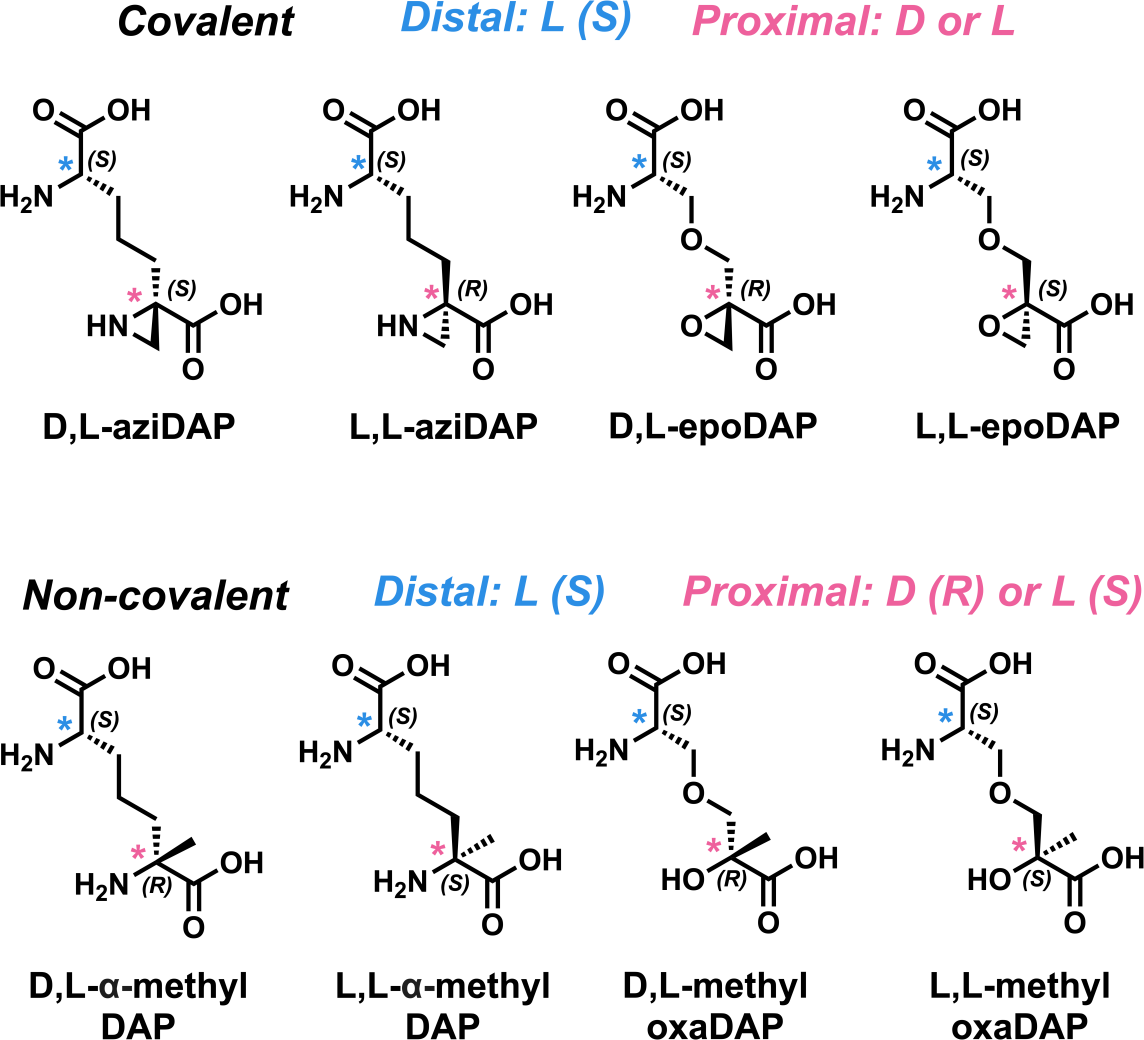
DAP analogues synthesized as pure stereoisomers for DapF inhibition. The top four structures are covalent inhibitors, while the bottom four are non‐covalent, with stereochemistry of proximal and distal *α*‐carbons indicated. The proximal *α*‐C (pink *) is the catalytic center when bound to DapF.

To obtain a crystal structure of *Anabaena* DapF in its closed conformation, several potential covalent inhibitors were synthesized (Figure 3). The use of pure stereoisomers of inhibitors for X‐ray crystallography is required to obtain high‐resolution structures of the DapF active site.

Previously described irreversible, covalent DAP epimerase inhibitors D,L‐ and L,L‐aziDAP were synthesized stereoselectively according to a literature procedure (Figure S3) (Diaper et al., 2005). We also developed a route to produce pure stereoisomers of an epoxide analogue of DAP: (*S*)‐2‐(((*S*)‐2‐amino‐2‐carboxyethoxy)methyl)oxirane‐2‐carboxylic acid and (*R*)‐2‐(((*S*)‐2‐amino‐2‐carboxyethoxy)methyl)oxirane‐2‐carboxylic acid (L,L‐ and D,L‐epoDAP, respectively; Figure S4). The epoxide inhibitors were based on previously reported epoxide analogues of *O*‐ureidoserine (Ahn et al., 2018). Unfortunately, there was no measurable inhibition of DapF after overnight incubation of the enzyme with these epoxides (Figure S5). However, aziDAP inhibition of *Anabaena* DapF (and *A. thaliana* DapF, used as a control) was observed after overnight incubation (Figure S5). A larger scale incubation with D,L‐aziDAP was successful in producing two sets of crystals suitable for diffraction to a resolution of 1.5 Å (PDB ID 9MRO) and 1.7 Å (PDB ID 9MRV), while incubation with L,L‐aziDAP produced a structure with a resolution of up to 1.5 Å (PDB ID 9MRP). Data collection and refinement statistics are shown in Table S3.

**FIGURE 4:**
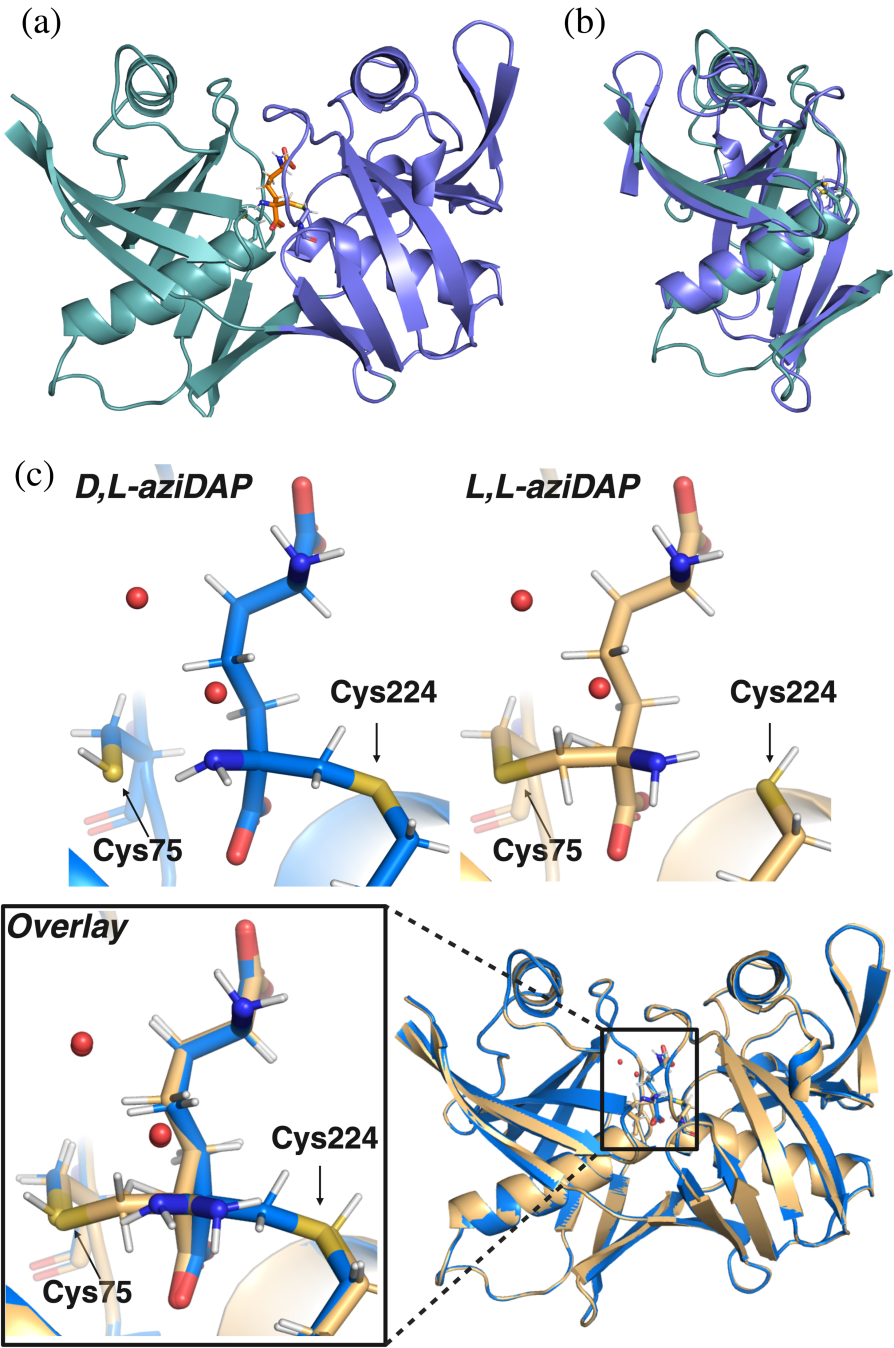
Crystal structures of *Anabaena* DapF in its closed, aziDAP‐bound conformation. (a) Overall pseudosymmetry of *Anabaena* DapF N‐terminal (teal) and C‐terminal domains (purple). D,L‐aziDAP is shown in orange bound in the active site. (b) Structural alignment of N‐terminal and C‐terminal domains. (c) Structures of D,L‐ and L,L‐aziDAP covalently bound to *Anabaena* DapF through Cys224 and Cys75, respectively. The bottom panels show an alignment of the two crystal structures.

The overall structure of *Anabaena* DapF is typical of DAP epimerases and consists of an *α* + *β* structure with pseudosymmetric N‐terminal and C‐terminal domains (Figure 4a). The N‐terminal domain (residues 1–123 and 269–280) is composed of two antiparallel *β*‐sheets (*β*1, *β*2, *β*3, *β*4, *β*17 and *β*5, *β*6, *β*7) and *α*‐helices 1 and 2 (Figure S6). The C‐terminal domain (residues 124–268) is composed of a second set of antiparallel *β*‐sheets (*β*8, *β*9, *β*10 and *β*11, *β*12, *β*14, *β*15, *β*16) and *α*‐helices 3 and 4. The two domains have a root mean square deviation (RMSD) of 3.2 Å over 112 C^*α*^ pairs (Figure 4b). Irreversible, covalent aziDAP inhibitors are bound in the active site through a thioether bond between the inhibitor methylene carbon and the thiol of Cys224 (D,L‐aziDAP) or Cys75 (L,L‐aziDAP), formed after nucleophilic ring‐opening of the aziridine (Figure 4c). The alkylated enzyme is an excellent mimic for the closed, substrate‐bound conformation of DapF, offering insight into the unusual deprotonation of DAP's C^*α*^ by a weak thiolate base. The two aziDAP‐bound structures have an RMSD of 0.118 Å over 280 C^*α*^ pairs.

Structural features that have been previously identified to account for the reduced p*K*_a_ values (experimentally determined to be 6.5 and 8.5 in *H. influenzae* DapF) of the thiols of catalytic cysteine residues appear to be conserved in *Anabaena* DapF, suggesting conservation of the unusual thiol‐thiolate pair at physiological pH in this cyanobacterial enzyme. These features include the positioning of these cysteines at the N‐termini of *α*‐helices 2 and 4, which may stabilize the thiolate negative charge by the effective positive charge of the helix vector dipole (Kortemme & Creighton, 1995). Additionally, catalytic sulfur atoms are within H‐bonding distance to the backbone amides of Gly76 (Cys75) and Gly227 (Cys224), which may further stabilize the thiolate negative charge. Finally, there are no basic residues in close proximity to Cys75 and Cys224 S*γ* atoms (<4.0 Å), providing further evidence for a thiol‐thiolate pair mechanism of action in *Anabaena* DapF.

The *Anabaena* DapF active site satisfies H‐bonding requirements of the inhibitor molecule with highly conserved active site residues (Figures S1 and S7). At the proximal end of the inhibitor molecule (C^*α*^ positioned between the catalytic cysteine residues), the *α*‐carboxylate appears to form five H‐bonds with H‐bond donors of the enzyme (backbone amide NH of Gly76, Asn77, Gly225, and Thr226, and side chain‐OH^*γ*1^ of Thr226), suggesting a negatively charged carboxylate. The proximal end of the inhibitor molecule is positioned at the N‐termini of helices 2 and 4, allowing for dispersion of the presumable negative charge on the carboxylate of the inhibitor by the vectors of the helices' dipoles (Richard & Amyes, 2004). Dispersion of charge and satisfying the carboxylate's H‐bonding requirements may help to decrease the p*K*_a_ of the *α*‐H and aid catalysis (Gerlt et al., 1991). Additionally, the proximal *α*‐amine is within H‐bonding distance to a water molecule, Asn13 side chain O^*δ*1^, and Cys75 S^*γ*^ (Figure S7). This suggests a protonated state for the inhibitor (or substrate) *α*‐amine, and its positive charge has been suggested to aid catalysis by stabilizing the planar anionic transition state (Richard & Amyes, 2001).

The distal amino acid of the inhibitor molecule is also predicted to be zwitterionic and also forms H‐bonds with highly conserved residues (Figure S7). The distal (side chain) amino group is within H‐bonding distance of Asn66 O^*δ*1^, Glu215 O^*ε*1^, and the backbone amide NH of Arg216, while the carboxylate forms key electrostatic interactions/H‐bonds with the side chain guanidinium of Arg216 (NH^*η*1^ and NH^*η*2^), as well as additional H‐bonds with the NH^δ2^ atoms of Asn66, Asn164, and Asn197.

**FIGURE 5:**
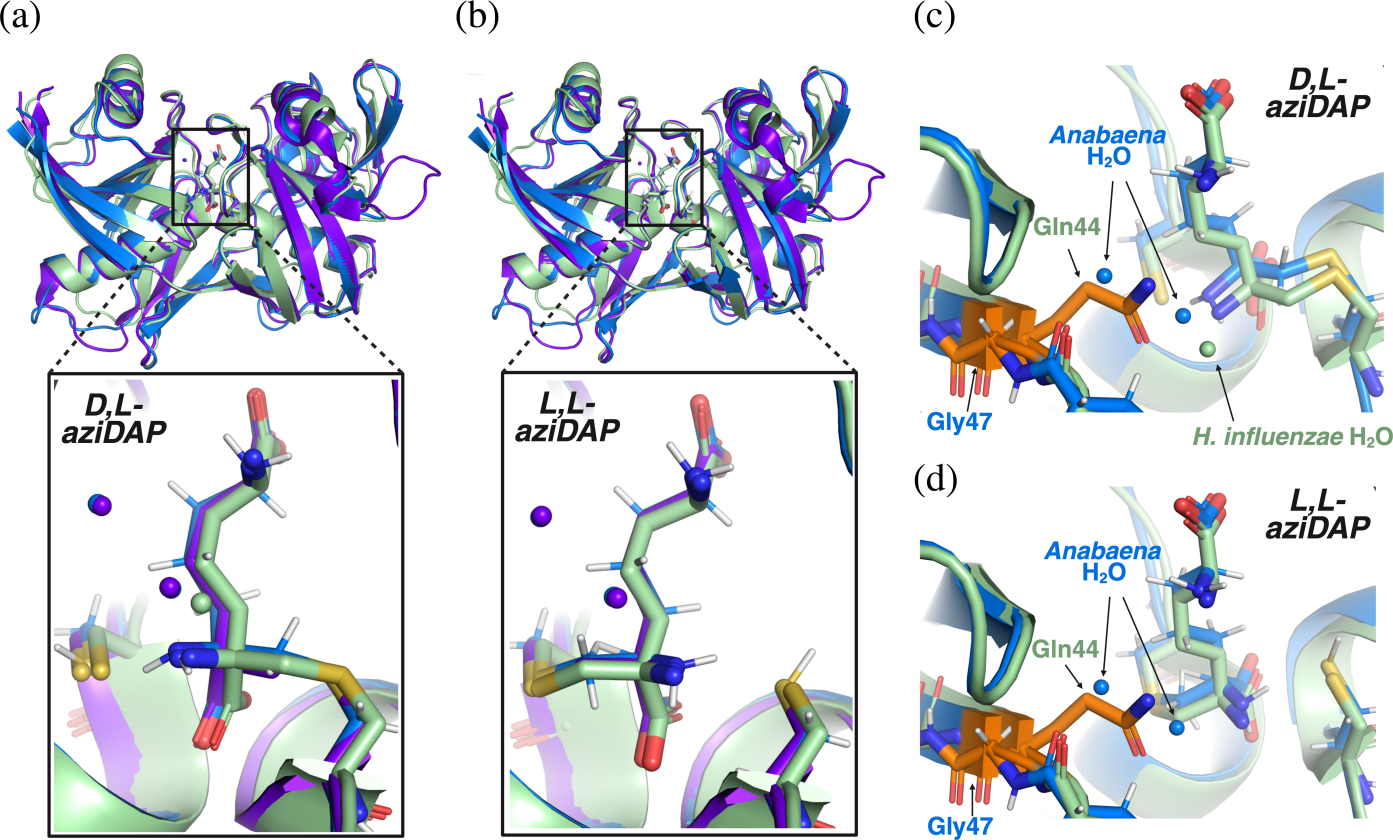
Binding of aziDAP in the active sites of *Anabaena*, *A. thaliana*, and *H. influenzae* DAP epimerases. (a) Overlay of crystal structures showing D,L‐aziDAP bound to *Anabaena* (blue, PDB ID 9MRO), *A. thaliana* (purple, PDB ID 3EKM), and *H. influenzae* (green, PDB ID 2GKJ) DapF enzymes. Water molecules are indicated with the appropriate color of the structure to which they correspond. (b) Overlay of crystal structures showing L,L‐aziDAP bound to *Anabaena* (blue, PDB ID 9MRP), *A. thaliana* (purple, PDB ID 3EJX), and *H. influenzae* (green, PDB ID 2GKE) DapF enzymes. (c) Position of water molecules in *Anabaena* (blue) and *H. influenzae* (green) DAP epimerase active sites when bound to D,L‐aziDAP. Aligned residues Gln44 (*H. influenzae*) and Gly47 (*Anabaena*) are indicated. (d) Position of water molecules in *Anabaena* (blue) DAP epimerase active site when bound to L,L‐aziDAP, overlayed with the structure of *H. influenzae* DapF bound to L,L‐aziDAP, which does not have any water molecules in the active site. Aligned residues Gln44 (*H. influenzae*) and Gly47 (*Anabaena*) are indicated.

The binding mode of D,L‐ and L,L‐aziDAP is highly conserved amongst the structures of *Anabaena*, *H. influenzae*, and *A. thaliana* DAP epimerases (Figure 5a, b). Interestingly, the side chain of aziDAP twists in nearly identical conformations in all three of these structures, and covalent interactions with catalytic cysteines are identical. This twisted conformation likely allows for appropriate positioning and proximity of the thiolate relative to the substrate *α*‐H, and may also create an effective orientation to maximize *π* orbital overlap between the carboxylate and planar anionic transition state (Corey & Sneen, 1956).

As is the case in other crystal structures of DapF in its closed, substrate/inhibitor‐bound conformation, *Anabaena* DapF has an active site that nearly excludes water. Water expulsion upon DapF closure around its substrate has been hypothesized to be one of the key factors that enable catalysis. Exclusion of water may cause a decrease in the dielectric constant of the active site, thereby resulting in a significant increase in thiol p*K*_a_ and facilitating deprotonation of the substrate *α*‐H (Mertz & Krishtalik, 2000). This effect can also be observed in non‐enzymatic systems, as the p*K*_a_ of 1‐butanethiol in acetonitrile is estimated to be ~28 (Ding et al., 2009). Interestingly, one of the main factors that differ between *Anabaena, A. thaliana*, and *H. influenzae* DapF structures is the presence of water molecules in the active site. In *Anabaena* and *A. thaliana* DapF, there are two water molecules in identical positions in crystal structures of these enzymes bound to either D,L‐ or L,L‐aziDAP (Figure 5a, b). In *H. influenzae* DapF bound to D,L‐aziDAP there is a single water molecule (Figure 5c), which is within H‐bonding distance to the proximal α‐amine of D,L‐aziDAP, the side chain amide of Gln44, and Asn11, and the side chain carboxylate of Glu208. In the structure of this enzyme bound to L,L‐aziDAP, there are no water molecules present in the active site (Figure 5d). The position of the water molecule in D,L‐aziDAP‐bound *H. influenzae* DapF is also slightly shifted compared to the water molecules found in *A. thaliana* and *Anabaena* enzymes. The difference in number and position of water molecules appears to be due to the presence of Gln44 in *H. influenzae* (Figure 5c, d), which is substituted for a Gly residue in the cyanobacterial and plant enzymes (Gly47 in *Anabaena* and Gly71 in *A. thaliana*, respectively). The lack of a side chain in the Gly‐containing enzymes creates space in the active site for water to occupy, but this space is taken up by the side chain of Gln44 in *H. influenzae* DapF. As Gln44 extends toward the center of the aziDAP molecule, this residue and the tighter active site may also explain why this enzyme does not tolerate lanthionine as a substrate.

### 2.3 Crystal structure of *Anabaena* DapF non‐covalently bound to D,L‐*α*‐methylDAP

To our surprise, two of four of the monomer units in the first crystal structure obtained after overnight incubation of *Anabaena* DapF with D,L‐aziDAP (PDB ID 9MRO) did not show a covalent bond between Cys224 and the methylene carbon of D,L‐aziDAP. Instead, a different molecule, D,L‐*α*‐methylDAP (Figure 3), was bound in the active site. This compound may have been formed as a byproduct during the final step of aziDAP synthesis (Figure S8), which involves a Li_(s)_, NH_3(l)_ reduction, followed by evaporation and addition of buffered water before incubation with the enzyme. The final two steps of the synthesis are completed without purification before addition of the enzyme due to the instability of the intermediates and final product. To confirm this result, a second crystal structure was solved (PDB ID 9MRV) that contained four monomers non‐covalently bound to D,L‐*α*‐methylDAP.

**FIGURE 6:**
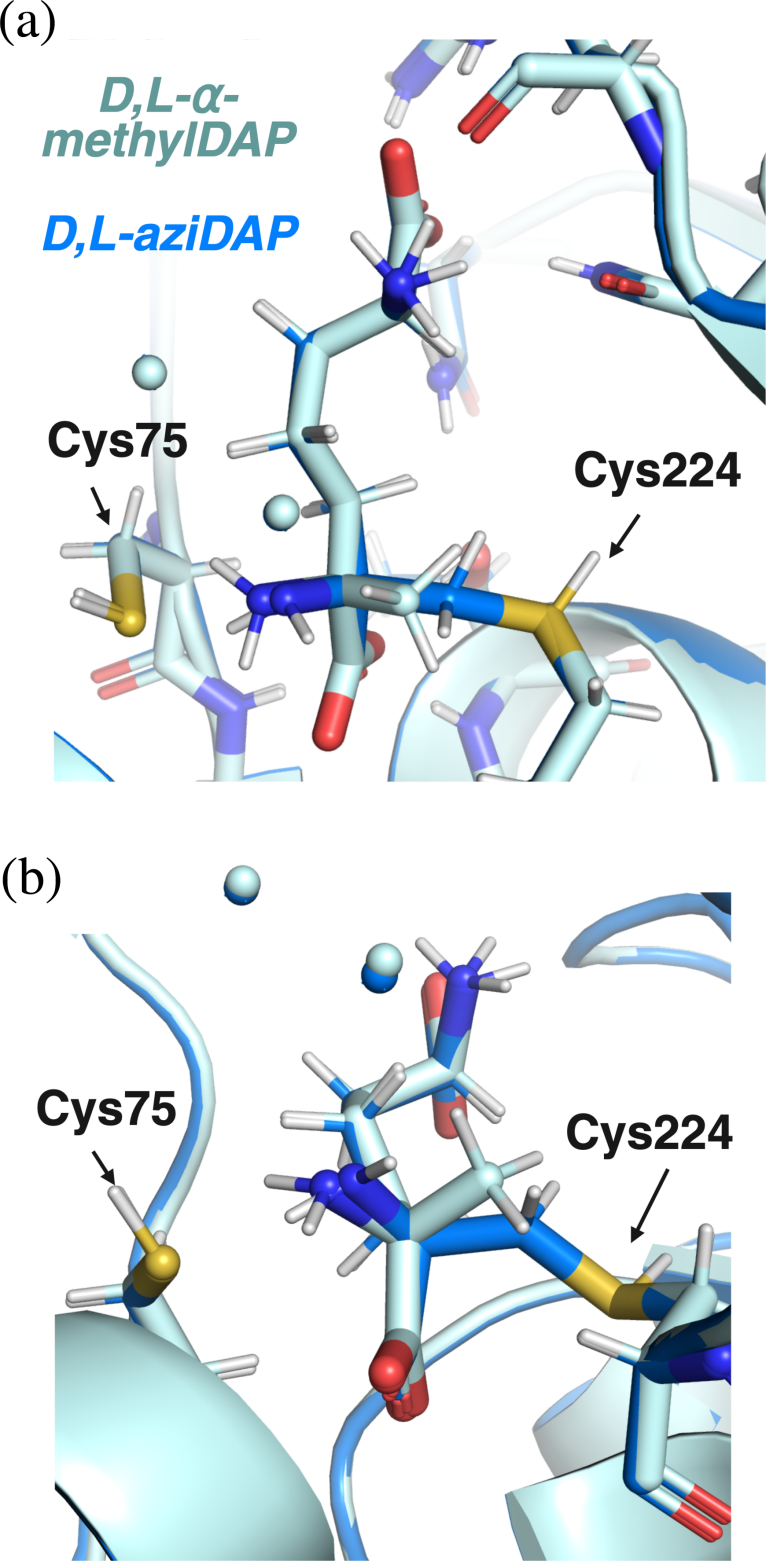
Overlay of *Anabaena* DapF covalently bound to D,L‐aziDAP (dark blue) and non‐covalently bound to D,L‐*α*‐methylDAP (light blue). (a) Overlay of inhibitors shows a high degree of alignment of side chain atoms. (b) Overlay of inhibitors shows slight movement of backbone N‐C^*α*^‐methyl(ene) atoms.

The structure of D,L‐*α*‐methylDAP non‐covalently bound to *Anabaena* DapF is very similar to that of the D,L‐aziDAP‐bound structure, with an RMSD of 0.160 Å over 280 C^*α*^ pairs (Figure 6). There is movement in the active site of the methyl carbon toward the amine (1.0 Å), relative to the position of the methylene carbon bound to Cys224 in the aziDAP structure. In fact, the N‐C^*α*^‐methylene bond angle in the aziDAP structure is 114°, while this same angle is only 95° in the D,L‐*α*‐methylDAP structure. The methyl carbon is 2.8 Å away from the Sγ atom of Cys224, indicating that the two are not covalently bound, as the theoretical C–S bond length is 1.8 Å (as is observed in the aziDAP‐bound structure). All other atoms in the inhibitors overlap nearly completely except for the proximal *α*‐amine, C^*α*^, and methyl/methylene carbons, and the H‐bonds formed between inhibitor and enzyme are identical in both structures. The water molecules observed in aziDAP‐bound *Anabaena* and *A. thaliana* DAP epimerases are also in the same positions (Figure 6b).

### 2.4 Synthesis of *α*‐methylated DAP analogues and inhibition studies

To test whether *α*‐methylDAP is a measurable inhibitor of DapF, we synthesized both L,L‐ and D,L‐*α*‐methylDAP stereoselectively (Figure S9). Synthesis of enantiomerically pure *α*‐substituted DAP has been previously reported (Paradisi et al., 2002), but we sought to establish a new route using chemistry previously reported by our group (based on work by Baran and coworkers). This route relies on decarboxylative radical addition of a protected glutamate to a cyclic dehydroalanine derivative for stereocontrolled synthesis of diamino diacids (Hsiao et al., 2021; Qin et al., 2017). We also produced a second set of methylated DAP analogues: (*S*)‐3‐((*S*)‐2‐amino‐2‐carboxyethoxy)‐2‐hydroxy‐2‐methylpropanoic acid and (*R*)‐3‐((*S*)‐2‐amino‐2‐carboxyethoxy)‐2‐hydroxy‐2‐methylpropanoic acid (L,L‐methyloxaDAP and D,L‐methyloxaDAP, respectively) via hydrogenation of the epoxide‐based inhibitors (Figure S4).

**FIGURE 7:**
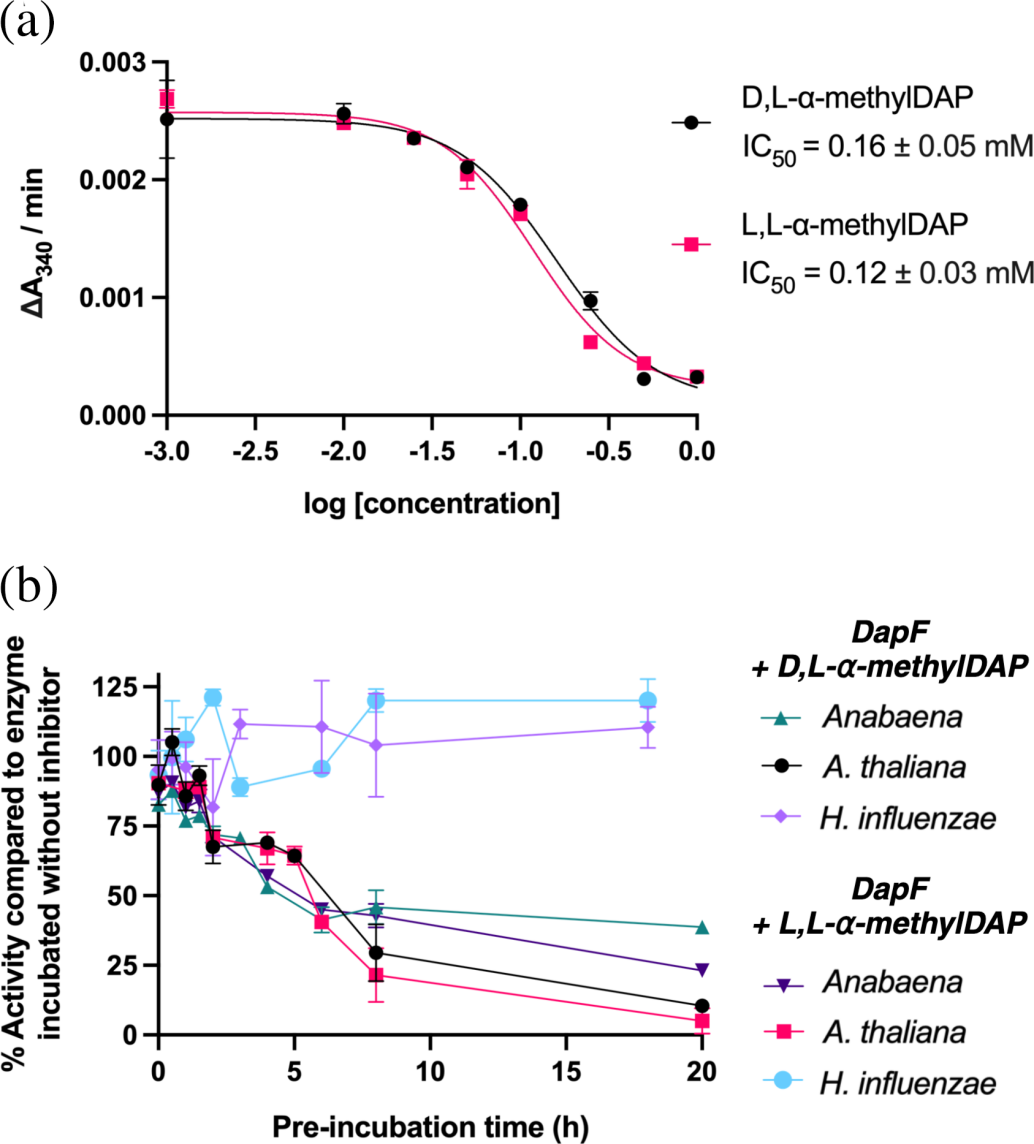
Slow‐binding inhibition of DapF by *α*‐methylDAP. (a) IC_50_ values for D,L‐ and L,L‐*α*‐methylDAP with *Anabaena* DAP epimerase after 20 h incubation. (b) Inhibition of various DAP epimerases by 250 μM D,L‐ or L,L‐*α*‐methylDAP after varying lengths of pre‐incubation of enzyme and inhibitor. Activity of each enzyme after incubation with inhibitor was scaled relative to activity of each enzyme incubated for the same time length with water only.

With four methylated DAP analogues in hand (Figure 3), these compounds were first confirmed to not be substrates or inhibitors of DAP dehydrogenase in concentrations up to 5 mM (Figure S10). We then proceeded to test inhibition of DapF in the DAP dehydrogenase–coupled kinetic assay. Interestingly, there was no inhibition of *Anabaena* DapF when the kinetic assay was performed immediately after mixing inhibitor and enzyme. However, inhibition was observed after overnight incubation of *Anabaena* DapF with each compound (as was done for protein crystallization), suggesting these methylated DAP analogues are slow‐binding inhibitors of DapF. L,L‐*α*‐MethylDAP and D,L‐*α*‐methylDAP were found to have IC_50_ values in the mid‐micromolar range (0.12 and 0.16 mM, respectively; Figure 7a). Methylated compounds L,L‐methyloxaDAP and D,L‐methyloxaDAP also showed inhibition of *Anabaena* DapF after overnight incubation, but were less potent with IC_50_ values greater than 0.2 mM (Figure S11), and were not further investigated.

To measure the time‐dependency of inhibition, DAP epimerase from *Anabaena* was incubated with L,L‐*α*‐methylDAP and D,L‐*α*‐methylDAP for increasing lengths of time and then measured for enzyme activity (Figure 7b). Compared to a control sample of enzyme incubated for the same time length without inhibitor added, activity decreased over time and appeared to reach an equilibrium after 8 h of pre‐incubation. The same experiment was repeated with DAP epimerases from *A. thaliana* and *H. influenzae* to test whether this effect was generalizable. Similar to the results of the lanthionine substrate tolerance experiment, *A. thaliana* DapF behaved similarly to *Anabaena* DapF and activity decreased with increasing length of pre‐incubation. However, DapF from *H. influenzae* did not show the same time‐dependent effect and did not appear to be inhibited by either inhibitor at 250 μM. This may be due to the tighter conformation of the enzyme active site and the presence of Gln44 rather than a glycine residue, which may also explain this enzyme's reduced activity with lanthionine as a substrate.

## 3 DISCUSSION

Racemases and epimerases are by nature unusual enzymes as they must be able to bind two stereoisomers of their substrate—enantiomers for racemases and diastereomers for epimerases. This feature sets them apart from most other enzymes, which typically operate with rigid stereospecificity. For cofactor‐independent racemases and epimerases with the DapF‐like fold, including DAP epimerase from *Anabaena* studied here, binding and turnover of both substrate stereoisomers is enabled by an unusual pseudosymmetric structure (Bearne, 2020). These enzymes have been suggested to have arisen from a gene duplication event, accounting for the structural pseudosymmetry but low sequence identity of their N‐ and C‐terminal domains (L. Liu et al., 2002). This overall structure results in an active site at the cleft between the two domains that performs a reversible stereoisomerization reaction. This reversibility poses a challenge for the development of these enzymes as biocatalysts for the production of non‐proteinogenic D‐amino acids—a so far unrealized goal but an exciting and relevant endeavor in the field of enzyme engineering (Alfonzo et al., 2022; Blaskovich, 2016; Wang et al., 2024).

The ability of *Anabaena* and *A. thaliana* DAP epimerases to tolerate lanthionine as a substrate was unexpected but not unprecedented, as there is some indirect evidence to suggest that DapF can epimerize lanthionine. Richaud et al. showed in 1993 that crude lysate of *E. coli* epimerized L,L‐lanthionine into D,L‐lanthionine, but this activity was absent from the lysate of a Δ*dapF* strain (Richaud et al., 1993). They also showed that Δ*dapF E. coli* can incorporate D,L‐lanthionine into their peptidoglycan instead of D,L‐DAP. More recently, Darbyshire et al. tested lanthionine as a substrate of a predicted DapF enzyme from *Fusobacterium nucleatum*, an organism that uses D,L‐lanthionine in its peptidoglycan instead of D,L‐DAP (Darbyshire et al., 2023; Fredriksen et al., 1991). However, the authors found an extremely low turnover rate with lanthionine and concluded that this enzyme likely has some other physiological substrate, which our group later identified as histidine (Lamer et al., 2024).

To the best of our knowledge, this is the first time lanthionine epimerization has been observed with a purified DAP epimerase, and comparison of crystal structures of DapF from *Anabaena*, *A. thaliana*, and *H. influenzae* suggest that subtle difference in architecture of DapF enzymes can influence substrate binding and catalysis. The enzymes used in this study all rely on the same conserved active site residues to form H‐bonds with aziDAP inhibitors (Figure S1). One of the only differences between active sites amongst these three DAP epimerases is Gln44 in *H. influenzae* versus Gly47 in *Anabaena* and Gly71 *in A. thaliana* (Figure 5). In *H. influenzae* DapF, the side chain of Gln44 extends toward the center of the inhibitor molecule. In *Anabaena* and *A. thaliana* DAP epimerases, the lack of a side chain in Gly47 and Gly71 allows two water molecules to occupy the space taken up by Gln44's side chain in *H. influenzae* DapF. These water molecules are in identical positions in all structures of *Anabaena* and *A. thaliana* enzymes, but are absent in the *H. influenzae* structure (*H. influenzae* DapF bound to D,L‐aziDAP does have a single water molecule in the active site, but in a different position). Gln44 may create a smaller active site pocket that is unable to tolerate even small changes in substrate structure, providing a possible explanation for functional differences in tolerance of lanthionine as a substrate and inhibition by *α*‐methylDAP compounds.

While most residues forming the active site pocket of the DAP epimerases studied here are highly conserved, there is one region of the active site that varies amongst the enzymes studied. In *Anabaena* and *A. thaliana*, the loop curving around the substrate connecting *β*‐strand 4 with *α*‐helix 2 has the sequence Pro‐Glu‐Met (Figure S12), while in *H. influenzae* it is Val‐Ser‐Gln. These residues are not involved in direct H‐bonding with the inhibitor/substrate; however, the Glu residue in the plant and cyanobacterial enzymes forms an electrostatic interaction with Arg216 of *Anabaena* DapF (Arg246 in *A. thaliana*). This key Arg residue interacts with both the distal carboxylate and amine of the DAP molecule and has been previously suggested to be important for chiral recognition of an L‐stereocenter of the distal C^*α*^ of D,L‐ and L,L‐DAP, providing an explanation as to why DapF does not turnover D,D‐DAP. The salt bridge formed between glutamate and arginine in *Anabaena* and *A. thaliana* DAP epimerases may help to seal the active site and strengthen interactions with the substrate, providing a possible explanation for the reduced *K*_M_ of *Anabaena* and *A. thaliana* DAP epimerases with L,L‐DAP compared to that of *H. influenzae* (Table 1).

While aziridine‐based inhibitors have been successfully used to covalently inhibit and obtain crystal structures of DapF in its closed conformation, the synthesis is complex and not generalizable to other amino acid substrates. Interestingly, *α*‐methyl glutamate and aspartate have been previously tested as inhibitors of glutamate racemase and aspartate racemase, respectively, with no inhibition observed (Diven, 1969; Yamashita et al., 2004). However, neither of these reports mentioned a pre‐incubation period between enzyme and inhibitor, and so it may be possible that the time‐dependency of this interaction has been overlooked. Slow‐binding inhibition occurs when equilibrium between an enzyme, an inhibitor, and the enzyme‐inhibitor (EI) complex is established slowly, necessitating a pre‐incubation period to establish this equilibrium before enzyme activity measurement. In the case of *α*‐methylDAP inhibitors and DAP epimerase, this slow‐binding phenomenon may be due to the buried, highly ordered nature of the enzyme active site and the “oyster shell‐like clamping” movement required to form the closed conformation EI complex (Pillai et al., 2006). Slow‐binding *α*‐methylated amino acid inhibitors may be a useful alternative to facilitate crystallization and structural elucidation of other enzymes of the cofactor‐independent racemase and epimerase family. The success of these compounds will hinge on whether the active site of a particular enzyme of interest has enough room to accommodate the inhibitor, as is the case in *A. thaliana* and *Anabaena* DAP epimerases. Solving crystal structures of these fascinating enzymes in their closed, active conformation is not a trivial pursuit, and new approaches are needed to improve structural understanding of these enzymes for advances in both antibiotic development and enzyme engineering.

## 4 CONCLUSIONS

Crystal structures of DAP epimerase from the cyanobacteria *Anabaena* were solved, revealing this enzyme in its closed, active conformation. Two structures show the enzyme covalently bound to synthesized aziridine‐based inhibitors L,L‐ and D,L‐aziDAP, but one showed the enzyme non‐covalently bound to D,L‐*α*‐methylDAP, a presumable synthetic byproduct. D,L‐ and L,L‐*α*‐methylDAP were synthesized stereoselectively and shown to be slow‐binding inhibitors of DAP epimerase. Functional and structural comparisons of DAP epimerase from *Anabaena, A. thaliana*, and *H. influenzae* show subtle differences in the active sites, which result in changes in function, including substrate and inhibitor tolerance. Future studies may investigate *α*‐methylated amino acids as inhibitors of other members of the cofactor‐independent racemase and epimerase family, potentially providing a new strategy to aid structural elucidation of these enzymes, several of which have never been solved in their closed, active conformations.

## 5 MATERIALS AND METHODS

### 5.1 Molecular biology

DNA coding sequences for *Anabaena* sp. YBS01 DAP epimerase (GenBank ID: CP034058.1; UniProt ID: A0A5Q0GFR1) with a C‐terminal hexahistidine tag, *H. influenzae* ATCC 51907 DAP epimerase (GenBank ID: L42023.1; Uniprot ID: P44859), and *Symbiobacterium thermophilum* IAM 14863 DAP dehydrogenase (GenBank ID: AP006840.1; UniProt ID: Q67PI3) with an N‐terminal hexahistidine tag were codon optimized for expression in *E. coli* using Integrated DNA Technologies Codon Optimization Tool. Genes were each synthesized by GenScript and inserted into pET‐24b(+) cloning vectors using a 5′ *Nde*I and a 3′ *Hind*III restriction site. *E. coli* BL21(DE3) cells (New England BioLabs) were transformed with the appropriate plasmids according to manufacturer instructions, and cultures were stored as 20% glycerol stocks at −80°C. *A. thaliana* DAP epimerase (GenBank ID: AL132966; UniProt ID: Q9LFG2) was expressed from a previously described cloning vector in *E. coli* M15(pREP4) cells (Pillai et al., 2009).

### 5.2 Protein expression and enzyme purification

Frozen glycerol stocks of *E. coli* BL21(DE3) cells transformed with the appropriate pET‐24b(+) vector containing the *Anabaena* DapF gene or *H. influenzae* DapF gene were inoculated into 50 mL of sterile Difco™ LB Broth, Miller (Luria‐Bertani) media with kanamycin (50 μg/mL) as selective pressure. The cells were grown overnight at 37°C with shaking at 240 rpm. The next day, 20 mL of the overnight culture was added to 500 mL of sterile LB media with kanamycin added, and the cells were grown to an optical density (OD_600_) of ~0.8 at 37°C. Protein expression was then induced at 37°C by the addition of isopropyl‐*β*‐D‐1‐thiogalactopyranoside (IPTG; Chem‐Impex International) to a final concentration of 0.5 mM, and flasks were vigorously shaken for an additional 4 h. Cells were then harvested by centrifugation (5000 × *g*, 10 min, 4°C) and the pellets were stored at −80°C. *S. thermophilum* DAP dehydrogenase was expressed as previously described (Gao et al., 2012) and generally according to the above procedure, except the final concentration of IPTG used was 0.1 mM, and the cells were grown for 6 h at 37°C after induction. *A. thaliana* DAP epimerase was expressed and purified as previously described (Pillai et al., 2009).

Frozen cell pellets were resuspended (30 mL buffer/500 mL culture media) evenly in ice cold lysis buffer (50 mM NaH_2_PO_4_, 300 mM NaCl, 5 mM imidazole, pH 8.0) and lysed by sonication while kept on ice. Lysis buffer for the DAP dehydrogenase had 5% glycerol added. DNase I (Thermo Fisher Scientific, 1 U) was added and the lysate kept on ice for 30 min with occasional inversion. The cellular debris was removed by centrifugation (20,000 × *g*, 30 min, 4°C), and then Ni‐NTA resin (McLab, 3 mL) was added to the clarified supernatant and the mixture was gently shaken for 1 h at 4°C. The mixture was then loaded onto a fritted column and the flow through collected at 4°C. The resin was washed with 25 mL of lysis buffer containing 20 mM imidazole, and then the protein was eluted in 5 mL fractions by sequential addition of elution buffer (50 mM NaH_2_PO_4_, 300 mM NaCl, pH 8) containing 40, 60, 80, 100, 200, and then 500 mM imidazole. Eluted fractions were analyzed by SDS‐PAGE and the samples containing the protein of interest were pooled together and then loaded on a Sephadex G‐15 size exclusion column (50 mM NaH_2_PO_4_, 150 mM NaCl, pH 8 for enzyme assays or 20 mM Tris, 100 mM NaCl, 2 mM DTT, pH 8 for crystallography; 5.0 × 15.0 cm). Elution of the protein of interest was monitored by absorbance at 280 nm, and then fractions were pooled and concentrated using an Amicon® Ultra Centrifugal Filter, 10 kDa MWCO (5000 × *g*, 4°C). Protein concentration was measured by absorbance at 280 nm using a Nanodrop spectrophotometer. Aliquots of the enzyme solutions were stored at −80°C, and the dehydrogenase was stored with 10% glycerol added.

### 5.3 DapF ^1^H‐NMR substrate testing and kinetic assays

To screen potential substrates for *Anabaena* DapF, 5 mM solutions of amino acids were prepared in D_2_O phosphate buffer (600 μL, 20 mM NaH_2_PO_4_, 0.1 mM 2‐mercaptoethanol, pD 7.3) at room temperature. To initiate the reaction, enzyme diluted in D_2_O phosphate buffer (~2 μg) was added. The ^1^H‐NMR spectrum was recorded at given times from the addition of the enzyme (typically at ~15 min and again at 48 h). Spectra were analyzed for the disappearance of the *α*‐H compared to that of a negative control without added enzyme, prepared from the same original amino acid solution. NMR spectra were collected on a Varian 600 MHz NMR spectrometer.

To compare the relative rates of DapF enzymes with DAP or lanthionine, DAP epimerase was added to either a solution of 5 mM L,L‐DAP or 5 mM L,L‐lanthionine in deuterated phosphate buffer (20 mM sodium phosphate, 100 mM NaCl, pH 8 in D_2_O) in a 2‐mm (i.d.) NMR tube. The tube was mixed, and time‐coursed ^1^H‐NMR spectra were recorded at 27°C on a 600 MHz NMR instrument every 40 s. The experiments were performed in duplicate for each enzyme with each substrate. An ^1^H‐NMR spectrum of the substrate without adding enzyme was recorded as a control to obtain an average integration value for the *α*‐H signal of each substrate. The integration of the *α*‐H signal over time was divided by the value of the *α*‐H signal with no enzyme added for each time point. These values were divided by the mmol of substrate and plotted against time using GraphPad Prism. The values shown are the average of the mean.

### 5.4 DAP dehydrogenase coupled enzyme assays

Enzyme assays were conducted according to a modified literature procedure (Lam et al., 1988). The coupled enzyme assay was performed at 27°C in buffer containing 20 mM Tris, 100 mM NaCl, 1 mM EDTA, 1 mM DTT, pH 8 in a 96‐well plate to a final volume of 200 μL. DAP epimerase activity was assayed at 340 nm using a SpectraMax i3x plate reader (Molecular Devices) by coupling the conversion of L,L‐DAP to D,L‐DAP with the NADP^+^‐dependent DAP dehydrogenase‐catalyzed (*S. thermophilus*) oxidation of D,L‐DAP. The assay solution contained varying concentrations of LL‐DAP, 300 μM NADP^+^, ~2 μg of DAP dehydrogenase, and ~15 ng of DAP epimerase (and varying concentrations of inhibitors if used). Control experiments were first conducted with each DAP epimerase enzyme to ensure that the epimerization reaction was the rate‐limiting reaction. Kinetic experiments that provided *K*_M_ and *k*_cat_ values were completed in triplicate, and errors displayed are averages of the mean. The initial linear range was used to perform Michaelis–Menten analysis in GraphPad Prism. All other kinetic experiments were completed in duplicate.

Enzyme assays completed with potential inhibitors were first tested to confirm that these compounds were not substrates for DAP dehydrogenase. To do so, a reaction mixture for the oxidative deamination of D,L‐DAP (or appropriate DAP analogue) was prepared containing final concentrations of 250 μM D,L‐DAP (or up to 5 mM DAP analogue), 300 μM NADP^+^, and DAP dehydrogenase to a final volume of 200 μL in a 96 well plate. Additionally, compounds were tested to confirm that they did not inhibit DAP dehydrogenase. To do so, the same reaction mixture was set up, but including both 100 μM D,L‐DAP and the potential inhibitor in concentrations up to 5 mM. Oxidative deamination of D,L‐DAP in the presence of the inhibitor, DAP dehydrogenase, and NADP^+^ was assayed and compared to the positive control with no inhibitor added to ensure DAP dehydrogenase was not affected. To test inhibition of DAP epimerases, DAP dehydrogenase‐coupled reactions were set up either with no incubation time between inhibitor and DapF as described above, or with a specified pre‐incubation period of the inhibitor with DapF at room temperature in the 96‐well plate (lid on) before addition of excess DAP dehydrogenase, NADP^+^ (300 μM), and L,L‐DAP (150 μM). For IC_50_ experiments, enzyme and inhibitor were incubated overnight at room temperature for 20 h. IC_50_ values were calculated using GraphPad Prism. For time‐dependent inhibition assays, enzyme and inhibitor were incubated at room temperature for a specified length of time before adding DAP dehydrogenase, NADP^+^, and L,L‐DAP and measuring absorption at 340 nm. The initial linear rate was compared to the initial linear rate of enzyme incubated without inhibitor (water added instead) to obtain a % activity value for each time point.

### 5.5 Circular dichroism

Circular dichroism experiments were performed using an OLIS globalworks CD spectrophotometer. All reactions were monitored using a quartz cuvette with a 0.2 mm pathlength at 25°C. The maximum CD signal was obtained for L,L‐DAP and L,L‐lanthionine at 203 nm, and all reactions were monitored at this wavelength. The average integrated CD signal (in mdeg) was recorded every second. Reactions were conducted in phosphate buffer (20 mM NaH_2_PO_4_, 100 mM NaCl, pH 8).

### 5.6 X‐ray crystallography

For crystallization of DapF bound to L,L‐ or D,L‐aziDAP, freshly prepared inhibitor was used without purification over the last two steps (assumed 30% yield overall for ester hydrolysis and reduction). The inhibitor was dissolved in buffer (20 mM Tris, 100 mM NaCl, pH 8) and pH was carefully adjusted if needed with dilute HCl or NaOH to a final concentration of ~3 mM. The inhibitor (~2 μmol, ~10× molar excess relative to the enzyme) was then added to a solution of buffered enzyme (~0.2 μmol) to a final concentration of 0.6 mM inhibitor and 0.05 μM enzyme (1.5 mg/mL). The solution was incubated for 24 h at room temperature before concentrating the enzyme using an Amicon® Ultra Centrifugal Filter, 10 kDa MWCO (5000 × *g*, 4°C) to a final concentration of 40 mg/mL for crystallization.

Crystallization conditions were screened by mixing 0.5 μL protein with 0.5 μL reservoir using the Phoenix ARI crystallization Robot (Art Robbins Instruments, USA), and crystals were grown at 18°C in a sitting‐drop plate. Crystals of DapF bound to D,L‐aziDAP appeared in the condition of 0.1 M succinic acid pH 7.0, 15% w/v polyethylene glycol 3350, while crystals of DapF bound to L,L‐aziDAP appeared in the condition of 0.1 M phosphate/citrate 4.2, 40% v/v PEG 300. The crystals were flash frozen in liquid nitrogen after being passed through 20% glycerol as a cryoprotectant. The data was collected at Stanford Synchrotron Radiation Light Source (SSRL), at beamline 12–1 1 and Canadian Light Source (CLS), at beamline CFCM‐BM. The data was processed using the program XDS (Kabsch, 2010). The molecular placement was done by the program CCP4 phaser MR (Agirre et al., 2023; McCoy et al., 2007) using a structure prediction by AlphaFold as a searching model (Bryant et al., 2022). The structures were refined using Phenix (Adams et al., 2010) and modified manually in Coot (Emsley et al., 2010). Data statistics and PDB IDs are summarized in Table S3.

### 5.7 Sequence and structural analysis

Protein sequence alignments were performed using UniProt Align, and structural alignments were performed using RSCB PDB Pairwise Structure Alignment Tool (Bittrich et al., 2024). To generate sequence alignments including secondary structure elements, protein sequences were aligned using UniProt Align, and then secondary structure elements from the DapF crystal structure (PDB 9MRO) were overlaid with this sequence alignment using ESPript 3.0 (Robert & Gouet, 2014). Protein structural alignments were completed using PyMOL.

## Supporting information


**Data S1:** Supporting Information

## Data Availability

The data that support the findings of this study are openly available in the RCSB PDB at https://www.rcsb.org, reference numbers 9MRO, 9MRV, and 9MRP.
