# Supplementary material for: Structure and inhibition of diaminopimelic acid epimerase by slow‐binding α‐methyl amino acids
Source: Protein Sci. 2025 Apr 29;34(5):e70139. doi: 10.1002/pro.70139 (PMC12039745; doi:10.1002/pro.70139)
Supplement: Supplementary file 1 — Data S1: Supporting Information [file PRO-34-e70139-s001.docx]

***Supporting Information for “Structure and inhibition of diaminopimelic acid epimerase by slow-binding α-methyl amino acids”***

Tess Lamer^1^, Pu Chen^2,3^, Karizza Catenza^1^, Ilia Perov^1^, Bethan Donnelly^1^, Yu-Ting Hsiao^1^, Tayla J. Van Oers^1^, M. Joanne Lemieux^2,3^, and John C. Vederas^1^*

^1^ University of Alberta, Department of Chemistry, Edmonton, AB, Canada

^2^ University of Alberta, Department of Biochemistry, Edmonton, AB, Canada

^3^ Li Ka Shing Institute of Virology, University of Alberta, Edmonton, AB, Canada

* Indicates corresponding author. Correspondence should be directed to [john.vederas@ualberta.ca](mailto:john.vederas@ualberta.ca)

Table of Contents

[Table S1 3](#_Toc187961300)

[Table S2 9](#_Toc187961301)

[Table S3 11](#_Toc187961302)

[Figure S1 12](#_Toc187961303)

[Figure S2 13](#_Toc187961304)

[Figure S3 14](#_Toc187961305)

[Figure S4 15](#_Toc187961306)

[Figure S5 16](#_Toc187961307)

[Figure S6 17](#_Toc187961308)

[Figure S7 18](#_Toc187961309)

[Figure S8 19](#_Toc187961310)

[Figure S9 20](#_Toc187961311)

[Figure S10 21](#_Toc187961312)

[Figure S11 22](#_Toc187961313)

[Figure S12 23](#_Toc187961314)

[Supporting information methods 24](#_Toc187961315)

[Protein and DNA sequences 24](#_Toc187961316)

[Synthetic chemistry methods 28](#_Toc187961317)

[Synthesis of D,L-epoDAP and L,L-epoDAP 28](#_Toc187961318)

[Synthesis of D,L-methyloxaDAP and L,L-methyloxaDAP (Figure S4) 32](#_Toc187961319)

[Synthesis of D,L-α-methylDAP and L,L-α-methylDAP 33](#_Toc187961320)

Table S1**. Inhibitors tested against DapF in this and previous studies.** When stereochemistry is not specified, compounds were tested as a mixture of all stereoisomers.

| **Structure** | **DapF tested** | ***K*_i_ or IC_50_** | **Inhibitor pre-incubation time** | **Reference** |
| --- | --- | --- | --- | --- |
| 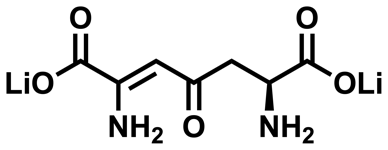 | *E. coli* | IC_50_ 500 µM | Not mentioned | Caplan, J. F., Zheng, R., Blanchard, J. S., & Vederas, J. C. (2000). Organic Letters, 2(24), 3857–3860. |
| 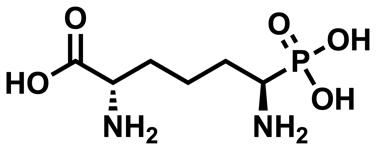 | *E. coli* | *K*_i_ 6.2 mM | Tested time-dependent inactivation by preincubation at concentrations up to 2.25 mM for up to 2 h; no effect observed | Song, Y., Niederer, D., Lane-Bell, P. M., Lam, L. K. P., Crawley, S., Palcic, M. M., Pickard, M. A., Pruess, D. L., & Vederas, J. C. (1994). Journal of Organic Chemistry, 59(19), 5784–5793. |
| 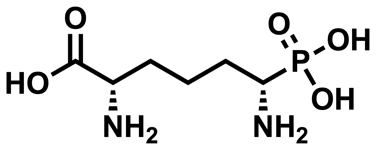 | *E. coli* | *K*_i_ 3.9 mM | ’’ | Song *et al.* |
| 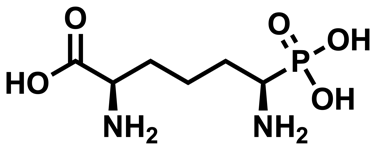 | *E. coli* | No inhibition | ’’ | Song *et al.* |
| 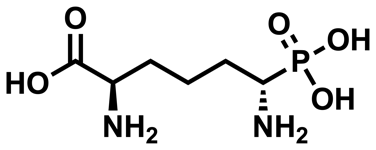 | *E. coli* | *K*_i_ 7.2 mM | ’’ | Song *et al.* |
| 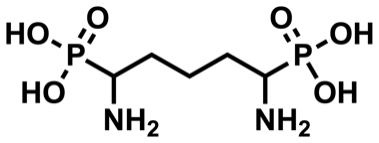 | *E. coli* | *K*_i_ 14 mM | ’’ | Song *et al.* |
| 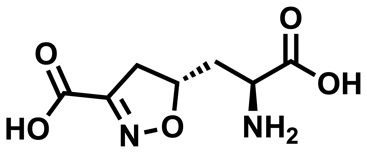 | *E. coli* | Competitive inhibition: 4%  at 0.5 mM | Tested time-dependent inactivation by preincubation at concentrations up to 3 mM for up to 2 h; no effect observed | Abbott, S. D., Lane-Bell, P., Sidhu, K. P. S., & Vederas, J. C. (1994). Journal of the American Chemical Society, 116(15), 6513–6520. |
| 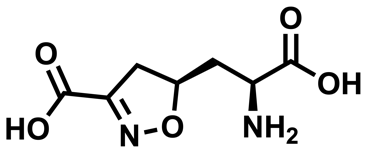 | *E. coli* | Competitive inhibition: 18%  at 1 mM | ’’ | Abbott *et al.* |
| 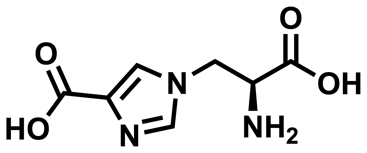 | *E. coli* | Competitive inhibition: 18%  at 1 mM | ’’ | Abbott *et al.* |
| 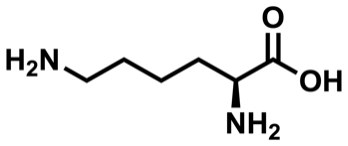 | *E. coli* | *K*_i_ > 2.4 mM | ’’ | Abbott *et al.* |
| 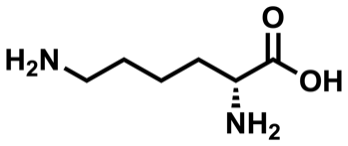 | *E. coli* | *K*_i_ > 2.4 mM | ’’ | Abbott *et al.* |
| 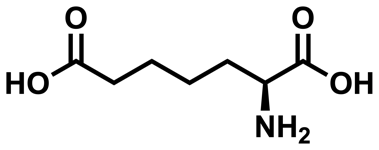 | *E. coli* | *K*_i_ > 2.4 mM | Tested time-dependent inactivation by preincubation at concentrations of 100 µM for up to 2 h; no effect observed | Abbott *et al.* |
| 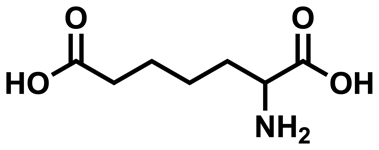 | *E. coli* | *K*_i_ > 2.4 mM | ’’ | Abbott *et al.* |
| 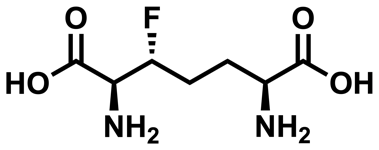 | *E. coli* | IC_50_ 25 µM | Not done – turned over by enzyme and product is not an effective inhibitor | Gelb, M. A., Lin, Y., Pickard, M. A., Song, Y., Vederas, J. C. (1990). Journal of the American Chemical Society, 112, 4932–4942. |
| 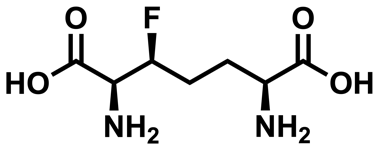 | *E. coli* | IC_50_ 8 µM | ’’ | Gelb *et al.* |
| 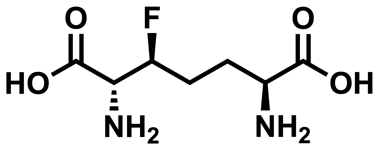 | *E. coli* | IC_50_ 4 µM | ’’ | Gelb *et al.* |
| 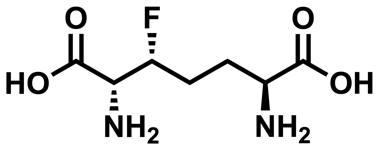 | *E. coli* | IC_50_ 10 µM | ’’ | Gelb *et al.* |
| 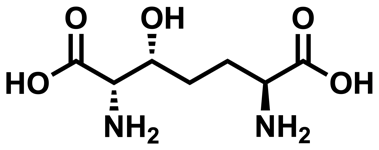 | *E. coli* | IC_50_ 2500 µM | ’’ | Gelb *et al.* |
| 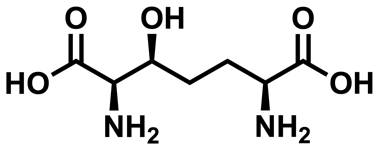 | *E. coli* | IC_50_ 4000 µM | ’’ | Gelb *et al.* |
| 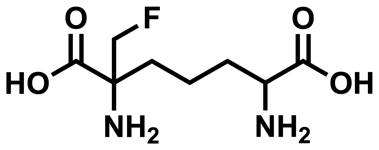 | *E. coli* | N.d.; spontaneous conversion to aziridine | Tested time-dependent inactivation by preincubation at concentrations up to 250 µM for up to 40 min; time-dependent inhibition observed | Gerhart, F., Higgins, W., Tardif, C., & Ducep, J. B. (1990). Journal of Medicinal Chemistry, 33(8), 2157—2162. |
| 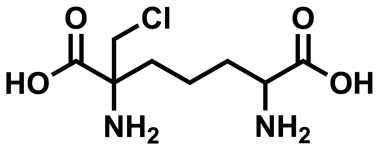 | *E. coli* | N.d.; spontaneous conversion to aziridine | ’’ | Gerhart *et al.* |
| 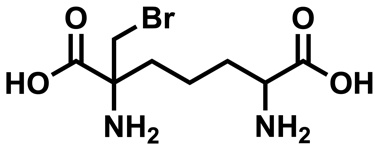 | *E. coli* | N.d.; spontaneous conversion to aziridine | ’’ | Gerhart *et al.* |
| 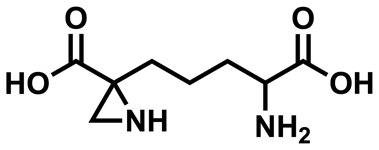 | *E. coli* | Could not be determined, but 5.5 µM was calculated for *K*_i_/*k*_i_ | Tested time-dependent inactivation by preincubation at concentrations up to 2 µM for up to 30 min; time-dependent inhibition observed | Gerhart *et al.* AND Higgins, W., Tardif, C., Richaud, C., Krivanek, M. A., & Cardin, A. (1989). European Journal of Biochemistry, 186(1–2), 137–143. |
| 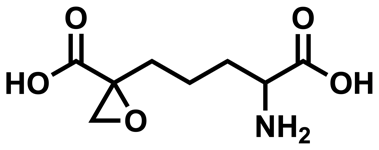 | *H. influenzae* | Could not be determined due to instability at pH 7 | N/A | Cox, R. J., Durston, J., & Roper, D. I. (2002). Journal of the Chemical Society, Perkin Transactions 1, 8(8), 1029–1035. |
| 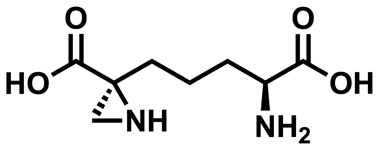 | *H. influenzae* | Not determined, but irreversible inhibition observed |  | Diaper, C. M., Sutherland, A., Pillai, B., James, M. N. G., Semchuk, P., Blanchard, J. S., & Vederas, J. C. (2005). Org. Biomol. Chem., 3(24), 4402–4411. |
| 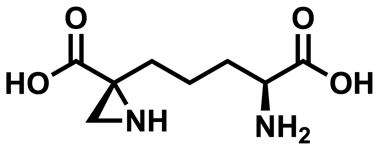 | *H. influenzae* | Not determined, but irreversible inhibition observed | Tested time-dependent inactivation by incubation for 16 h at room temperature | Diaper *et al.* |
| 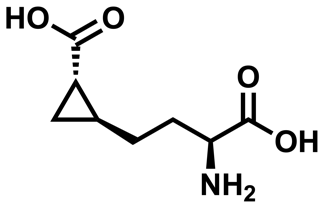 | *H. influenzae* | Tested as a mixture of diastereomers, IC_50_ >2.88 mM | Not mentioned | Diaper *et al.* |
| 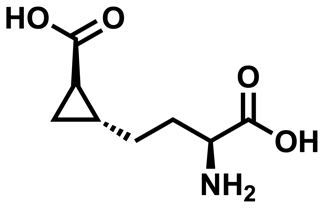 | *H. influenzae* | IC_50_ 2.88 mM | Tested time-dependent inactivation, not indicated whether slow-binding effect observed | Diaper *et al.* |
| 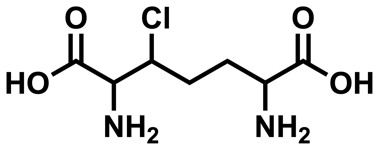 | *E. coli* | *K*_i_ 0.2 µM | Time-dependent inactivation observed, but inhibition decreases over time as the enzyme turns over the compound | Baumann, R. J., Bohme, E. H., Wiseman, J. S., Vaal, M., & Nichols, J. S. (1988). Antimicrobial Agents and Chemotherapy, 32(8), 1119–1123. |
| 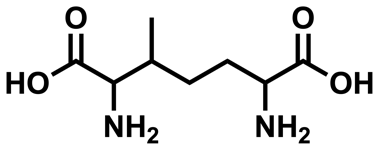 | *E. coli* | *K*_i_ >10 mM | Time-dependent inactivation tested but not observed, concentrations and time not indicated | Baumann *et al.* |
| 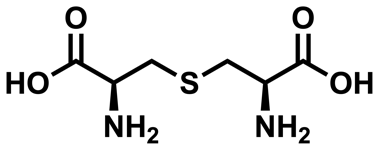 | *E. coli* | *K*_i_ 0.18 mM | Tested time-dependent inactivation by preincubation at concentrations up to 4 mM for up to 60 min; no time-dependent inhibition observed | Lam, L. K., Arnold, L. D., Kalantar, T. H., Kelland, J. G., Lane-Bell, P. M., Palcic, M. M., Pickard, M. A., & Vederas, J. C. (1988). Journal of Biological Chemistry, 263(24), 11814–11819. |
| 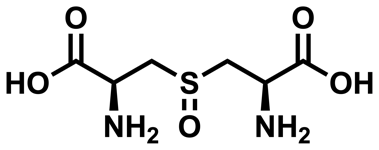 | *E. coli* | *K*_i_ 11 mM | ’’ | Lam *et al.* |
| 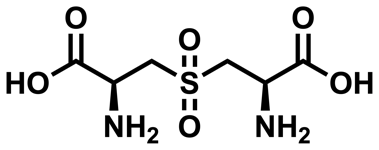 | *E. coli* | *K*_i_ 21 mM | ’’ | Lam *et al.* |
| 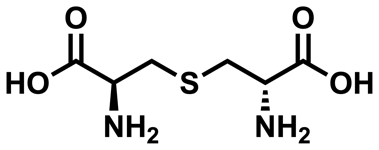 | *E. coli* | *K*_i_ 0.42 mM | ’’ | Lam *et al.* |
| 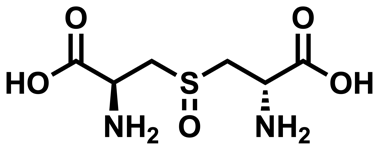 | *E. coli* | No inhibition | ’’ | Lam *et al.* |
| 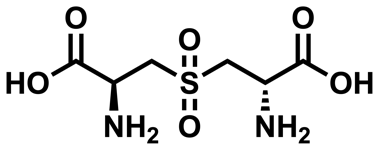 | *E. coli* | No inhibition | ’’ | Lam *et al.* |
| 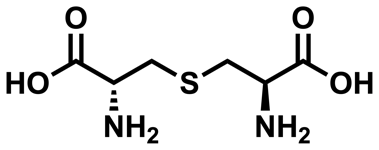 | *E. coli* | *K*_i_ 9.1 mM | ’’ | Lam *et al.* |
| 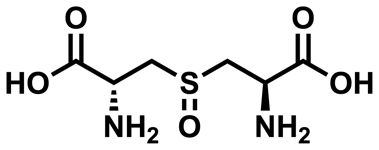 | *E. coli* | No inhibition | ’’ | Lam *et al.* |
| 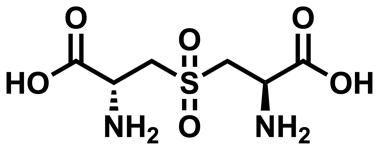 | *E. coli* | No inhibition | ’’ | Lam *et al.* |
| 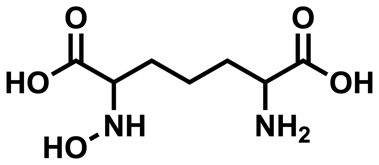 | *E. coli* | *K*_i_ 5.6 µM | ’’ | Lam *et al.* |
| 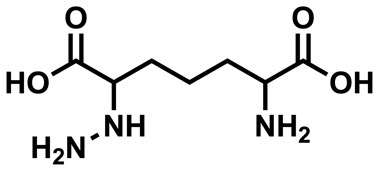 | *E. coli* | *K*_i_ 2.9 mM | ’’ | Lam *et al.* |
| 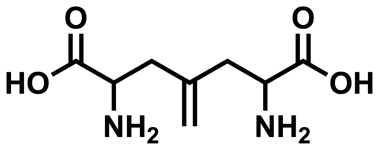 | *E. coli* | *K*_i_**^’^** 0.95 mM | ’’ | Lam *et al.* |
| 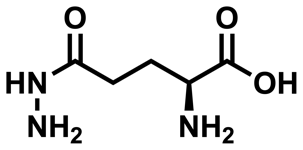 | *E. coli* | No inhibition | ’’ | Lam *et al.* |
| 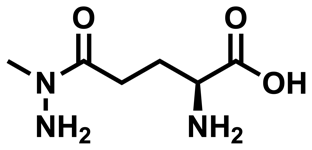 | *E. coli* | No inhibition | ’’ | Lam *et al.* |
| 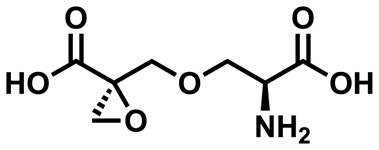 | *Anabaena sp.* YBS01 | No inhibition |  | This work |
| 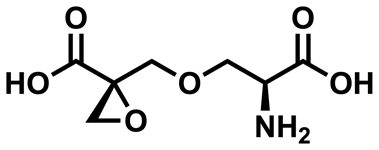 | *Anabaena sp.* YBS01 | No inhibition |  | This work |
| 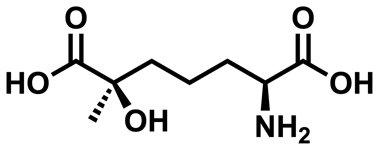 | *Anabaena sp.* YBS01 | IC_50_ > 5 mM |  | This work |
| 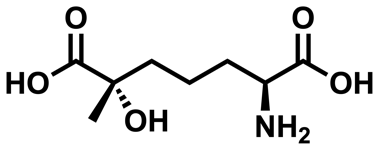 | *Anabaena sp.* YBS01 | IC_50_ > 5 mM |  | This work |
| 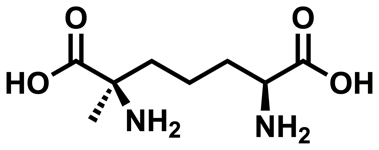 | *Anabaena sp.* YBS01 | IC_50_ > 5 mM |  | This work |
| 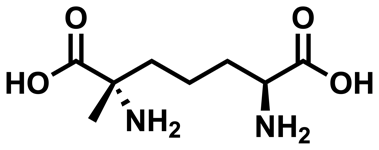 | *Anabaena sp.* YBS01 | IC_50_ > 5 mM |  | This work |

Table S2**. Crystal structures of DAP epimerases.** Rows highlighted in blue are structures with substrate/inhibitor bound in the active site.

| **Organism** | **PDB ID** | **Form** | **Resolution** | **Conformation** | **Reference** |
| --- | --- | --- | --- | --- | --- |
| *H. influenzae* | 1BWZ | Wild-type; apo | 2.72 Å | Disulfide bond C73 and C217, inactive | Cirilli, M., Zheng, R., Scapin, G., & Blanchard, J. S. (1998). Biochemistry, 37(47), 16452–16458. |
| *H. influenzae* | 1GQZ | Wild-type; apo | 1.75 Å | Disulfide bond C73 and C217, inactive | Lloyd, A. J., Huyton, T., Turkenburg, J., & Roper, D. I. (2004). Acta Crystallographica Section D: Biological Crystallography, 60(2), 397–400. |
| *H. influenzae* | 2Q9H | C73S; apo | 2.30 Å | Open, unbound | Pillai, B., Cherney, M., Diaper, C. M., Sutherland, A., Blanchard, J. S., Vederas, J. C., & James, M. N. G. (2007). Biochemical and Biophysical Research Communications, 363(3), 547–553. |
| *H. influenzae* | 2Q9J | C217S; apo | 2.20 Å | Open, unbound | Pillai *et al*. (2007). |
| *H. influenzae* | 2GKE | Wild-type; L,L-aziDAP covalently bound | 1.35 Å | Closed, inhibitor-bound (active) | Pillai, B., Cherney, M. M., Diaper, C. M., Sutherland, A., Blanchard, J. S., Vederas, J. C., & James, M. M. G. (2006). Proceedings of the National Academy of Sciences of the United States of America, 103(23), 8668–8673. |
| *H. influenzae* | 2GKJ | Wild-type; D,L-aziDAP covalently bound | 1.70 Å | Closed, inhibitor-bound (active) | Pillai *et al*. (2006). |
| *E. coli* | 4IKO | Wild-type; apo | 2.05 Å | Open, unbound | Hor, L., Dobson, R. C. J., Dogovski, C., Hutton, C. A., & Perugini, M. A. (2009). Acta Crystallographica Section F: Structural Biology and Crystallization Communications, 66(1), 37–40. |
| *E. coli* | 4IJZ | Y268A; apo | 2.00 Å | Open, unbound, monomeric (inactive) | Hor, L., Dobson, R. C. J., Downton, M. T., Wagner, J., Hutton, C. A., & Perugini, M. A. (2013). Journal of Biological Chemistry, 288(13), 9238–9248. |
| *Mycobacterium tuberculosis* | 3FVE | Wild-type; apo | 2.60 Å | Disulfide bond C87 and C226, inactive | Usha, V., Dover, L. G., Roper, D. I., Fütterer, K., & Besra, G. S. (2009). Acta Crystallographica Section D: Biological Crystallography, 65(4), 383–387. |
| *Bacillus anthracis* | 2OTN | Wild-type; apo | 2.40 Å | Open, unbound | To be published; Matho, M.H., Fukuda, K., Lloyd, A.J., Santelli, E., Jaroszewski, L., Scott, D.J., Liddington, R.C., Roper, D. |
| *A. thaliana* | 3EJX | Wild-type; L,L-aziDAP covalently bound | 1.95 Å | Closed, inhibitor-bound (active) | Pillai, B., Moorthie, V. A., van Belkum, M. J., Marcus, S. L., Cherney, M. M., Diaper, C. M., Vederas, J. C., James, M. N. G. (2009). Journal of Molecular Biology, 385(2), 580–594. |
| *A. thaliana* | 3EKM | Wild-type; D,L-aziDAP covalently bound | 2.30 Å | Closed, inhibitor-bound (active) | Pillai *et al.* (2009). |
| *C. glutamicum* | 5H2G | Wild-type; apo | 2.00 Å | Disulfide bond C87 and C226, inactive | Sagong, H. Y., & Kim, K. J. (2017). Scientific Reports, 7(1), 1–13. |
| *C. glutamicum* | 5H2Y | Wild-type; apo | 2.00 Å | Open, unbound | Sagong *et al.* |
| *C. glutamicum* | 5M47 | Wild-type; D,L-DAP non-covalently bound* | 2.59 Å | Closed, substrate-bound. *However, bound in an inactive conformation (substrate D stereocenter in distal site, L in proximal) | Sagong *et al.* |
| *Anabaena sp.* YBS01 |  | Wild-type; D,L-aziDAP covalently bound |  | Closed, inhibitor-bound (active) | This work |
| *Anabaena sp.* YBS01 |  | Wild-type; D,L-methylDAP non-covalently bound |  | Closed, inhibitor-bound (active) | This work |

Table S3**. Crystallography data collection and refinement statistics.**

|  | **DapF in complex with D,L-aziDAP** | **DapF in complex with L,L-aziDAP** | **DapF in complex with D,L-α-methylDAP** |
| --- | --- | --- | --- |
| **PDB ID** | **9MRO** | **9MRP** | **9MRV** |
| **Data collection** |  |  |  |
| Space group | P1 | P 1 2 1 | P1 |
| **Cell dimensions** |  |  |  |
| a, b, c (Å) | 47.62 103.77 103.77 | 179.46 102.60 47.67 | 47.50 103.88 103.87 |
| α, β, γ (°) | 60.23 78.53 78.54 | 90.00 103.126 90.00 | 61.01 78.43 78.62 |
| **Resolution (Å)** | 39.30 - 1.59 | 45.15 - 1.5 | 45.39 - 1.69 |
| ***R*_merge_ (last shell)** | 0.07 (1.06) | 0.09 (1.35) | 0.05 (0.63) |
| ***I* / σ*I* (last shell)** | 9.69 (0.93) | 7.53 (1.55) | 24.30 (2.68) |
| **Completeness % (last shell)** | 94.71 (83.86) | 98.32 (98.60) | 95.69 (93.12) |
| **Observations (last shell)** | 1516133 (41963) | 1817339 (184363) | 1288756 (127799) |
| **Redundancy** | 3.6 (3.5) | 6.9 (7.0) | 7.1 (7.2) |
| **Refinement** |  |  |  |
| Resolution Å | 1.61 - 1.5 | 1.55 - 1.5 | 1.75 - 1.69 |
| No. reflections (No. reflections in cross validation) | 420990 (11893) | 264860 (26460) | 181124 (17669) |
| *R*_work_ / *R*_free_ (last shell) | 0.21/0.21 (0.33/0.34) | 0.23/0.24 (0.36/0.36) | 0.20/0.20 (0.31/0.31) |
| **No. atoms (non-hydrogen atoms)** | **10132** | **9555** | **10247** |
| Protein | 8508 | 8508 | 8505 |
| Ligand/ion | 56 | 112 | 112 |
| Water | 1568 | 991 | 1683 |
| ***B*-factors** | **23.74** | **23.78** | **22.35** |
| Protein | 23.77 | 23.82 | 22.37 |
| Ligand/ion | 19.88 | 17.96 | 18.84 |
| Water | 23.74 | 23.78 | 22.35 |
| **R.m.s. deviations** |  |  |  |
| Bond lengths (Å) | 0.007 | 0.014 | 0.006 |
| Bond angles (°) | 0.88 | 1.37 | 0.96 |

**
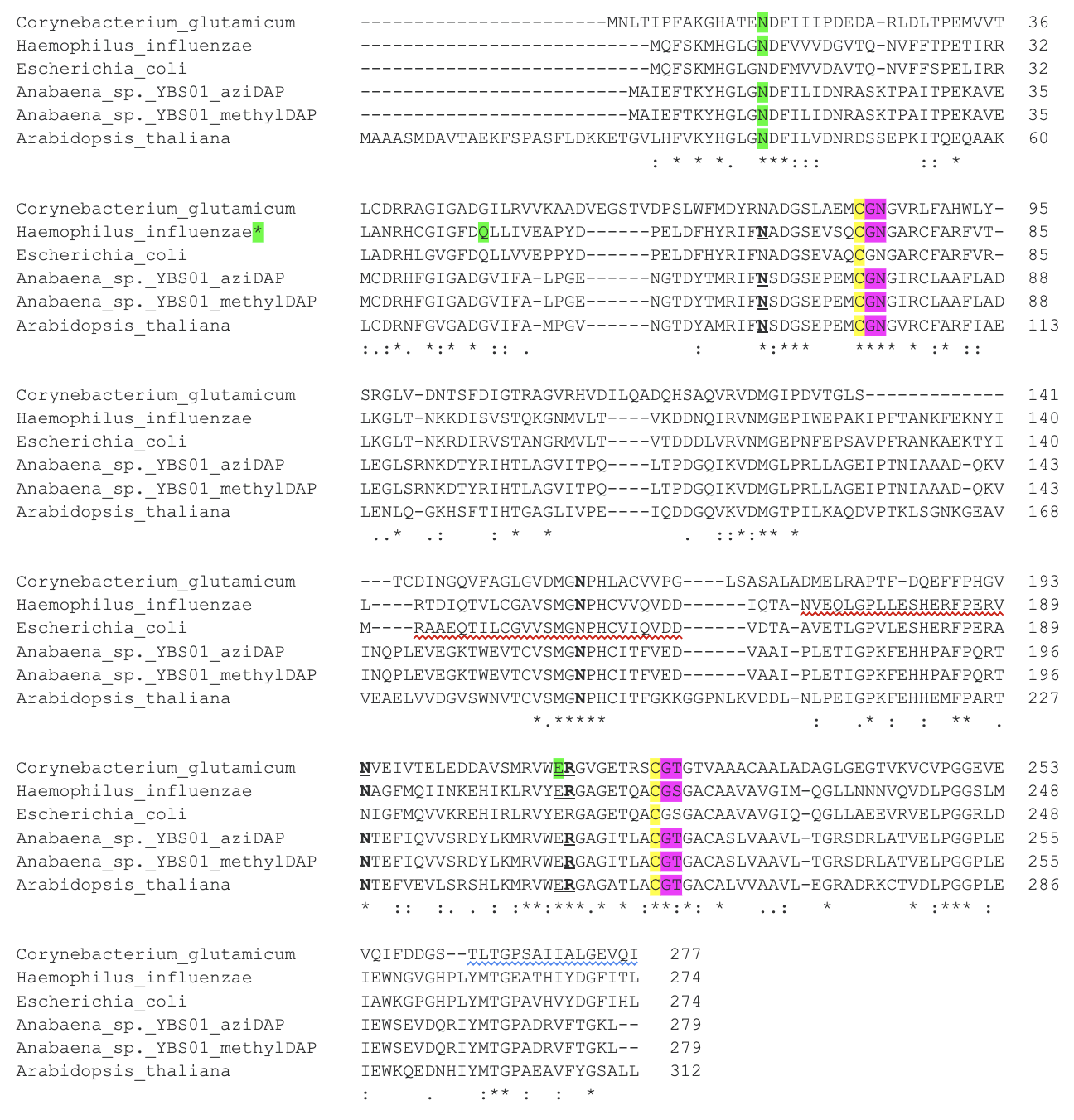
**

Figure S1**. Multiple sequence alignment of DAP epimerases.** Key residues found to form H-bonds with substrate/inhibitor in crystal structures are highlighted. Yellow = catalytic cysteine residues; green = H-bonds with proximal α-amine; pink = H-bonds with proximal α-carboxylate; underline = H-bonds with distal amine; **bold** = H-bonds with distal carboxylate. *C. glutamicum* bound to D,L-DAP: PDB 5M47; *H. influenzae* bound to D,L-aziDAP: PDB 2GKJ; *Anabaena* bound to D,L-aziDAP: PDB ; *A. thaliana* bound to D,L-aziDAP: PDB 3EKM. There is no substrate/inhibitor bound crystal structure of *E. coli* DapF, but it is included because it has been used for testing with most DAP epimerase inhibitors.

**
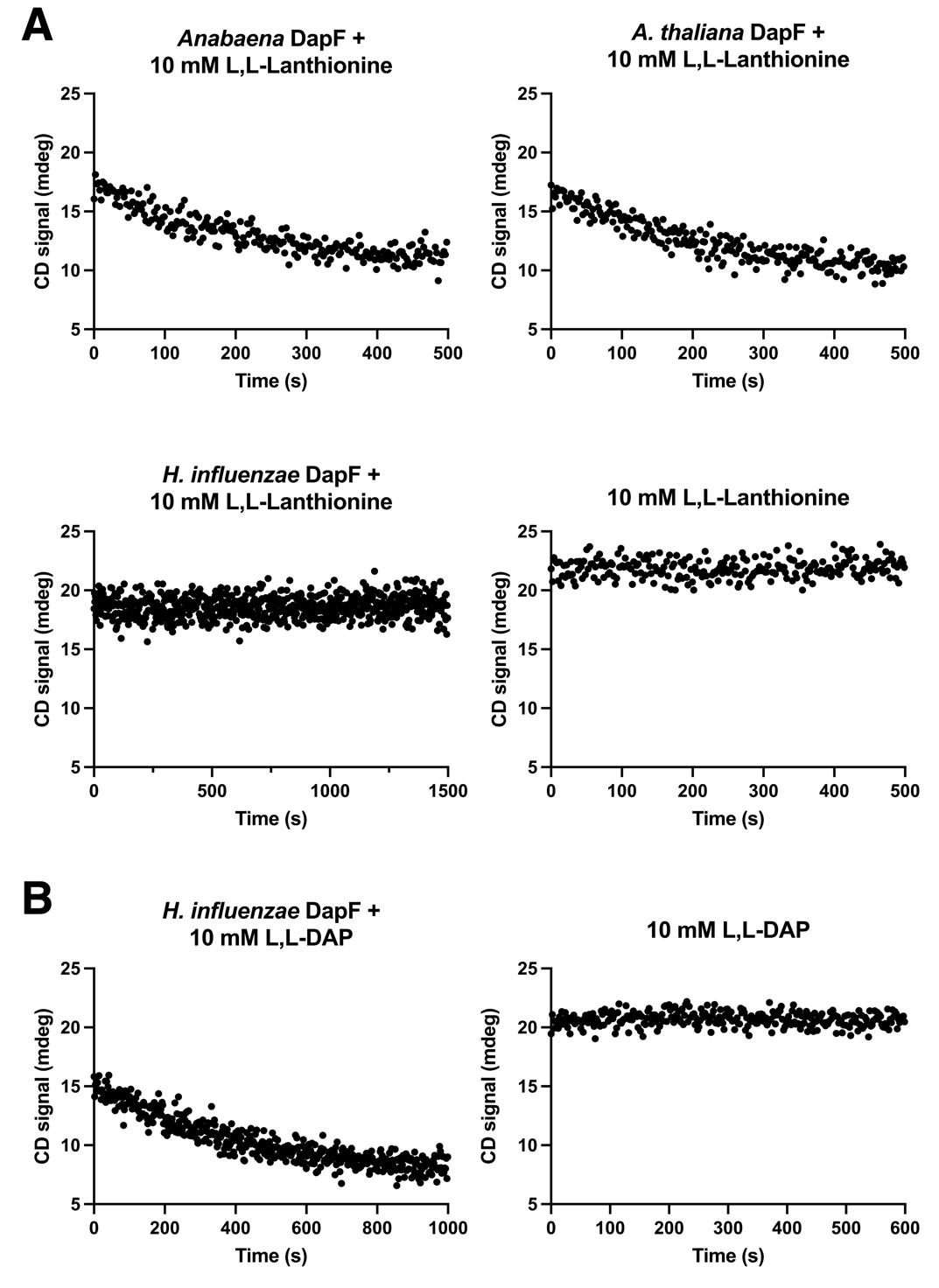
**

Figure S2**. Circular dichroism experiments to confirm that lanthionine is epimerized by DAP epimerases. A)** 10 mM solutions of L,L-lanthionine with DapF from *Anabaena*, *A. thaliana*, or *H. influenzae* added, compared to L,L-lanthionine with no epimerase added. **B)** *H. influenzae* DapF with 10 mM L,L-DAP added shows this enzyme prefers L,L-DAP over L,L-lanthionine as a substrate.

**
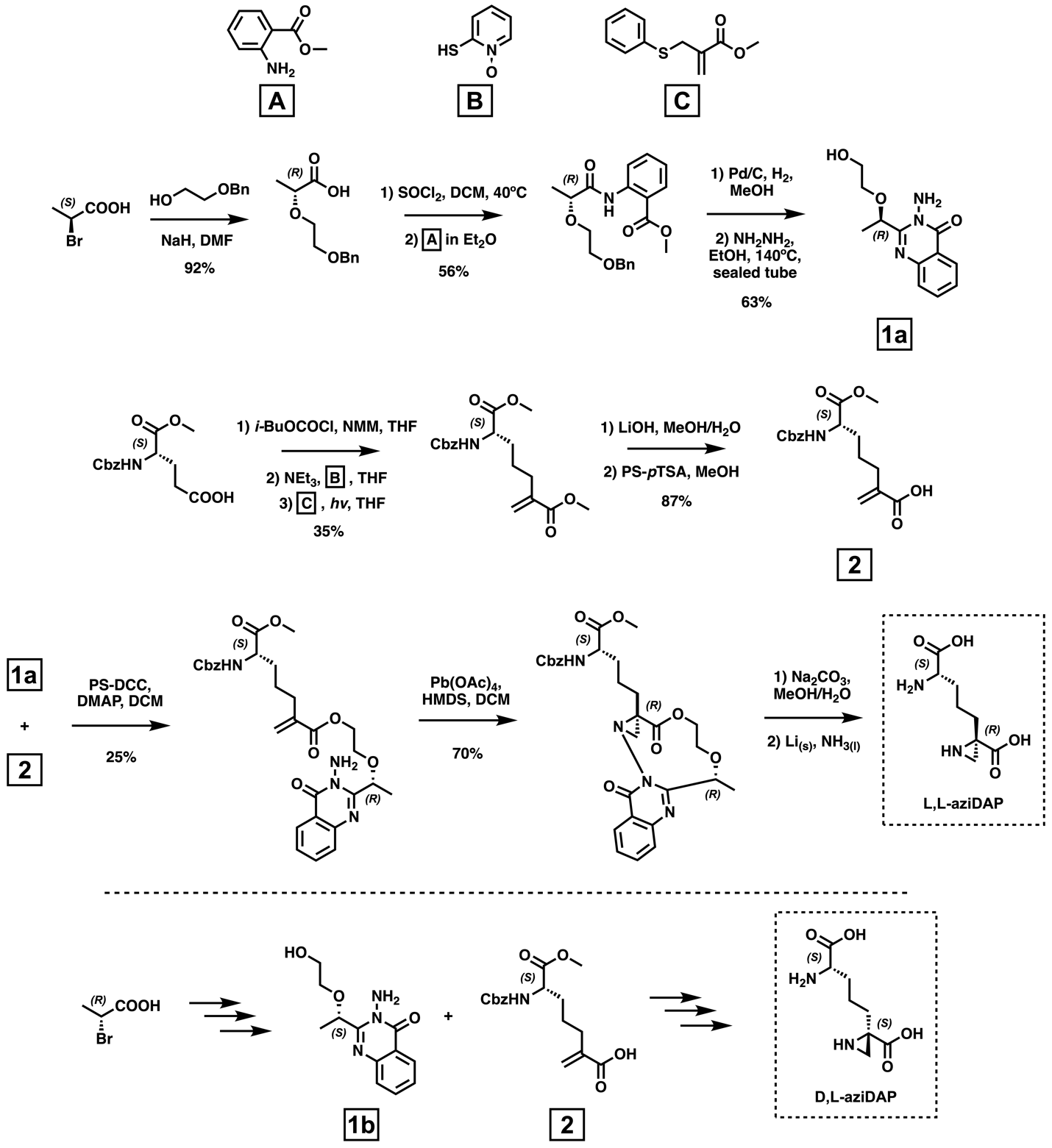
**

Figure S3**. Stereoselective synthesis of L,L-aziDAP (top) and D,L-aziDAP, as first reported by Diaper *et al.* (2005).**

**
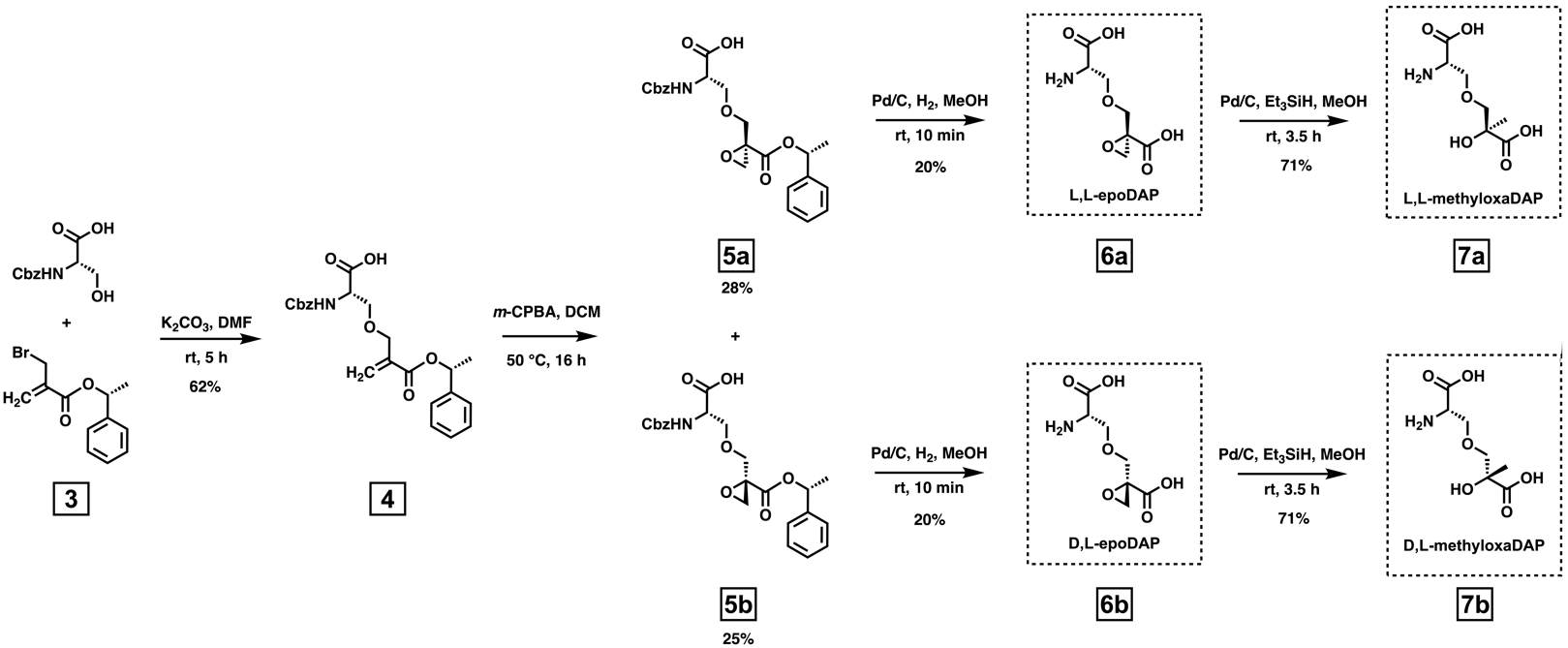
**

Figure S4**. Synthesis of pure diastereomers of D,L- and L,L-epoDAP, followed by reduction to D,L- and L,L-methyloxaDAP.**

**
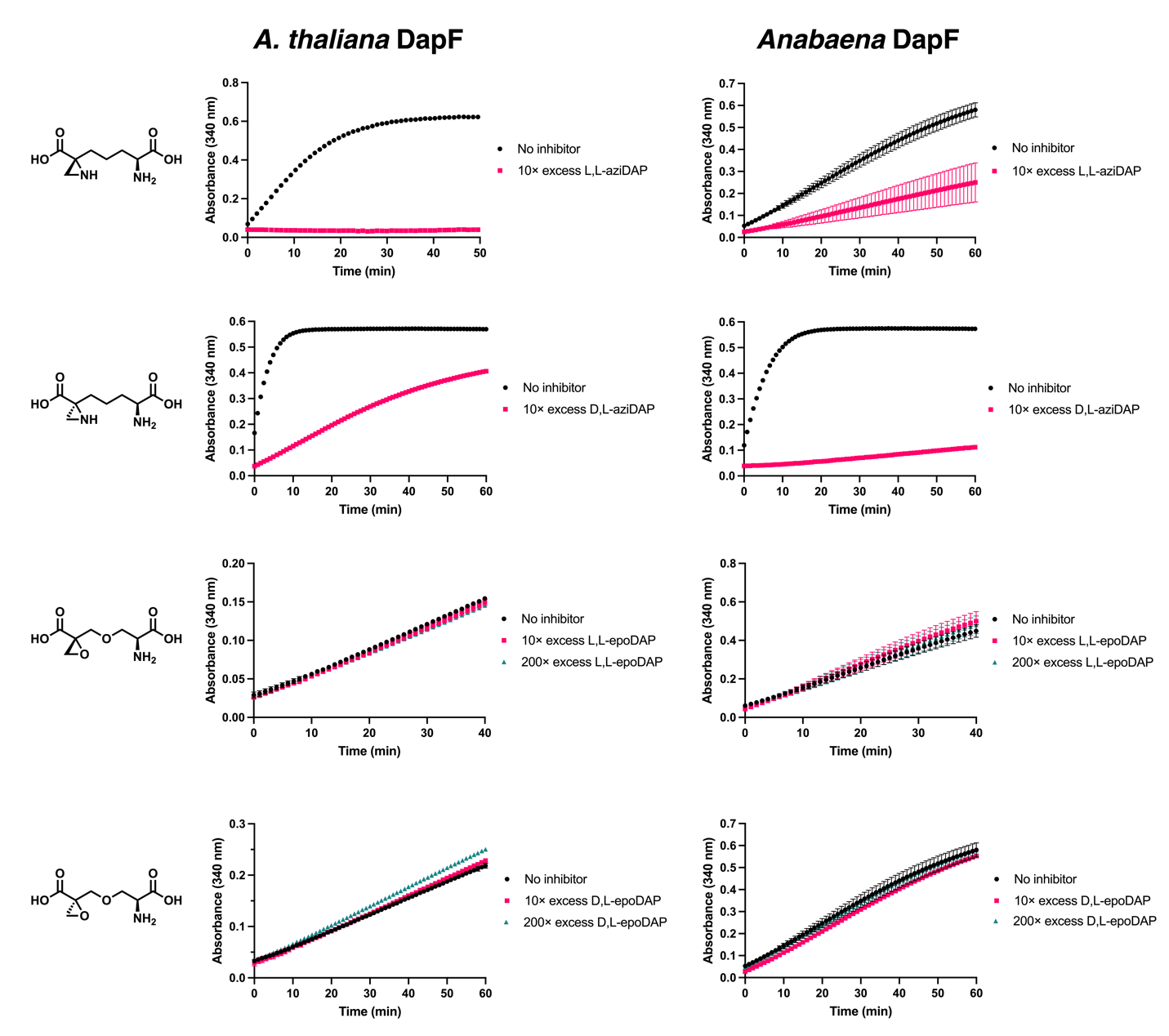
**

Figure S5**. Inhibition of *A. thaliana* and *Anabaena* DAP epimerases by covalent aziDAP and epoDAP compounds.** Enzymes were incubated with inhibitors overnight (10× or 200× molar excess of inhibitor relative to enzyme) at 4 °C before being diluted for use in a coupled enzyme assay with DAP dehydrogenase, L,L-DAP, and NADP^+^. Production of NADPH is monitored at 340 nm. The positive control was enzyme incubated overnight without inhibitor added.


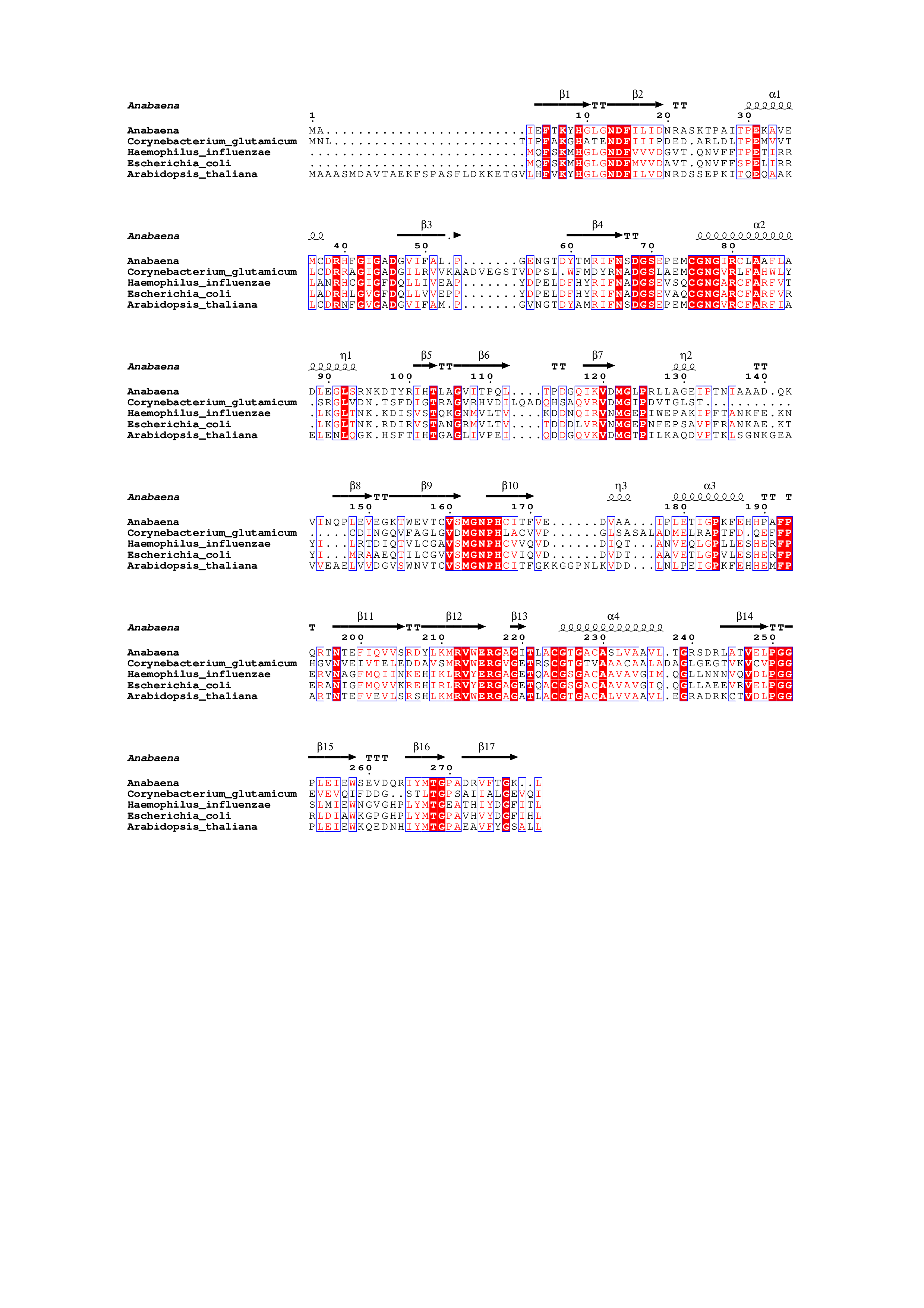
Figure S6**. Multiple sequence alignment of DAP epimerases with secondary structural elements of *Anabaena* DapF indicated.**


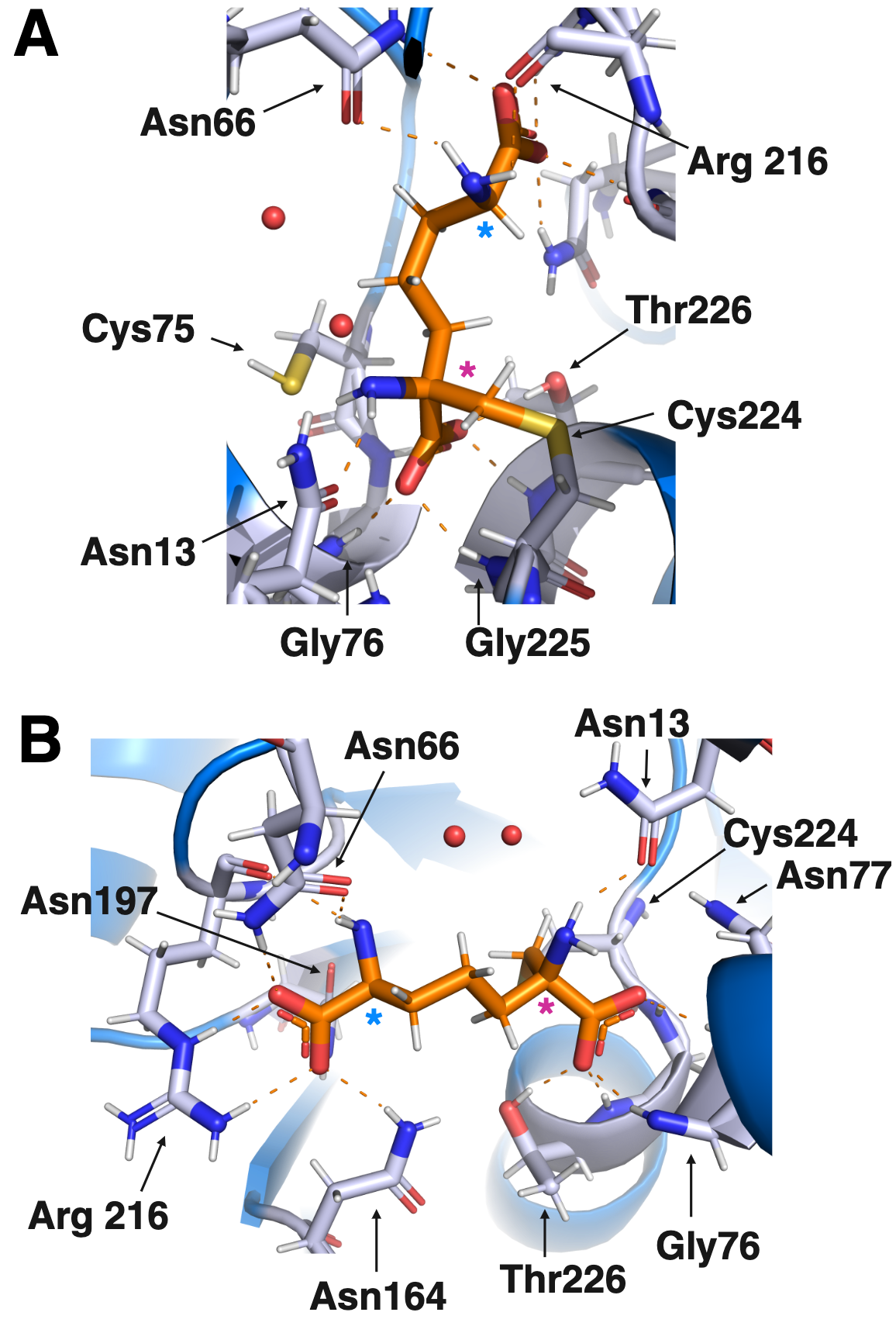


Figure S7**. *Anabaena* DapF active site residues involved in forming H-bonds with D,L-aziDAP.** A) Overall view of D,L-aziDAP bound in the enzyme active site. The proximal α-carbon (catalytic center) is labelled with a pink *, while the distal α-carbon is labelled with a blue *. B) Side view of D,L-aziDAP bound in the active site.

**
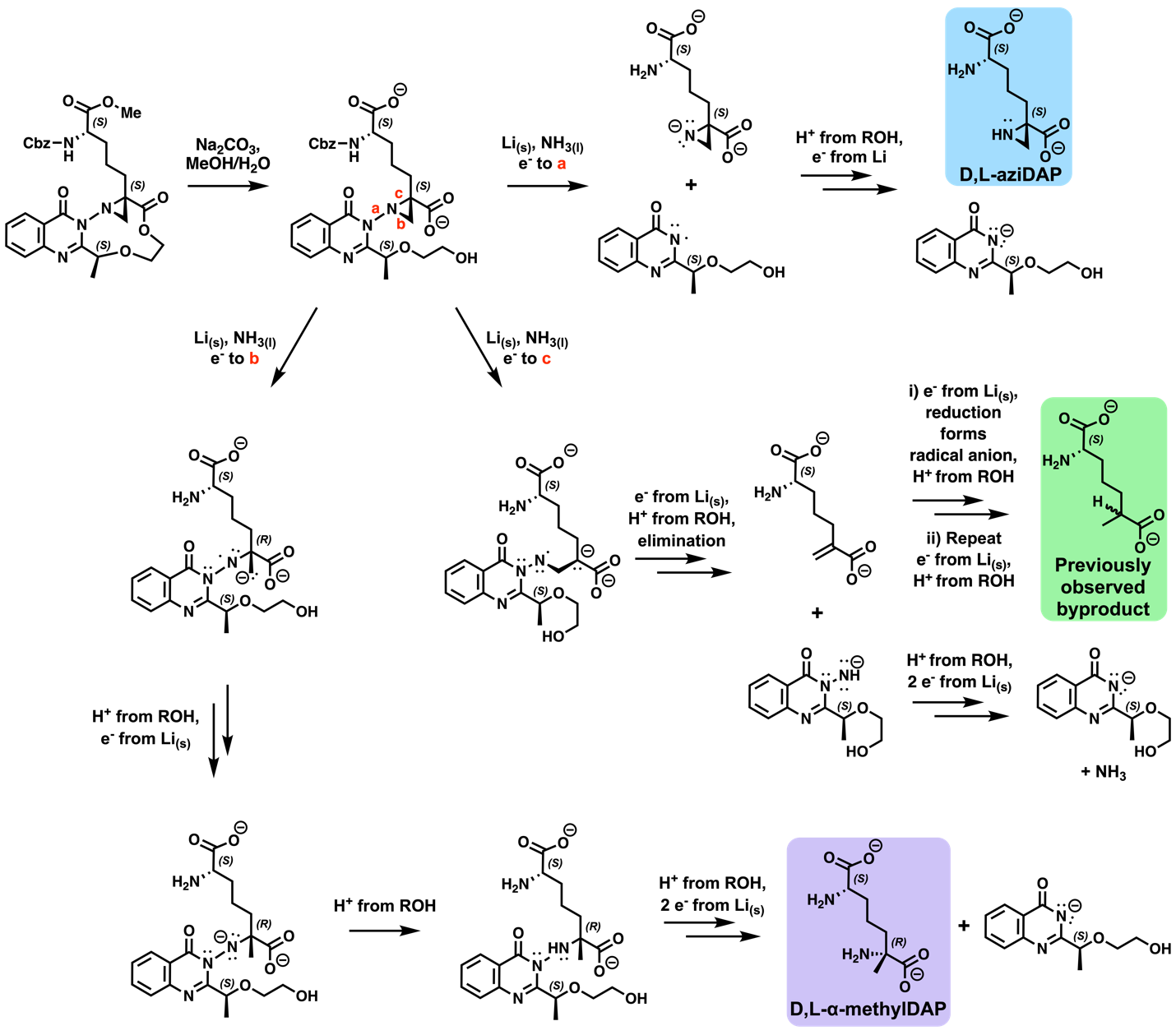
**

Figure S8**. Proposed route for formation of D,L-aziDAP, D,L-α-methylDAP, and a previously observed deaminated byproduct during Li_(s)_, NH_3(l)_ reduction of D,L-** **aziDAP precursor.**

**
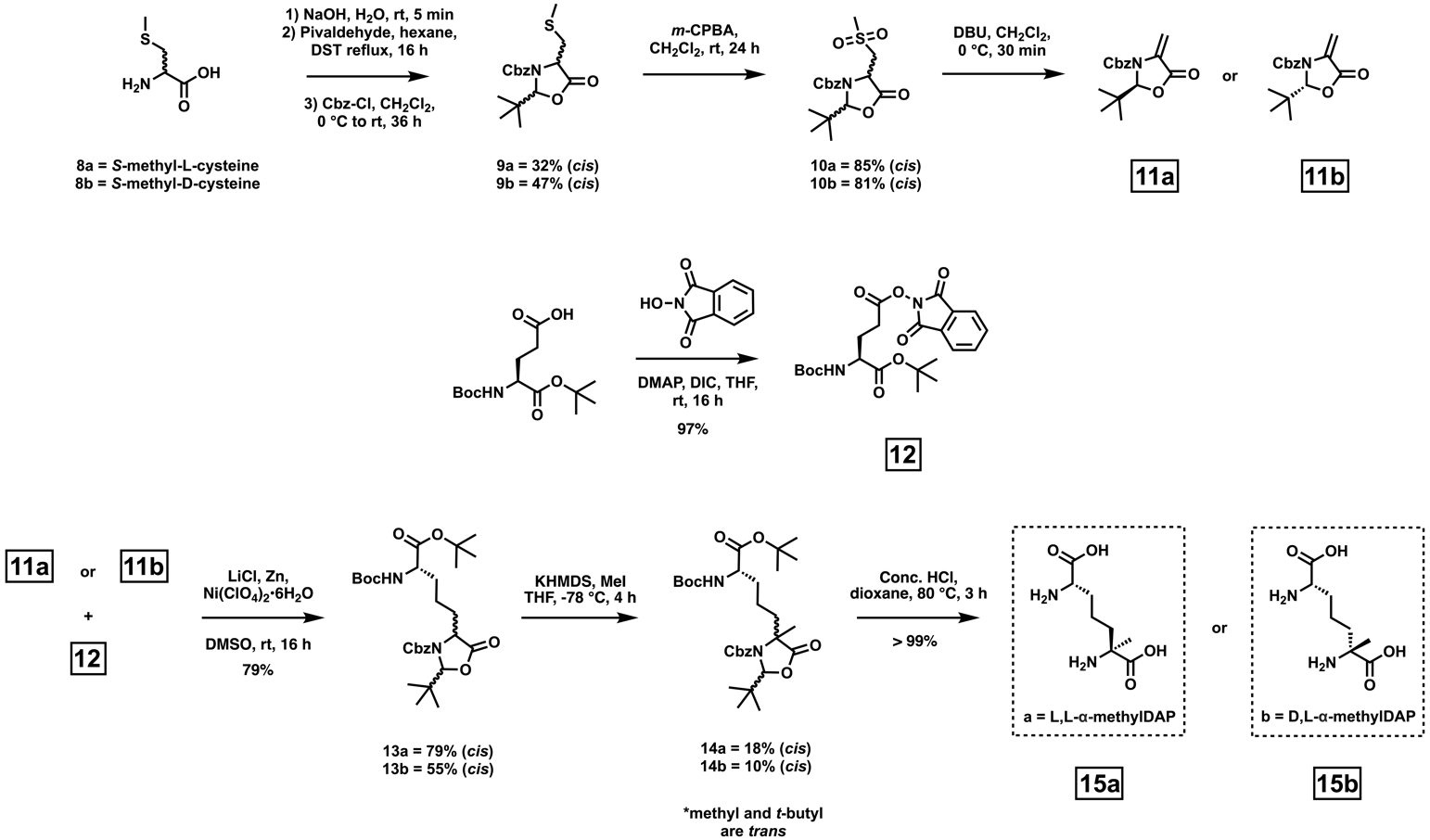
**

Figure S9**. Stereoselective synthesis of D,L-α-methylDAP and L,L-α-methylDAP.**

**
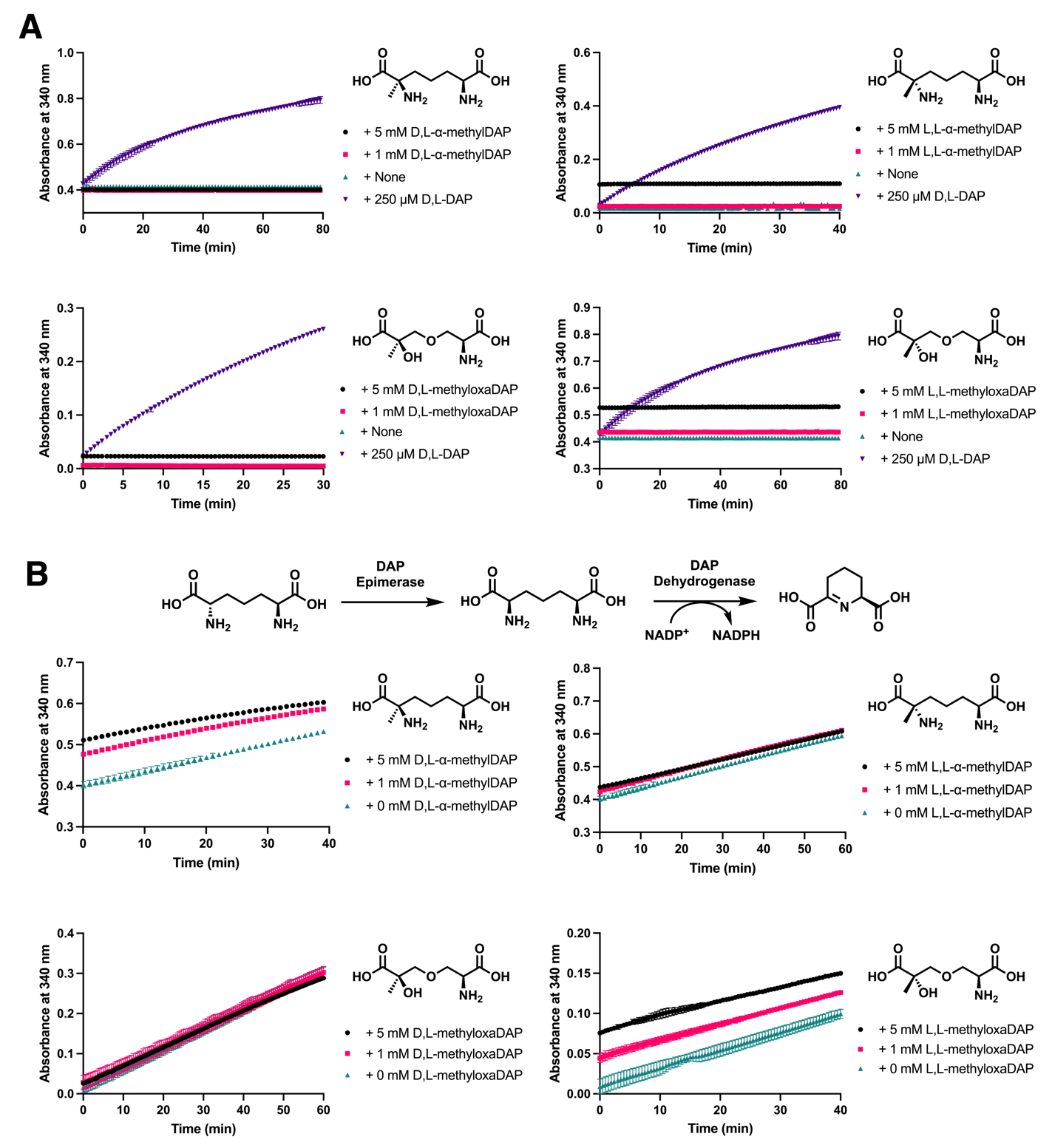
**

Figure S10**. Methylated DAP analogues are not substrates for DAP dehydrogenase, or significant inhibitors of *Anabaena* DAP epimerase (or DAP dehydrogenase). A)** Enzyme assay with DAP dehydrogenase to ensure methylated DAP analogues are not substrates for this enzyme. Experiments were performed in duplicate, and contained DAP dehydrogenase, NADP^+^, and D,L-DAP or specified compound. Absorbance at 340 nm monitors production of NADPH. **B)** Coupled enzyme assay with DapF, DAP dehydrogenase, L,L-DAP, NADP^+^, and specified compounds. Significant inhibition was not observed, even at concentrations as high as 5 mM.


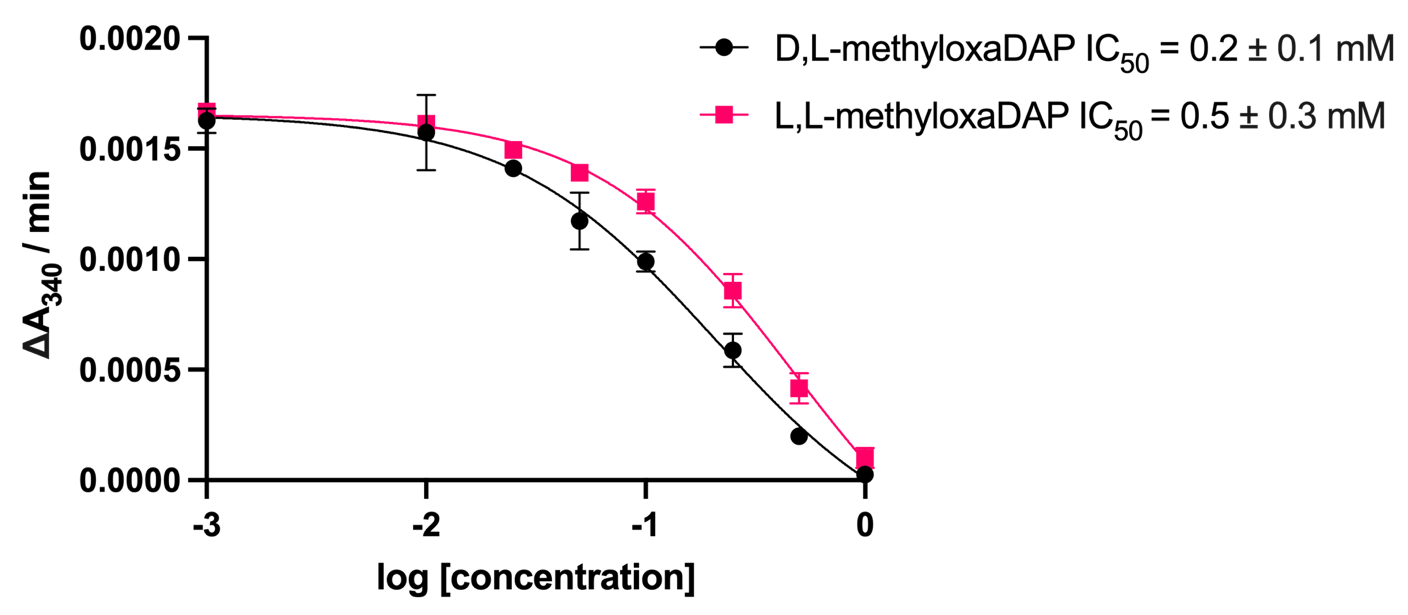


Figure S11**. IC_50_ values for D,L- and L,L-methyloxaDAP with *Anabaena* DAP epimerase after 20 h incubation.**

**
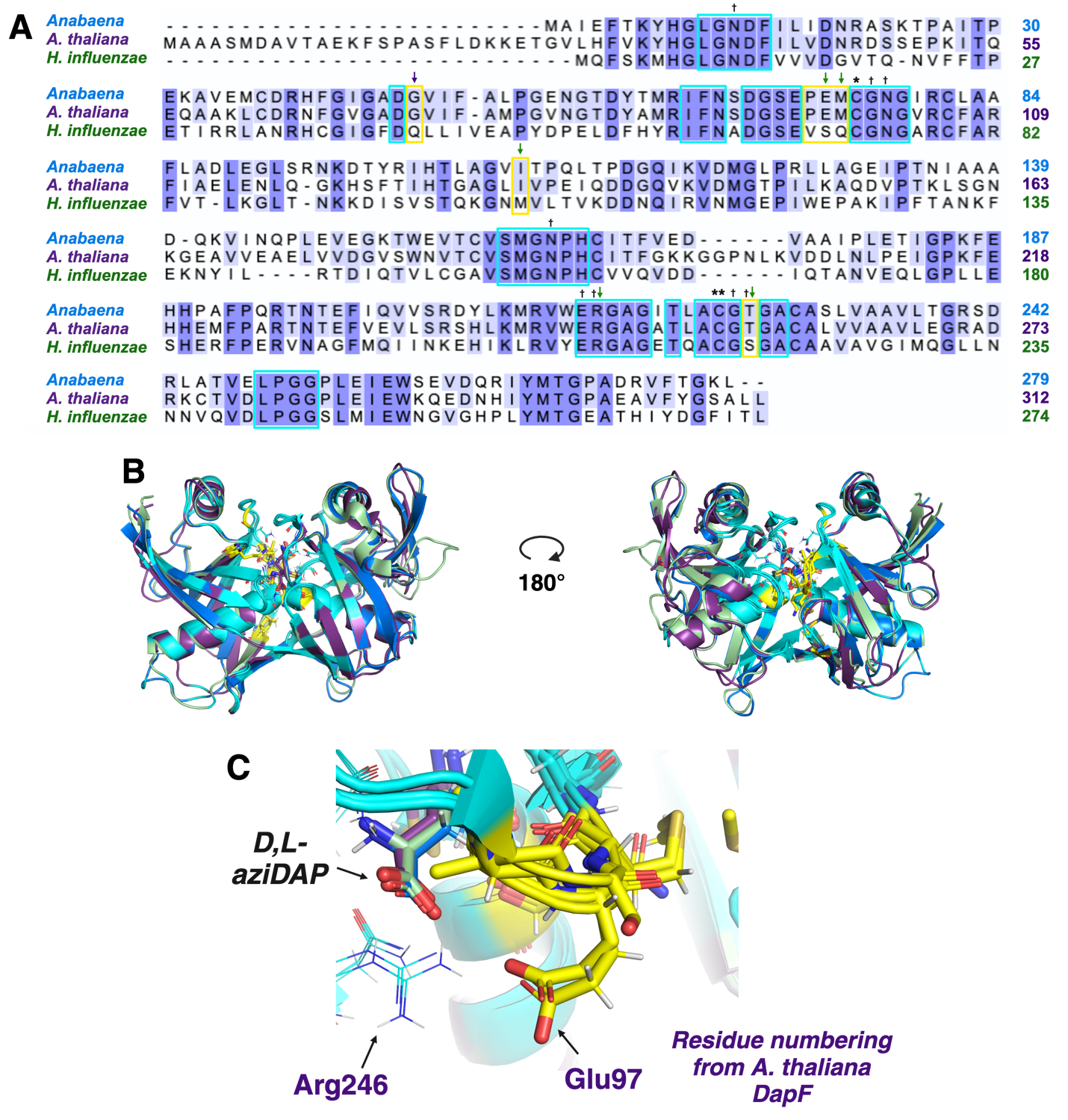
**Figure S12**. Differences and similarities in active site residues of *H. influenzae* (green), *A. thaliana* (purple), and *Anabaena* (dark blue) DAP epimerases bound to D,L- and L,L-aziDAP. A)** Multiple sequence alignment of the three enzymes. Catalytic cysteine residues are indicated with * and **, while residues involved in H-bonds with the inhibitor are indicated with ^†^. Conserved residues found in the active site pocket are boxed in cyan, while varied residues in the active site are boxed in yellow. Residues highlighted in C) and D) are indicated with appropriately coloured arrows. **B)** Structural alignment of the three enzymes, with conserved residues highlighted in cyan, and key active site residues that vary between enzymes highlighted in yellow. Cyan regions indicated conserved residues amongst all three enzymes. **C)** Structural alignment of the three enzymes with a focus on the varied loop region that forms a salt bridge between Glu97 and Arg246 in *A. thaliana* (also present in *Anabaena*, but absent from *H. influenzae*).

# Supporting information methods

# Protein and DNA sequences

| **Protein** | **Sequence** | **MW (Da)** | **Extinction coefficient (reduced thiols; M^-1^cm^-1^)** |
| --- | --- | --- | --- |
| *Anabaena sp.* YBS01 DapF | MAIEFTKYHGLGNDFILIDNRASKTPAITPEKAVEMCDRHFGIGADGVIFALPGENGTDYTMRIFNSDGSEPEMCGNGIRCLAAFLADLEGLSRNKDTYRIHTLAGVITPQLTPDGQIKVDMGLPRLLAGEIPTNIAAADQKVINQPLEVEGKTWEVTCVSMGNPHCITFVEDVAAIPLETIGPKFEHHPAFPQRTNTEFIQVVSRDYLKMRVWERGAGITLACGTGACASLVAAVLTGRSDRLATVELPGGPLEIEWSEVDQRIYMTGPADRVFTGKL**HHHHHH** | 31142 | 23950 |
| *Symbiobacterium thermophilum* IAM 14863 DAP dehydrogenase | M**HHHHHH**DKLRVAVVGYGNVGRYALEAVQAAPDMELVGVVRRKVLAATPPELTGVRVVTDISQLEGVQGALLCVPTRSVPEYAEAMLRRGIHTVDSYDIHGDLADLRRRLDPVAREHGAAAVISAGWDPGTDSIIRALLEFMAPKGITYTNFGPGMSMGHSVAVKAIPGVRDALSMTIPAGMGVHKRAVYVELEPGADFAEVERAIKTDPYFVRDETRVTQVESVSALMDVGHGVVMERKGVSGATHNQLFRFEMRINNPALTAQVMVAALRAAARQKPGCYTMIEIPVIDYLPGDREAWIRKLV | 33265 | 24535 |
| *A. thaliana* DapF (leader sequence removed; cloning previously described in Pillai *et al.*, JMB, 2009) | **M**AAASMDAVTAEKFSPASFLDKKETGVLHFVKYHGLGNDFILVDNRDSSEPKITQEQAAKLCDRNFGVGADGVIFAMPGVNGTDYAMRIFNSDGSEPEMCGNGVRCFARFIAELENLQGKHSFTIHTGAGLIVPEIQDDGQVKVDMGTPILKAQDVPTKLSGNKGEAVVEAELVVDGVSWNVTCVSMGNPHCITFGKKGGPNLKVDDLNLPEIGPKFEHHEMFPARTNTEFVEVLSRSHLKMRVWERGAGATLACGTGACALVVAAVLEGRADRKCTVDLPGGPLEIEWKQEDNHIYMTGPAEAVFYGSALL**HHHHHH** | 34394 | 22460 |
| *H. influenzae* ATCC 51907 DapF | MQFSKMHGLGNDFVVVDGVTQNVFFTPETIRRLANRHCGIGFDQLLIVEAPYDPELDFHYRIFNADGSEVSQCGNGARCFARFVTLKGLTNKKDISVSTQKGNMVLTVKDDNQIRVNMGEPIWEPAKIPFTANKFEKNYILRTDIQTVLCGAVSMGNPHCVVQVDDIQTANVEQLGPLLESHERFPERVNAGFMQIINKEHIKLRVYERGAGETQACGSGACAAVAVGIMQGLLNNNVQVDLPGGSLMIEWNGVGHPLYMTGEATHIYDGFITL**HHHHHH** | 31071 | 19940 |

***Anabaena sp.* YBS01 DapF codon optimized DNA sequence inserted into pET-24b(+) at 5’ *Nde*I and 3’ *Hind*III restriction sites:**

CAT**ATGGCGATTGAGTTTACGAAGTACCATGGGCTGGGAAACGACTTTATTCTTATAGATAACCGCGCATCCAAAACTCCAGCCATAACTCCGGAGAAAGCGGTCGAAATGTGTGACCGTCACTTTGGGATCGGCGCTGATGGCGTAATTTTCGCGCTGCCGGGAGAGAACGGGACTGATTACACAATGCGTATTTTTAACAGCGATGGTTCGGAGCCGGAGATGTGCGGAAACGGCATCCGCTGTCTTGCGGCATTCTTAGCCGACTTGGAGGGCCTTTCACGCAACAAAGACACATATCGTATTCATACGCTGGCTGGAGTGATAACGCCTCAGCTTACCCCCGACGGACAGATTAAGGTAGATATGGGCTTACCTCGTCTTTTAGCTGGAGAGATTCCGACTAATATAGCTGCCGCAGATCAGAAGGTAATCAATCAACCACTTGAGGTAGAGGGCAAGACATGGGAGGTCACATGCGTATCGATGGGGAATCCTCACTGCATCACTTTCGTAGAGGATGTCGCGGCTATCCCGCTGGAGACAATAGGACCGAAGTTTGAGCACCACCCGGCCTTCCCACAGCGTACCAACACAGAGTTTATTCAGGTTGTATCGCGGGATTATTTGAAAATGCGGGTTTGGGAACGTGGCGCTGGCATTACATTGGCTTGCGGCACAGGCGCGTGCGCGAGCCTGGTTGCGGCCGTTTTGACTGGTCGCTCGGATAGACTTGCGACTGTGGAGTTGCCCGGAGGGCCGTTGGAAATCGAATGGAGTGAAGTGGACCAACGTATATATATGACTGGACCTGCCGACCGGGTTTTTACGGGTAAATTGCACCATCACCACCACCACTAA**GCTT

***Symbiobacterium thermophilum* IAM 14863 DAP dehydrogenase codon optimized DNA sequence inserted into pET-24b(+) at 5’ *Nde*I and 3’ *Hind*III restriction sites:**

CAT**ATGCACCACCACCACCATCATGATAAATTACGGGTGGCAGTAGTCGGCTATGGGAATGTGGGTCGGTATGCCCTTGAGGCAGTTCAAGCAGCGCCGGACATGGAGCTGGTTGGGGTAGTGCGTCGTAAGGTTTTGGCCGCTACTCCGCCGGAGCTTACTGGAGTTCGGGTAGTTACAGATATTAGCCAGCTGGAGGGAGTCCAGGGTGCCTTACTTTGCGTACCGACGCGTAGCGTCCCGGAGTATGCTGAAGCGATGTTACGCAGAGGTATCCACACTGTCGATAGCTACGATATTCATGGGGACCTTGCTGACCTGCGGAGACGCCTGGATCCAGTGGCACGCGAGCATGGGGCCGCTGCAGTGATCAGCGCAGGATGGGATCCAGGTACCGACTCCATCATTCGTGCTCTGCTTGAGTTTATGGCTCCAAAGGGAATCACATATACAAACTTCGGTCCAGGGATGAGCATGGGACATTCTGTGGCAGTTAAAGCTATACCCGGTGTTAGAGATGCATTGTCAATGACAATACCAGCCGGGATGGGTGTTCATAAACGGGCCGTCTACGTTGAATTAGAACCTGGGGCCGACTTTGCTGAGGTAGAACGTGCCATAAAGACCGATCCTTATTTTGTTCGCGATGAGACAAGAGTAACGCAGGTCGAATCAGTTAGTGCGTTGATGGACGTTGGACATGGAGTGGTTATGGAGAGAAAGGGCGTCTCTGGTGCCACGCATAATCAGCTTTTCCGGTTCGAGATGCGTATCAATAACCCCGCATTGACAGCCCAGGTCATGGTGGCTGCACTTCGCGCAGCCGCCCGCCAGAAACCGGGCTGTTACACGATGATTGAAATCCCCGTTATTGACTATCTGCCCGGCGACCGCGAGGCGTGGATACGTAAATTGGTATAA**GCTT

***H. influenzae* DapF codon optimized DNA sequence inserted into pET-24b(+) at 5’ *Nde*I and 3’ *Hind*III restriction sites:**

CAT**ATGCAATTCTCCAAGATGCATGGCCTTGGTAACGACTTCGTAGTTGTCGACGGCGTGACACAAAACGTCTTCTTCACACCAGAAACCATCCGGCGGCTGGCAAATCGTCATTGTGGCATCGGTTTCGACCAGTTACTTATCGTAGAGGCCCCGTATGATCCAGAATTGGACTTCCACTATCGTATTTTTAACGCCGATGGATCGGAAGTTTCTCAATGCGGGAATGGGGCACGGTGTTTTGCGCGGTTCGTAACACTTAAAGGACTGACAAATAAAAAAGATATTTCGGTCAGTACGCAGAAGGGGAATATGGTACTTACCGTCAAAGATGACAATCAAATCCGGGTCAATATGGGAGAGCCCATTTGGGAACCCGCTAAAATACCATTTACCGCGAACAAATTCGAGAAAAATTACATATTACGGACAGACATCCAAACGGTATTATGCGGAGCCGTATCTATGGGAAACCCTCATTGTGTGGTGCAAGTCGACGATATACAGACGGCGAATGTAGAACAACTGGGCCCATTGCTTGAATCGCATGAAAGATTCCCCGAGCGCGTCAACGCAGGGTTCATGCAAATAATAAACAAAGAACACATCAAACTTCGCGTCTACGAACGTGGTGCAGGTGAGACCCAGGCATGTGGTAGCGGGGCCTGTGCTGCAGTCGCCGTTGGGATTATGCAAGGGTTGCTGAATAACAATGTACAGGTTGATTTACCTGGAGGGTCGCTGATGATCGAGTGGAACGGAGTTGGGCATCCATTGTACATGACTGGTGAAGCTACACATATTTACGACGGATTCATTACTCTGCACCATCATCATCATCACTAA**GCTT

# Synthetic chemistry methods

D,L- and L,L-aziDAP were synthesized as described in Diaper *et al.*, Org. Biomol. Chem., 3(24), 4402–4411, and the scheme is summarized in Figure S3.

Synthesis of D,L-epoDAP and L,L-epoDAP **(Figure S4)**

**(*R*)-1-Phenylethyl 2-(bromomethyl)acrylate (3)**

**
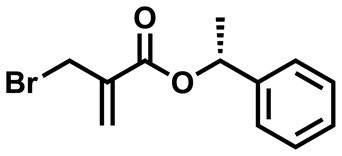
**

To a solution of bromomethylacrylic acid (1.50 g, 9.09 mmol, 1.00 equiv) and diisopropyl azodicarboxylate (1.80 mL, 9.14 mmol, 1.01 equiv) in diethyl ether (15 mL) was added a solution of (*R*)-1-phenylethanol (1.20 mL, 9.90 mmol, 1.10 equiv), followed by the addition of triphenylphosphine (2.36 g, 9.09 mmol, 1.00 equiv) in diethyl ether (15 mL) dropwise at 0 °C. The mixture was allowed to stir at 0 °C for 30 minutes then warmed to room temperature for 48 hours. The mixture was filtered and washed with Et_2_O (20 mL). After the reaction time, the solution was concentrated *in vacuo* and purified *via* flash column chromatography (5% EtOAc/hexanes) to give the pure product **3** (1.47 g, 60%) as a colorless oil; R*_f_* = 0.25 (5% EtOAc/hexanes). ^1^H NMR (500 MHz, CDCl_3_) δ 7.40 – 7.30 (5 H, m), 6.39 (1 H, d, *J* = 1.0 Hz), 6.00 (1 H, q, *J* = 6.5 Hz), 5.96 (1 H, d, *J* = 1.0 Hz), 4.21 (1 H, dd, *J* = 10.5, 1.0 Hz), 4.18 (1 H, dd, *J* = 10.5, 1.0 Hz), 1.61 (3 H, d, *J* = 6.5 Hz). ^13^C NMR (125 MHz, CDCl_3_) δ 164.1, 141.3, 137.6, 129.2, 128.6, 128.0, 126.1, 73.5, 28.3, 22.3. IR (cast film, 𝜈_max_ / cm^–1^) 3093, 3063, 2982, 2922, 2874, 1774, 1723, 1631, 1493, 1452, 1398, 1304, 1223, 1186. OR: [𝛼]_D_^26^ = –36.90 (c = 0.71, DCM). HRMS (ESI–TOF) [M+Na]^+^ calc’d for C_12_H_13_BrNaO_2_ 290.9991, found 290.9990.

**N-((Benzyloxy)carbonyl)-O-(2-(((R)-1-phenylethoxy)carbonyl)allyl)-L-serine (4)**


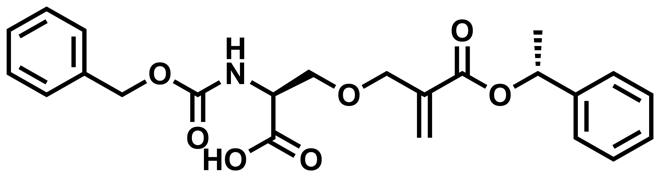


Cbz-L-Ser-OH (6.53 g, 27.2 mmol, 3.0 equiv) and acrylate **3** (2.45 g, 9.1 mmol, 1.0 equiv) were dissolved in DMF (50 mL), followed by addition of K_2_CO_3_ (8.30 g, 60.1 mmol, 6.6 equiv). The reaction was allowed to stir at room temperature for 5 hours. After that time, the reaction crude was quenched with H_2_O (100 mL) and extracted with EtOAc (3 × 50 mL). The organic layer was washed with brine (3 × 50 mL). The organic solution was concentrated *in vacuo* and purified *via* flash column chromatography (30% EtOAc/hexanes) to give the pure product **4** (2.40 g, 62%) as a colorless oil; R*_f_* = 0.15 (30% EtOAc/hexanes). ^1^H NMR (700 MHz, CDCl_3_) δ 7.36 – 7.29 (10 H, m), 6.42 (1 H, s), 5.95 (1 H, q, *J* = 7.0 Hz), 5.90 (1 H, s), 5.75 (1 H, br s), 5.12 (2 H, s), 5.02 (1 H, d, *J =* 18.9 Hz), 4.80 (1 H, d, *J* = 18.9 Hz), 4.51 – 4.49 (1 H, m), 3.88 (1 H, dd, *J* = 11.2, 2.8 Hz), 3.80 (1 H, dd, *J* = 11.2, 2.8 Hz), 1.59 (3 H, d, *J* = 7.0 Hz). ^13^C NMR (175 MHz, CDCl_3_) δ 170.0, 164.5, 156.2, 141.2, 136.1, 134.8, 128.7, 128.6, 128.5, 128.2, 128.1, 126.0, 73.5, 67.1, 63.8, 63.1, 56.2, 22.2. IR (cast film, 𝜈_max_ / cm^–1^) 3422, 3115, 3089, 3064, 3034, 2982, 2940, 2891, 1720, 1524, 1407, 1340, 1269, 1192. OR: [𝛼]_D_^26^ = –13.56 (c = 3.10, DCM). HRMS (ESI–TOF) [M–H]^–^ calc’d for C_23_H_24_NO_7_ 426.1558, found 426.1553.

***N*-((Benzyloxy)carbonyl)-*O*-(((*S*)-2-(((*R*)-1-phenylethoxy)carbonyl)oxiran-2-yl)methyl)-*L*-serine (5a) / *N*-((benzyloxy)carbonyl)-*O*-(((*R*)-2-(((*R*)-1-phenylethoxy)carbonyl)oxiran-2-yl)methyl)-*L*-serine (5b)**


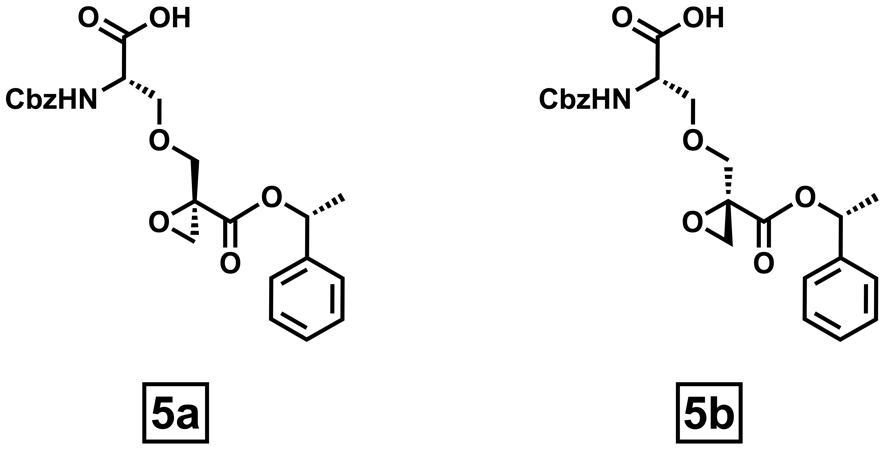


Compound **4** (372 mg, 0.87 mmol, 1 equiv) was dissolved in dichloromethane (10 mL), followed by addition of *m*CPBA (50–60%) (450 mg, 1.31 mmol, 1.5 equiv). The reaction was heated to 50 °C and allowed to stir for 16 hours. After that time, the reaction was quenched with sat. Na_2_CO_3(aq)_ (10 mL) for 30 minutes and extracted with EtOAc (3 × 10 mL). The organic solution was concentrated *in vacuo* and purified *via* flash column chromatography (5% acetone/DCM) to give the pure products **5a** (108 mg, 28%) and **5b** (96 mg, 25%), each as a colorless oil; **5a** R*_f_* = 0.45 (5% acetone/DCM) stained with Hanessian’s stain; **5b** R*_f_* = 0.40 (5% acetone/DCM) stained with Hanessian’s stain.

**5a:** ^1^H NMR (500 MHz, CDCl_3_) δ 7.40 – 7.28 (10 H, m), 5.95 (1 H, q, *J* = 6.5 Hz), 5.71 (1 H, d, *J =* 7.5 Hz), 5.14 (2 H, s), 5.04 (1 H, d, *J* = 12.5 Hz), 4.44 – 4.42 (1 H, m), 4.19 (1 H, d, *J* = 12.5 Hz), 3.81 – 3.74 (2 H, m), 3.20 (1 H, d, *J* = 6.0 Hz), 2.99 (1 H, d, *J* = 6.0 Hz), 1.60 (3 H, d, *J* = 6.5 Hz). ^13^C NMR (125 MHz, CDCl_3_) δ 169.9, 168.6, 156.1, 140.8, 136.1, 128.7, 128.6, 128.4, 128.2, 128.1, 126.0, 74.8, 67.2, 63.5, 63.2, 56.2, 54.6, 49.9, 22.1. IR (cast film, 𝜈_max_ / cm^–1^) 3376, 3091, 3066, 3034, 2982, 2942, 2889, 1728, 1520, 1455, 1343, 1267, 1179, 1062. OR: [𝛼]_D_^26^ = –37.95 (c = 0.38, DCM). HRMS (ESI–TOF) [M–H]^–^ calc’d for C_23_H_24_NO_8_ 442.1507, found 442.1508.

**5b:** ^1^H NMR (500 MHz, CDCl_3_) δ 7.40 – 7.29 (10 H, m), 5.98 (1 H, q, *J* = 6.5 Hz), 5.65 (1 H, d, *J =* 7.0 Hz), 5.15 (2 H, s), 4.70 (1 H, d, *J* = 12.5 Hz), 4.54 (1 H, d, *J* = 12.0 Hz), 4.41–4.39 (1 H, m), 3.94 (1 H, dd, *J* = 11.5, 3.0 Hz), 3.85 (1 H, dd, *J* = 11.5, 2.5 Hz), 3.15 (1 H, d, *J* = 5.5 Hz), 2.98 (1 H, d, *J* = 6.0 Hz), 1.61 (3 H, d, *J* = 6.5 Hz). ^13^C NMR (125 MHz, CDCl_3_) δ 169.8, 167.5, 156.1, 140.4, 136.1, 128.7, 128.6, 128.5, 128.3, 128.2, 126.2, 74.7, 67.2, 63.5, 63.2, 56.1, 54.5, 50.2, 21.9. IR (cast film, 𝜈_max_ / cm^–1^) 3391, 3066, 3034, 2981, 2957, 2894, 1728, 1520, 1455, 1344, 1299, 1243, 1061. OR: [𝛼]_D_^26^ = –37.59 (c = 0.340, DCM). HRMS (ESI–TOF) [M–H]^–^ calc’d for C_23_H_24_NO_8_ 442.1507, found 442.1508.

**(*S*)-2-(((*S*)-2-Amino-2-carboxyethoxy)methyl)oxirane-2-carboxylic acid (6a) / (*R*)-2-(((*S*)-2-amino-2-carboxyethoxy)methyl)oxirane-2-carboxylic acid (6b)**

**
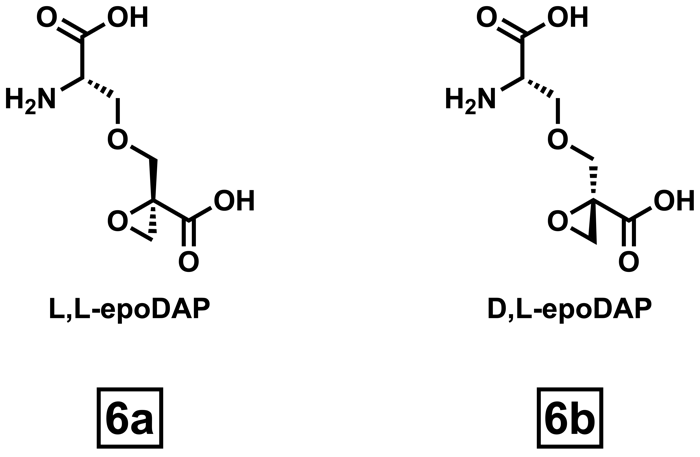
**

Compound **5a** or **5b** (20 mg, 0.045 mmol, 1.0 equiv) was dissolved in MeOH (1.0 mL) in a test tube and placed in a Parr hydrogenation reactor. 10% Pd/C (7.2 mg, 6.75 µmol, 0.15 equiv) was added to the reaction mixture. Parr hydrogenation was set to 26 psi and the reactor was shaken for 10 minutes. After that time, the reaction crude was filtered with celite and washed with MeOH (10 mL). The filtrate was concentrated *in vacuo* and purified *via* RP-HPLC using a preparative C18 column. Purification was conducted with water (solvent A) and acetonitrile (solvent B) as eluents. The gradient began with 20% MeCN/80% H_2_O for the first 2 mins, and then was ramped up to 35% MeCN over 6 mins, following by the final ramp up to 100% MeCN over the next 1 min and held for 5 min. Fractions containing the peptide eluted at 3.00 minute. Fractions were pooled and lyophilized to give the pure product **6a** or **6b** (1.80 mg, 20%) as a colorless oils.

**6a:** ^1^H NMR (500 MHz, D_2_O) δ 5.07 (1 H, d, *J* = 12.0 Hz), 4.34 – 4.31 (1 H, m), 4.15 (1 H, d, *J* = 12.5 Hz), 4.10 (1 H, dd, *J* = 13.0, 4.0 Hz), 4.00 (1 H, dd, *J* = 12.5, 3.5 Hz), 3.03 (1 H, dd, *J* = 5.0, 1.0 Hz), 3.00 (1 H, dd, *J* = 5.0, 1.0 Hz). ^13^C NMR (125 MHz, D_2_O) δ 176.8, 170.3, 70.1, 61.9, 59.6, 57.3, 52.1. IR (𝜈_max_ / cm^–1^) 3353, 3008, 2976, 2925, 2821, 1749, 1585, 1420, 1399, 1216. OR: [𝛼]_D_^26^ = –48.40 (c = 0.05, H_2_O). HRMS (ESI–TOF) [M–H]^–^ calc’d for C_7_H_10_NO_6_ 204.0514, found 204.0515.

**6b:** ^1^H NMR (700 MHz, D_2_O) δ 5.07 (1 H, d, *J* = 12.6 Hz), 4.31–4.29 (1 H, m), 4.15 (1 H, d, *J* = 12.6 Hz), 4.11 (1 H, dd, *J* = 12.6, 4.2 Hz), 4.00 (1 H, dd, *J* = 12.6, 4.2 Hz), 3.03 (1 H, d, *J* = 4.9 Hz), 3.00 (1 H, d, *J* = 4.9 Hz). ^13^C NMR (175 MHz, D_2_O) δ 176.8, 170.3, 68.4, 60.4, 58.1, 55.7, 50.6. IR (𝜈_max_ / cm^–1^) 3469, 3347, 3273, 3048, 2972, 2840, 1742, 1575, 1515, 1420, 1236. OR: [𝛼]_D_^26^ = +66.00 (c = 0.05, H_2_O). HRMS (ESI–TOF) [M–H]^–^ calc’d for C_7_H_10_NO_6_ 204.0514, found 204.0515.

Synthesis of D,L-methyloxaDAP and L,L-methyloxaDAP (Figure S4)

**(S)-3-((S)-2-Amino-2-carboxyethoxy)-2-hydroxy-2-methylpropanoic acid (7a) /**

**(*R*)-3-((*S*)-2-Amino-2-carboxyethoxy)-2-hydroxy-2-methylpropanoic acid (7b)**


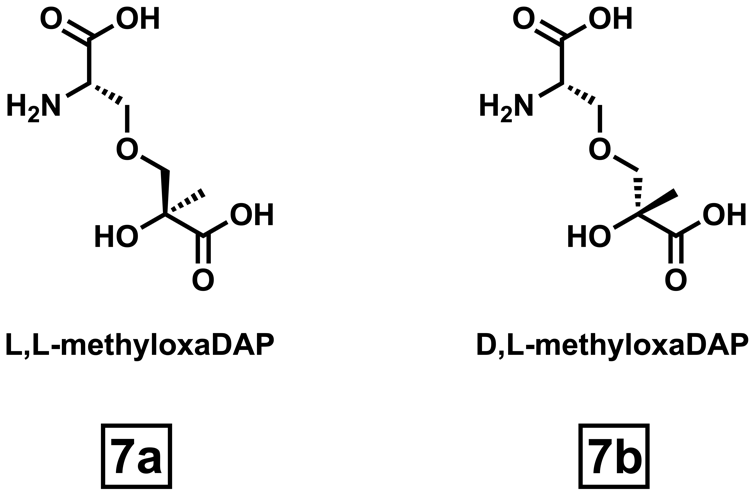


Compound **6a** or **6b** (20 mg, 0.045 mmol, 1.0 equiv) was dissolved in MeOH (1.0 mL), followed by addition of 10% Pd/C (9.6 mg, 9.0 µmol, 0.20 equiv). Triethylsilane (71 µL, 0.45 mmol, 10.0 equiv) was then added to the reaction mixture. The reaction was allowed to stir at room temperature for 3.5 hours. After that time, the crude reaction mixture was filtered with Celite and washed with MeOH (10 mL). The filtrate was concentrated *in vacuo* to give the pure product **7a** or **7b** (6.62 mg, 71%) without purification.

**7a:** ^1^H NMR (500 MHz, D_2_O) δ 4.45 (1 H, d, *J* = 8.0 Hz), 4.29 (1 H, d, *J* = 8.0 Hz), 4.29–4.26 (1 H, m), 4.08 (1 H, dd, *J* = 9.0, 3.0 Hz), 3.98 (1 H, dd, *J* = 9.0, 3.0 Hz), 1.36 (3 H, s). ^13^C NMR (125 MHz, D_2_O) δ 180.5, 169.8, 75.5, 72.5, 60.1, 55.7, 22.7. IR (cast film, 𝜈_max_ / cm^–1^) 3234, 2986, 1750, 1601, 1461, 1401, 1361, 1283, 1230. OR: [𝛼]_D_^26^ = –18.75 (c = 0.0080), H_2_O). HRMS (ESI–TOF) [M–H]^–^ calc’d for C_7_H_12_NO_6_ 206.0670, found 206.0665.

**7b:** ^1^H NMR (600 MHz, D_2_O) δ 4.45 (1 H, d, *J* = 11.3 Hz), 4.29 (1 H, d, *J* = 11.2 Hz), 4.15–4.12 (1 H, m), 4.05 (1 H, dd, *J* = 11.8, 4.0 Hz), 3.94 (1 H, dd, *J* = 11.8, 4.0 Hz), 1.39 (3 H, s). ^13^C NMR (125 MHz, D_2_O) δ 180.8, 169.0, 75.7, 72.7, 60.3, 55.7, 22.9. IR (cast film, 𝜈_max_ / cm^–1^) 3374, 3238, 2977, 2899, 1750, 1600 1460, 1401, 1232. OR: [𝛼]_D_^26^ = +18.06 (c = 0.0084, H_2_O). HRMS (ESI–TOF) [M–H]^–^ calc’d for C_7_H_12_NO_6_ 206.0670, found 206.0674.

Synthesis of D,L-α-methylDAP and L,L-α-methylDAP **(Figure S9)**

**Benzyl (2*S*,4*R*)-2-(*tert*-butyl)-4-((methylthio)methyl)-5-oxooxazolidine-3-carboxylate (9a) / benzyl (2*R*,4*S*)-2-(*tert*-butyl)-4-((methylthio)methyl)-5-oxooxazolidine-3-carboxylate (9b)**


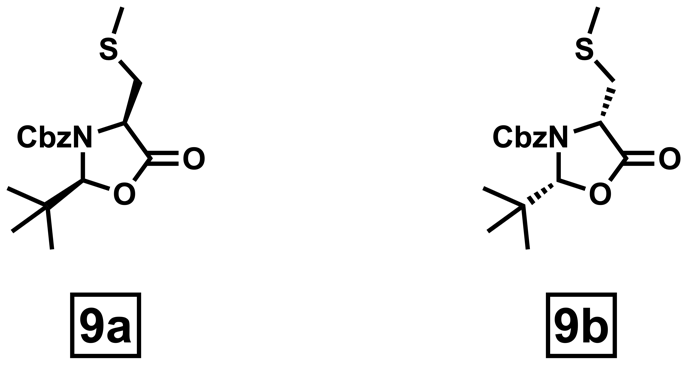


Optically pure S-methyl-cysteine (5.0 g, 37.0 mmol, 1.0 equiv) and NaOH (1.5 g, 37.0 mmol, 1.0 equiv) were dissolved in 300 mL anhydrous EtOH and the reaction mixture was stirred at rt until homogeneity. The reactiozn mixture was then concentrated *in vacuo,* and the residue was resuspended in 150 mL dry DCM. Pivalaldehyde (4.5 g, 51.8 mmol, 1.4 equiv) was then added dropwise, and the reaction mixture was heated to reflux with azeotropic removal of water for 16 h. The reaction mixture was then cooled to 0 °C and was added slowly dropwise with Cbz-Cl (10.7 g, 62.9 mmol, 1.7 equiv). The reaction mixture was then warmed to rt and was left to stir for 36 h. The reaction was then quenched with saturated NaHCO_3,_ and the organic layer was collected. The aqueous layer was further extracted with DCM (3 × 50 mL). The pooled organic layer was washed with brine (3 × 50 mL), dried over anhydrous Na_2_SO_4_, filtered, and concentrated in vacuo. The crude yellow oil was then purified via flash column chromatography (25% EtOAc in hexanes) to yield a light-yellow oil; R*_f_* = 0.75 (30% EtOAc in hexanes).

**9a:** Light yellow oil (32%); ^1^H NMR (500 MHz, CDCl_3_) δ 7.41–7.33 (m, 5 H), 5.55 (s, 1 H), 5.20 (A part of AB q, *J* = 11.9 Hz, 1 H), 5.15 (B part of AB q, *J* = 11.9 Hz), 4.50 (dd, *J* = 7.5, 6.0 Hz, 1 H), 3.00 (dd, *J* = 14.0, 8.0 Hz, 1 H), 2.86 (dd, *J* = 14.0, 5.5 Hz, 1 H), 2.11 (s, 3 H), 0.97 (s, 9 H); ^13^C NMR (125 MHz, CDCl_3_) δ 171.3, 155.9, 135.1, 128.8, 128.7, 96.3, 68.7, 57.2, 37.0, 36.8, 24.9, 16.1; FTIR (dry film, cm^–1^) 3068, 3032, 2973, 2940, 2874, 1793, 1724, 1482, 1309, 1129; OR [α]_D_^26^ = +30.5 (c = 1.44, DCM); HRMS (ESI-TOF) [M + Na]^+^ calc’d for C_17_H_23_NNaO_4_S 360.1240, found 360.1239.

**9b:** Light yellow oil (5.93 g, 47%); ^1^H NMR (500 MHz, CDCl_3_) δ 7.41–7.33 (m, 5 H), 5.55 (s, 1 H), 5.20 (A part of AB q, *J* = 11.9 Hz, 1 H), 5.15 (B part of AB q, *J* = 11.9 Hz), 4.50 (dd, *J* = 7.5, 6.0 Hz, 1 H), 3.00 (dd, *J* = 14.0, 8.0 Hz, 1 H), 2.86 (dd, *J* = 14.0, 5.5 Hz, 1 H), 2.11 (s, 3 H), 0.97 (s, 9 H); ^13^C NMR (125 MHz, CDCl_3_) δ 171.3, 155.9, 135.1, 128.8, 128.7, 96.3, 68.7, 57.2, 37.0, 36.8, 24.9, 16.1; FTIR (dry film, cm^–1^) 3068, 3032, 2973, 2940, 2874, 1793, 1724, 1482, 1309, 1129; OR [α]_D_^26^ = –30.5 (c = 1.44, DCM); HRMS (ESI-TOF) [M + Na]^+^ calc’d for C_17_H_23_NNaO_4_S 360.1240, found 360.1241.

**Benzyl (2*S*,4*R*)-2-(*tert*-butyl)-4-((methylsulfonyl)methyl)-5-oxooxazolidine-3-carboxylate (10a) / benzyl (2*R*,4*S*)-2-(*tert*-butyl)-4-((methylsulfonyl)methyl)-5-oxooxazolidine-3-carboxylate (10b)**

**
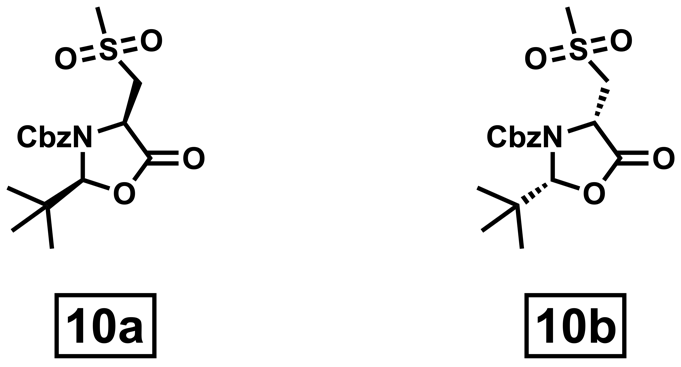
**

Compound **9a** or **9b** (1.2 g, 3.6 mmol, 1.0 equiv) was dissolved in 50 mL DCM. A solution of *m*CPBA (1.5 g, 8.9 mmol, 2.5 equiv) in 30 mL DCM was then added dropwise, and the resulting solution was stirred at rt for 24 h. The reaction was then quenched with saturated NaHCO_3_ and was extracted with DCM (3 × 10 mL). The pooled organic layer was then washed with brine (3 × 10 mL), dried over anhydrous Na_2_SO_4_, filtered, and concentrated *in vacuo*. The crude product was then purified via flash column chromatography (50% EtOAc in hexanes) to yield a clear, viscous oil; R*_f_* = 0.60 (50% EtOAc in hexanes).

**10a:** clear viscous oil (1.12 g, 85%). The characterization data matches what was reported on literature (Hsiao, Y. T., *et al.*, Organic Letters, 23(18), 7270–7273).

**10b:** clear viscous oil (1.06 g, 81%); ^1^H NMR (500 MHz, CDCl_3_) δ 7.42–7.36 (m, 5 H), 5.62 (s, 1 H), 5.26 (A part of AB q, *J* = 11.9 Hz, 1 H), 5.21 (B part of AB q, *J* = 11.9 Hz), 4.99 (dd, *J* = 7.9, 3.5 Hz, 1 H), 3.57 (dd, *J* = 15.1, 8.1, 1 H), 3.39 (dd, *J* = 15.2, 4.8 Hz), 3.11 (s, 3 H), 0.95 (s, 9 H); ^13^C NMR (125 MHz, CDCl_3_) δ 170.7, 155.3, 134.8, 128.9, 128.9, 128.8, 96.9, 69.0, 60.4, 57.3, 53.5, 42.6, 37.2, 24.6; FTIR (dry film, cm^–1^) 3068, 3032, 2973, 2940, 2874, 1793, 1724, 1482, 1395, 1129; OR [α]_D_^26^ = –34.9 (c = 0.83, DCM); HRMS (ESI-TOF) [M + Na]^+^ calc’d for C_17_H_23_NNaO_6_S 392.1138, found 392.1145.

**Benzyl (*S*)-2-(*tert*-butyl)-4-methylene-5-oxooxazolidine-3-carboxylate (11a) / Benzyl (*R*)-2-(*tert*-butyl)-4-methylene-5-oxooxazolidine-3-carboxylate (11b)**


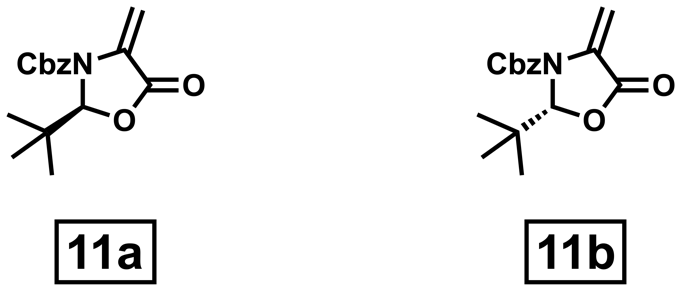


Compound **10a** or **10b** (0.95 g, 2.6 mmol, 1.0 equiv) was dissolved in 20 mL DCM, and was cooled to 0 °C. DBU (0.45 g, 3.0 mmol, 1.2 equiv) was then added dropwise and the reaction mixture was stirred at 0 °C for 30 min. The reaction was then quenched with 20.0 mL H_2_O, and the resulting organic layer was collected. The aqueous layer was then extracted with DCM (3 x 10 mL). The pooled organic layers were then washed with brine (3 × 10 mL), dried over saturated Na_2_SO_4_, filtered, and concentrated *in vacuo.* The crude product was purified using a flash column chromatography (30% EtOAc in hexanes) to give a white solid; R*_f_* = 0.62 (30% EtOAc in hexanes).

**11a:** white solid (0.64 g, 86%). The characterization data matches what was reported in the literature (Hsiao, Y. T., *et al.*, Organic Letters, 23(18), 7270–7273).

**11b:** white solid (0.64 g, 86%); ^1^H NMR (500 MHz, CDCl_3_) δ 7.41–7.36 (m, 5 H), 5.72 (s, 1 H), 5.68 (app s, 2 H), 5.27 (A part of AB q, *J* = 12.1 Hz, 1 H), 5.25 (B part of AB quartet, *J* = 12.1 Hz, 1 H), 0.93 (s, 9 H); ^13^C NMR (125 MHz, CDCl_3_) δ 164.6, 152.4, 134.7, 130.2, 128.9, 128.8, 128.7, 104.4, 94.0, 68.8, 38.7, 24.4; FTIR (dry film, cm^–1^) 3067, 3034, 2971, 2910, 2875, 1794, 1724, 1587, 1497, 1268; OR [α]_D_^26^ = +35.9 (c = 0.43, DCM); HRMS (ESI-Orbitrap) [M + H]^+^ calc’d for C_16_H_20_NO_4_ 290.1387, found 290.1386.

**1-(*Tert-*butyl)-5-(1,3-dioxoisoindolin-2-yl)-(*tert*-butoxycarbonyl)-L-glutamate (12)**


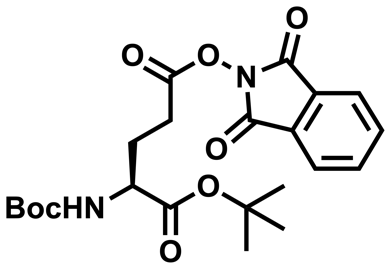


Boc-L-Glu-α-OtBu (1.0 g, 3.5 mmol, 1.0 equiv) was dissolved in 17.5 mL THF and was added with *N*-hydroxythalimide (0.63 g, 3.8 mmol, 1.1 equiv). DMAP (43 mg, 0.35 mmol, 0.1 equiv) and DIC (0.67 g, 5.2 mmol, 1.5 equiv) were then added sequentially, and the reaction mixture was stirred at rt for 16 h. The reaction mixture was then vacuum filtered to remove the urea side product, and the filtrate was then concentrated *in vacuo*. The crude product was purified using flash column chromatography (30 % EtOAc in hexanes) to yield a white solid; R*_f_* = 0.48 (40% EtOAc in hexanes). The characterization data matches what was reported in the literature (Hsiao, Y. T., *et al.*, Organic Letters, 23(18), 7270–7273).

**Benzyl (2*S,*4*S*)*-*4-((*S*)-5-(*tert*-butoxy)-4-((*tert*-butoxycarbonyl)amino)-5-oxopentyl)-2-(*tert-*butyl)-5-oxooxazolidine-3-carboxylate (13a) / Benzyl (2*R,*4*R*)*-*4-((*S*)-5-(*tert*-butoxy)-4-((*tert*-butoxycarbonyl)amino)-5-oxopentyl)-2-(*tert-*butyl)-5-oxooxazolidine-3-carboxylate (13b)**

**
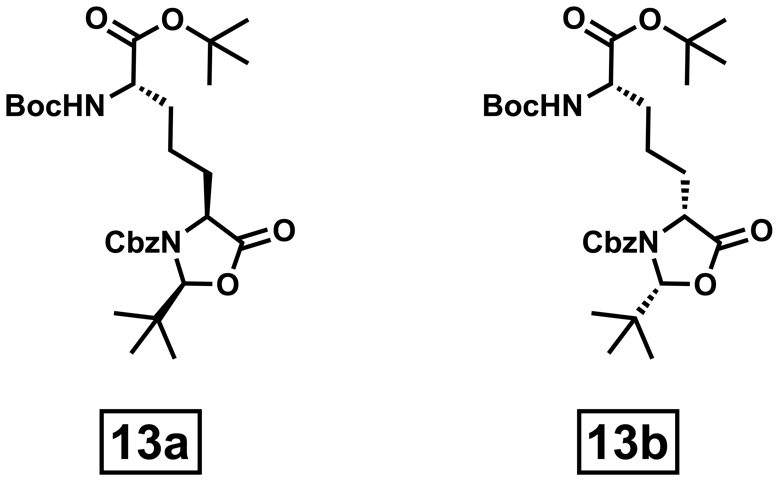
**

Compound **11a** or **11b** (0.29 g, 1.0 mmol, 1.0 equiv) was weighed into a round bottom flask with compound **12** (0.90 g, 2.0 mmol, 2.0 equiv), LiCl (0.13 g, 3.0 mmol, 3.0 equiv), Zn (0.13 g, 2.0 mmol, 2.0 equiv), and Ni(ClO_4_)_2_∙6H_2_O (0.11 g, 0.3 mmol, 0.3 equiv). The reaction flask was purged with Ar, followed by the addition of DMSO (3.0 mL). The reaction mixture was stirred at rt for 16 h. After that, the reaction was quenched with saturated NH_4_Cl:H_2_O (1:1). The reaction mixture was then extracted with EtOAc (3 × 15 mL). The pooled organic layer was then washed with saturated NaHCO_3_ (1 × 15 mL) and brine (1 × 15 mL), dried over anhydrous Na_2_SO_4_, filtered, and concentrated *in vacuo*. The resulting crude oil was then purified using flash column chromatography (20% EtOAc in hexanes) to yield a viscous colorless oil.

**13a:** colorless, clear oil (0.18 g, 79%). The characterization data matches what was reported in the literature (Hsiao, Y. T., *et al.*, Organic Letters, 23(18), 7270–7273).

**13b:** colorless oil (55%); ^1^H NMR (500 MHz, CDCl_3_) δ 7.43–7.32 (m, 5 H), 5.54 (s, 1 H), 5.19 (A part of AB q, *J* = 12.0 Hz, 1 H), 5.14 (B part of AB q, *J* = 12.0 Hz, 1 H), 5.02 (br d, *J* = 3.9 Hz, 1 H), 4.24 (app t, *J* = 7.1 Hz, 1 H), 4.19–4.16 (m, 1 H), 1.98–1.87 (m, 1 H), 1.84–1.75 (m, 2 H), 1.74–1.60 (m, 2 H), 1.55–1.50 (m, 1 H), 1.45 (s, 18 H), 0.95 (s, 9 H); ^13^C NMR (125 MHz, CDCl_3_) δ 172.5, 171.8, 156.1, 155.4, 135.3, 128.8, 128.7, 128.6, 96.4, 81.9, 79.7, 68.5, 56.9, 53.7, 36.9, 32.9, 32.4, 28.4, 28.0, 25.0, 21.9 FTIR (dry film, cm^–1^) 3373, 2976, 2942, 2872, 1793, 1717, 1499, 1155; OR [α]_D_^26^ = –4.75 (c = 0.49, DCM); HRMS (ESI-TOF) [M + Na]^+^ calc’d for C_29_H_44_N_2_NaO_8_ 571.2990, found 571.2984.

**Benzyl (2*S,*4*S*)-4-((S)-5-*tert*-butoxy)-4-((*tert*-butoxycarbonyl)amino)-5-oxopentyl)-2-(*tert*-butyl)-4-methyl-5-oxooxazolidine-3-carboxylate (14a) / Benzyl (2*R,*4*R*)-4-((S)-5-*tert*-butoxy)-4-((*tert*-butoxycarbonyl)amino)-5-oxopentyl)-2-(*tert*-butyl)-4-methyl-5-oxooxazolidine-3-carboxylate (14b)**

**
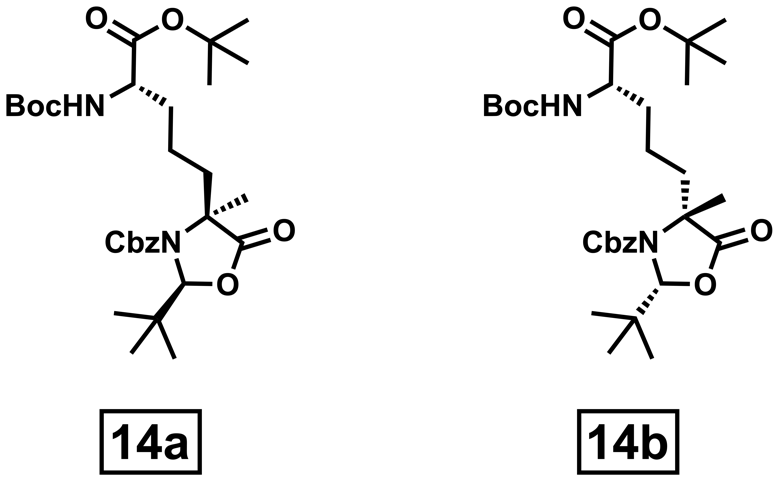
**

Compound **13a** or **13b** (0.175 g, 0.319 mmol, 1.00 equiv) was dissolved in freshly distilled THF (3.0 mL) in a flame-dried round bottom flask and cooled to -78 °C under argon. KHMDS (1.0 M in THF, 0.8 mL) was added to the solution dropwise, and the reaction mixture was stirred for 20 minutes. Methyl iodide (24 µL, 0.383 mmol, 1.20 equiv) was then added in one portion, and the mixture was stirred until completion, approximately 3 hours. Upon completion, the reaction mixture was quenched with aq. sat. NH_4_Cl, and allowed to warm to rt. The reaction mixture was extracted with EtOAc (3 x 10 mL), and organic layers combined and washed with H_2_O (10 mL) and brine (10 mL). The organic layer was then dried over Na_2_SO_4_, filtered, and concentrated. The crude product was obtained as a yellow foam (0.152 g), then purified by flash column chromatography (20% EtOAc in hexanes). The desired fractions were combined and concentrated to yield a clear, colorless oil.

**14a:** clear, colorless oil (0.032 g, 18%); ^1^H NMR (500 MHz, CDCl_3_) δ 7.40–7.32 (m, 5 H), 5.56 (s, 1 H), 5.20–5.12 (m, 2 H), 5.00 (d, *J* = 8.1 Hz, 1 H), 4.19–4.13 (m, 1 H), 1.92–1.84 (m, 2 H), 1.77–1.69 (m, 2 H), 1.63–1.53 (m, 2 H), 1.49 (s, 3 H), 1.443 (s, 9 H), 1.438 (s, 9 H), 0.94 (s, 9 H); ^13^C NMR (125 MHz, CDCl_3_) δ 174.9, 171.8, 155.3, 135.2, 128.72, 128.67, 128.6, 94.4, 81.9, 79.6, 67.9, 62.6, 53.8, 38.0, 37.5, 33.3, 28.4, 28.0, 27.6, 25.5, 24.4, 20.2; FTIR (cast film, cm^–1^) 3432, 3373, 2977, 2940, 2880, 1794, 1717, 1499, 1457, 1393, 1368, 1342, 1250, 1229, 1155; OR [α]_D_^26^ = +28.32 (c = 0.19, DCM); HRMS (ESI-TOF) [M + Na]^+^ calc’d for C_30_H_46_N_2_NaO_8_ 585.3146, found 585.3143.

**14b:** clear, colorless oil (10%); ^1^H NMR (500 MHz, CDCl_3_) δ 7.41–7.34 (m, 5 H), 5.57 (s, 1 H), 5.18–5.13 (m, 2 H), 4.99 (app d, *J* = 7.6 Hz, 1 H), 4.18–4.17 (m, 1 H), 2.03–1.89 (m, 2 H), 1.78–1.57 (m, 4 H), 1.56 (s, 3 H), 1.45 (s, 9 H), 1.44 (s, 9 H), 0.95 (s, 9 H); ^13^C NMR (125 MHz, CDCl_3_) δ 175.0, 171.8, 155.3, 135.2, 128.8, 128.73, 128.70, 94.5, 81.9, 79.6, 67.9, 62.6, 53.7, 38.0, 37.5, 33.2, 28.4, 28.1, 28.0, 25.5, 24.4, 20.2; FTIR (dry film, cm^–1^) 3379, 2966, 2924, 2851, 1794, 1716, 1498, 1457, 1339, 1230; OR [α]_D_^26^ = –3.46 (c = 0.13, DCM); HRMS (ESI-TOF) [M + Na]^+^ calc’d for C_30_H_46_N_2_NaO_8_ 585.3146, found 585.3147.

**(2*S*,6*S*)-2,6-diamino-2-methylheptanedioic acid (15a) / (2*R*,6*S*)-2,6-diamino-2-methylheptanedioic acid (15b)**

**
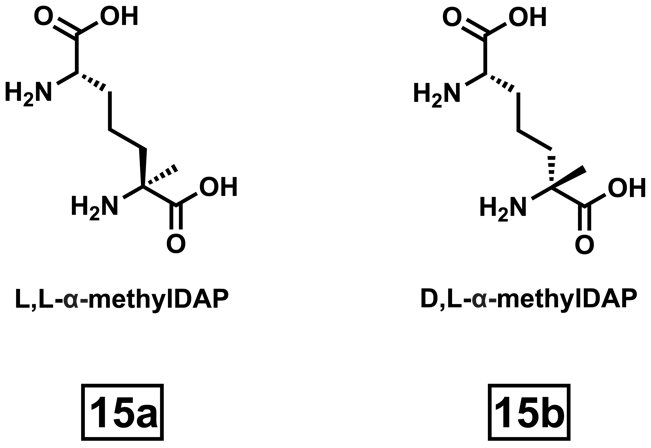
**

Compound **14a** or **14b** (0.030 g, 0.053 mmol, 1.00 equiv) was dissolved in 1,4-dioxane (1.0 mL) and conc. HCl (1.0 mL) and the mixture was heated to 80 °C and stirred for approximately 4 hours. The reaction mixture was concentrated to remove the solvent, and redissolved in H_2_O. This was then frozen and lyophilized overnight to yield an off-white solid.

**15a:** off-white solid (0.011 g, >99%). Characterization data matches that of the literature (Paradisi, F., *et al.*, (2002), 13(5), 497–502). ^1^H NMR (700 MHz, D_2_O) δ 4.05 (t, *J* = 6.2 Hz, 1 H), 2.05–1.91 (m, 4 H), 1.60 (s, 3 H), 1.59–1.54 (m, 1 H), 1.52–1.47 (m, 1 H); ^13^C NMR (125 MHz, D_2_O) δ 175.4, 173.3, 63.5, 53.9, 37.2, 30.7, 22.8, 20.0

**15b:** off-white solid (>99%); ^1^H NMR (500 MHz, D_2_O) δ 3.98 (t, *J* = 6.2 Hz, 1 H), 2.02–1.92 (m, 4 H), 1.67–1.61 (m, 1 H), 1.58 (s, 3 H), 1.45–1.39 (m, 1 H); ^13^C NMR (125 MHz, D_2_O) δ 175.8, 173.2, 61.4, 54.2, 37.3, 30.9, 22.7, 20.2, FTIR (dry film, cm^–1^) 3381, 2965, 1724, 1621, 1223; OR [α]_D_^26^ = +0.79 (c = 0.13, H_2_O); HRMS (ESI-TOF) [M – H]^–^ calc’d for C_8_H_15_N_2_O_4_ 203.1037, found 203.1030.

**NMR Characterization Data**

**7a - ^1^H NMR (500 MHz, D_2_O)**

**
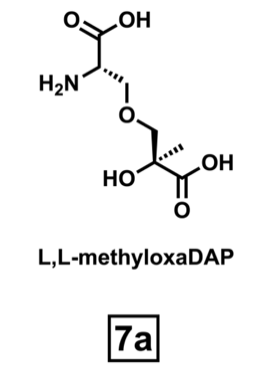

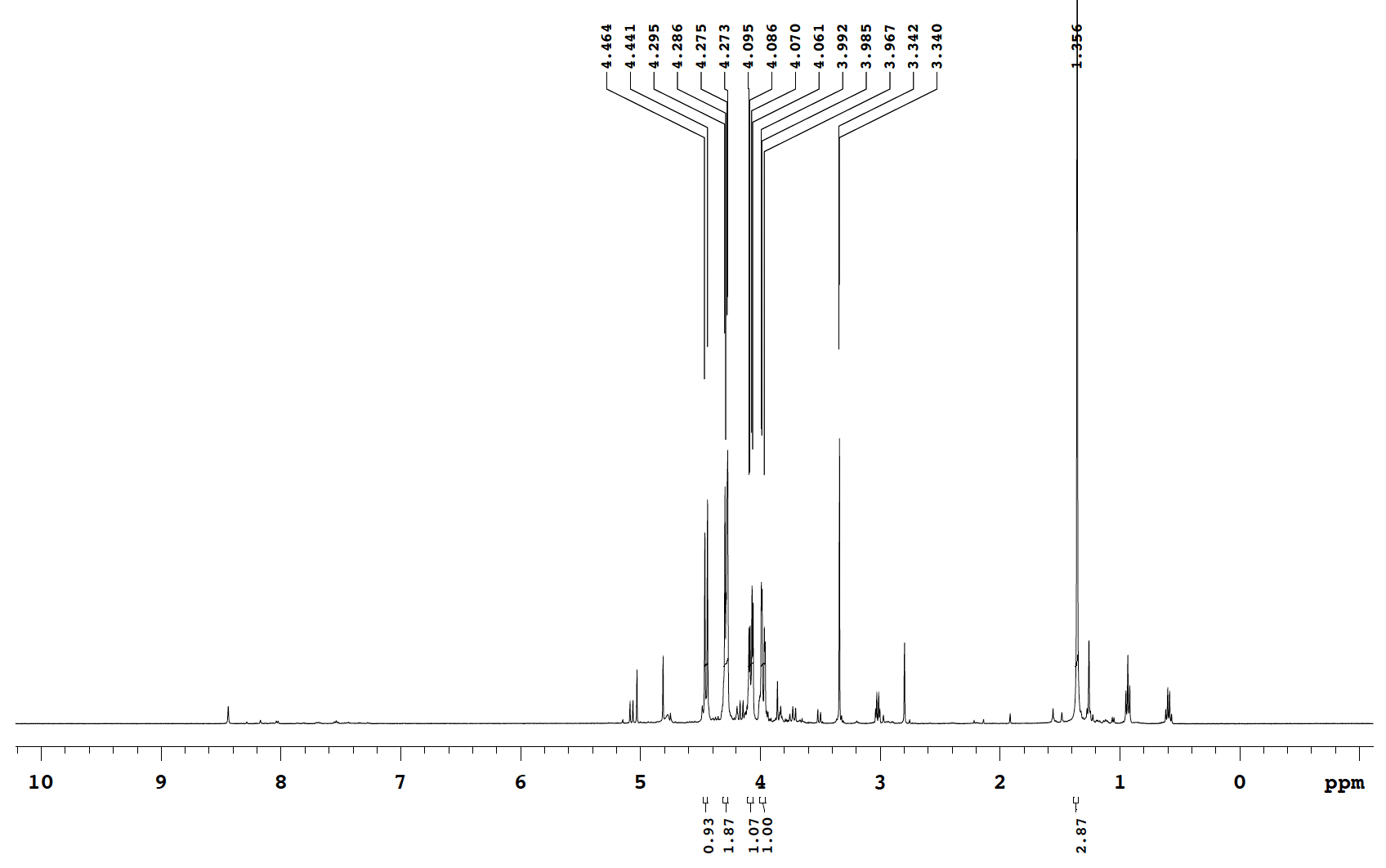
**

**7a – ^13^C NMR (125 MHz, D_2_O)**

**
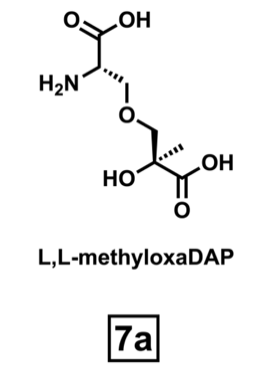

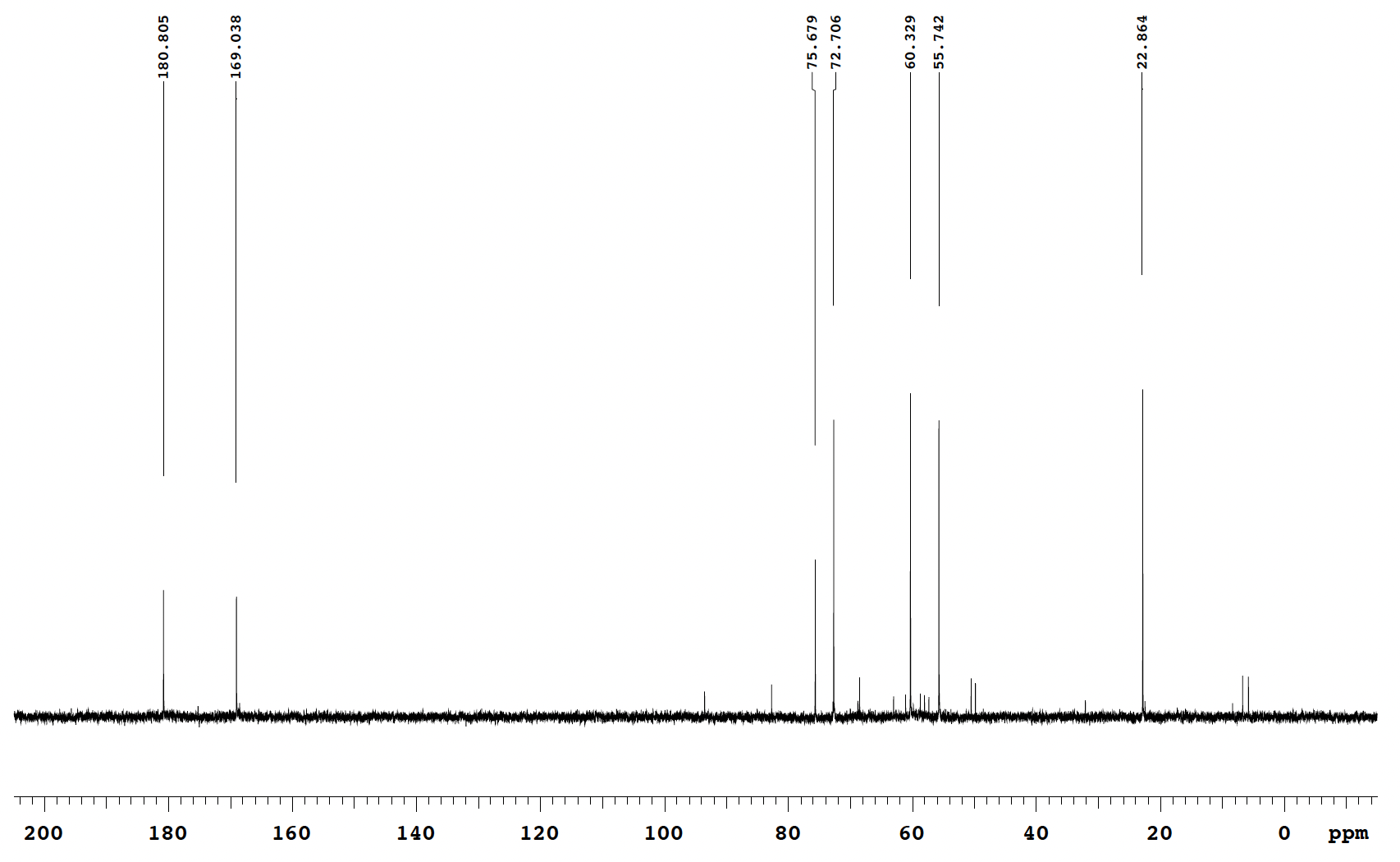
**

**7b - ^1^H NMR (500 MHz, D_2_O)**


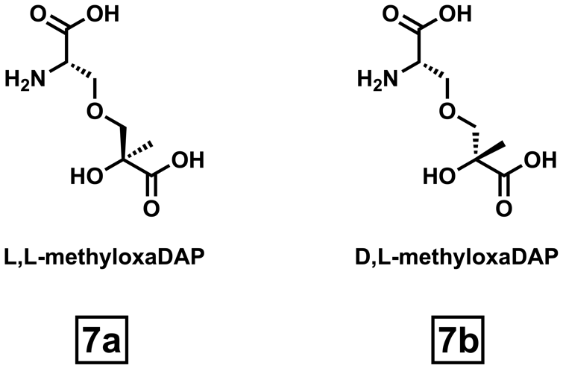

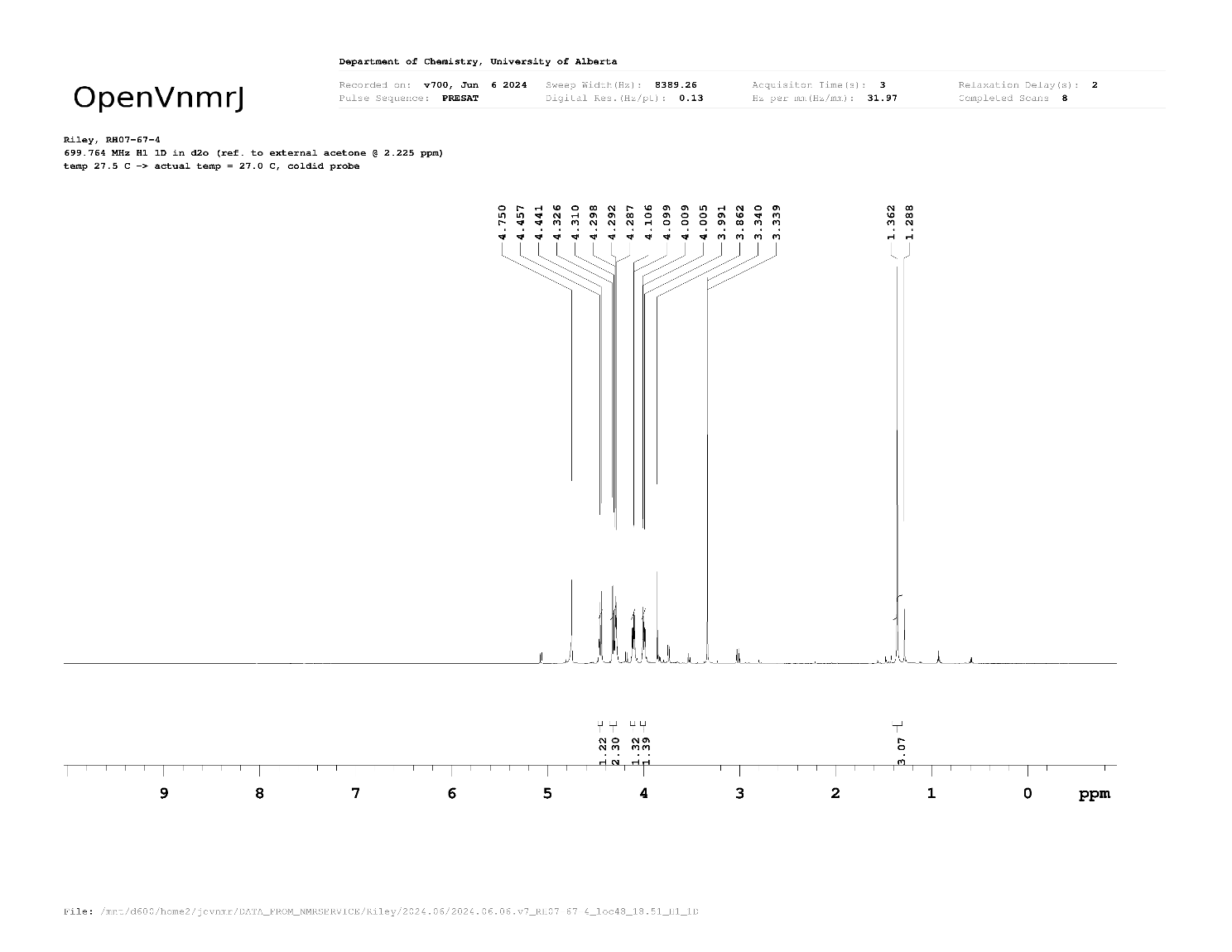


**7b – ^13^C NMR (125 MHz, D_2_O)**


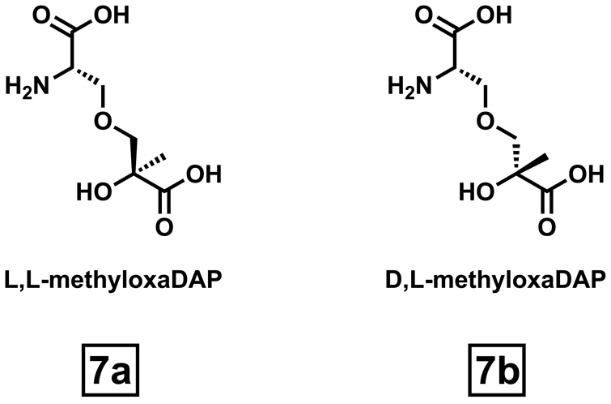

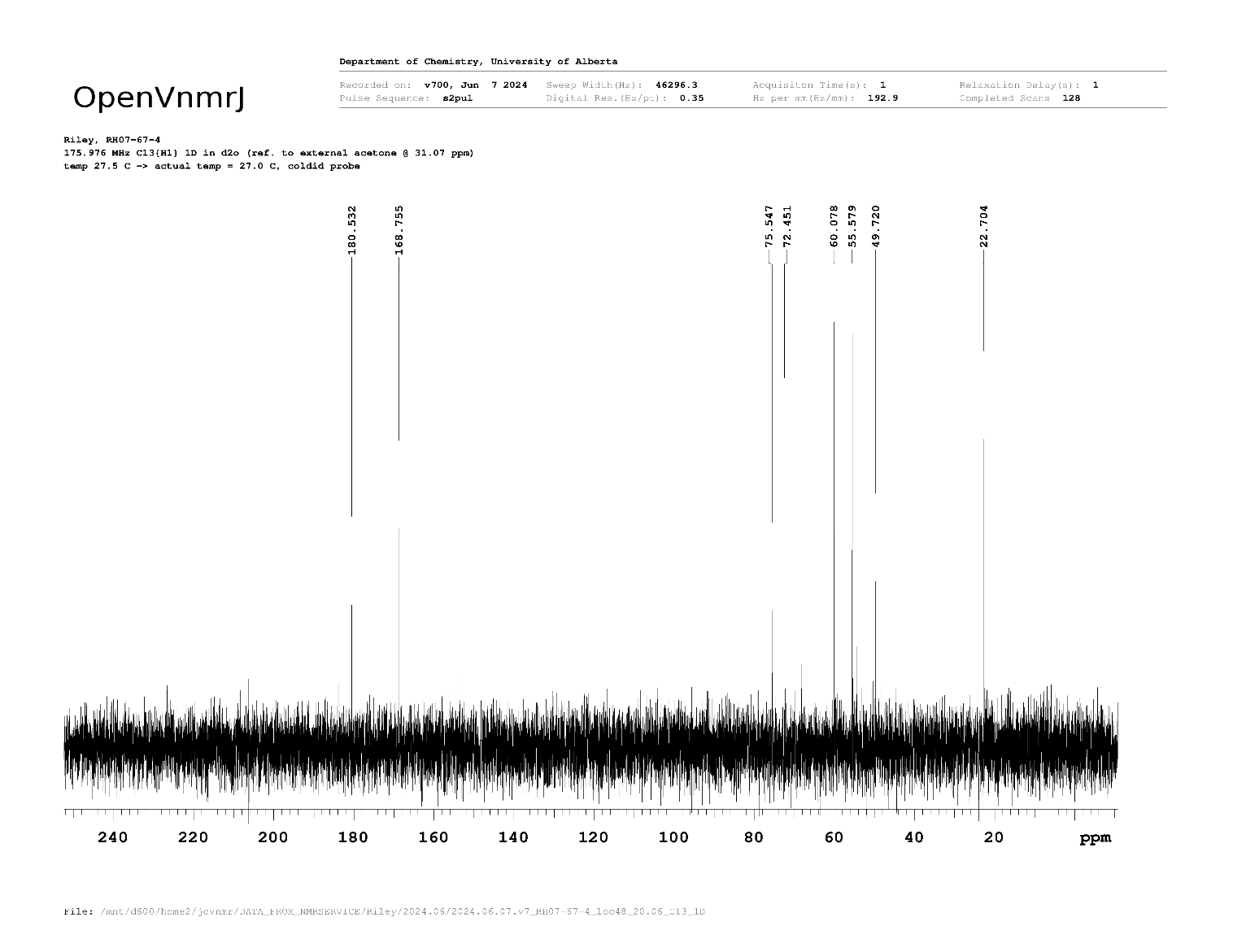


**9b – ^1^H NMR (500 MHz, CDCl_3_)**


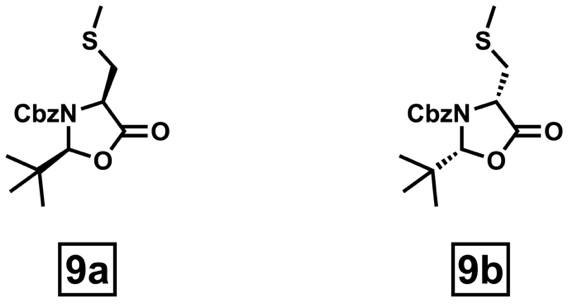

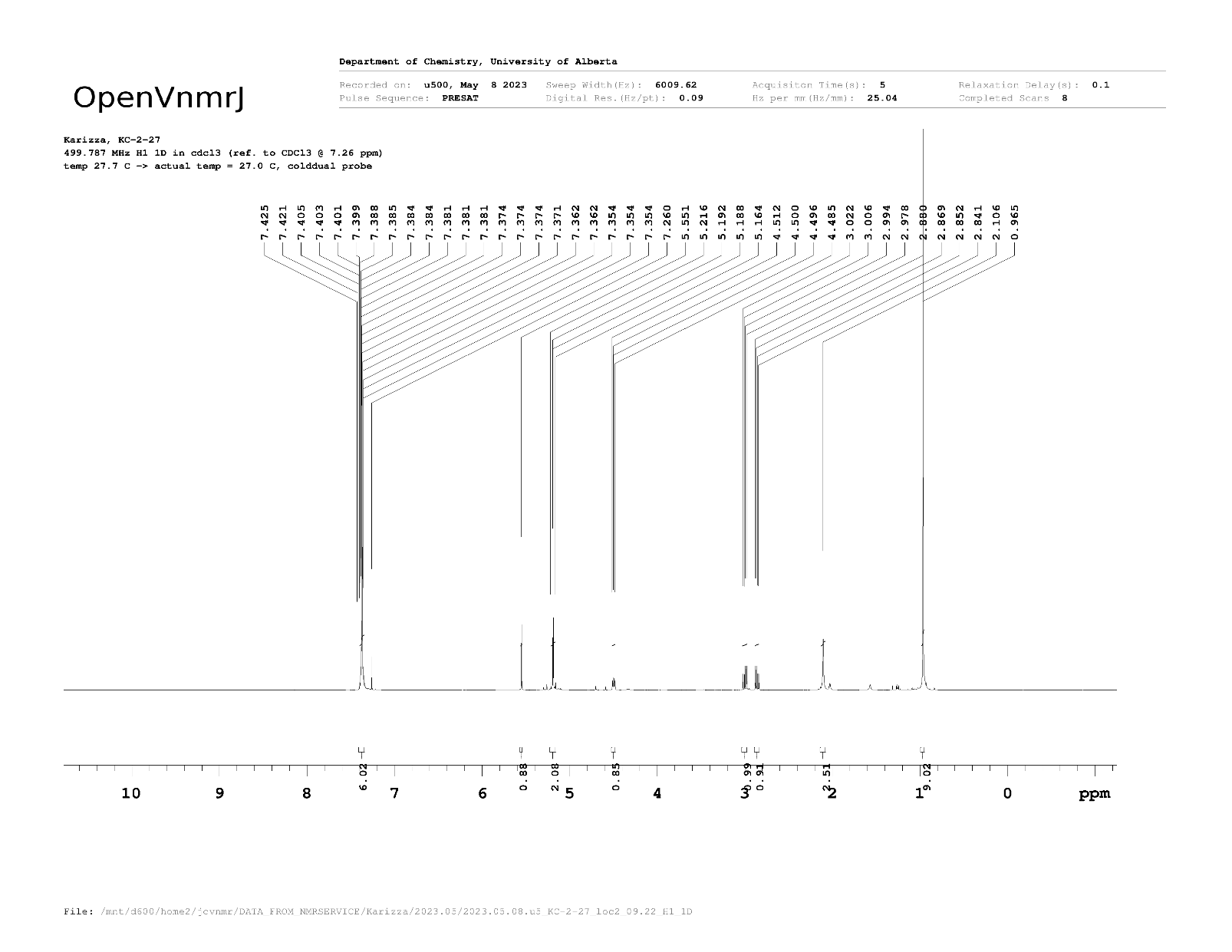


**9b – ^13^C NMR (125 MHz, CDCl_3_)**


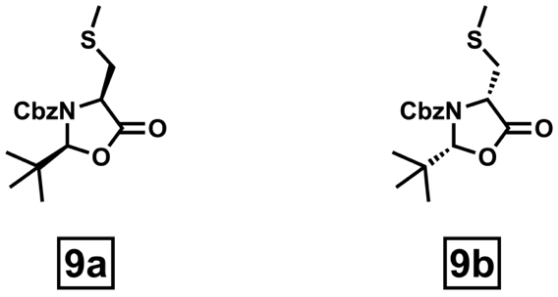

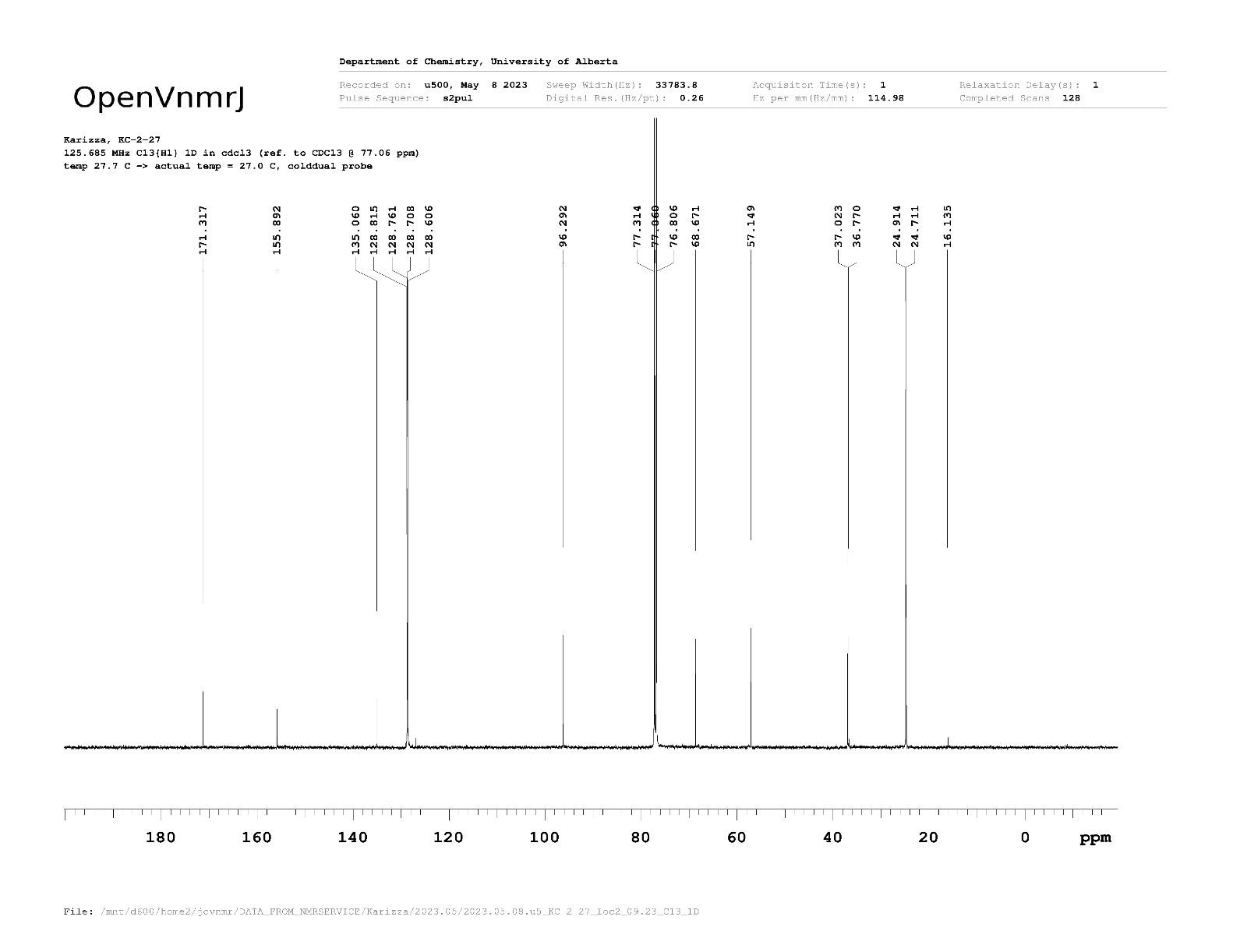


**10b – ^1^H NMR (500 MHz, CDCl_3_)**


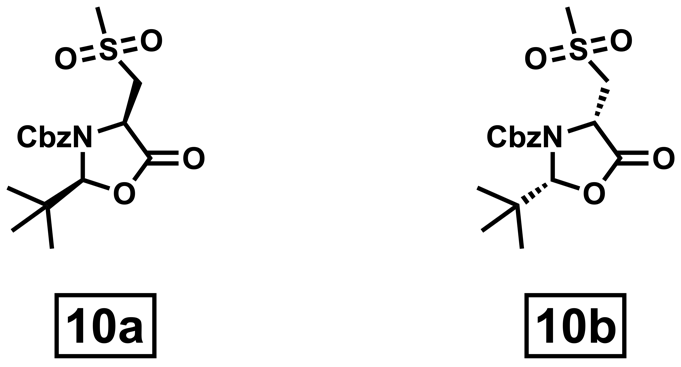

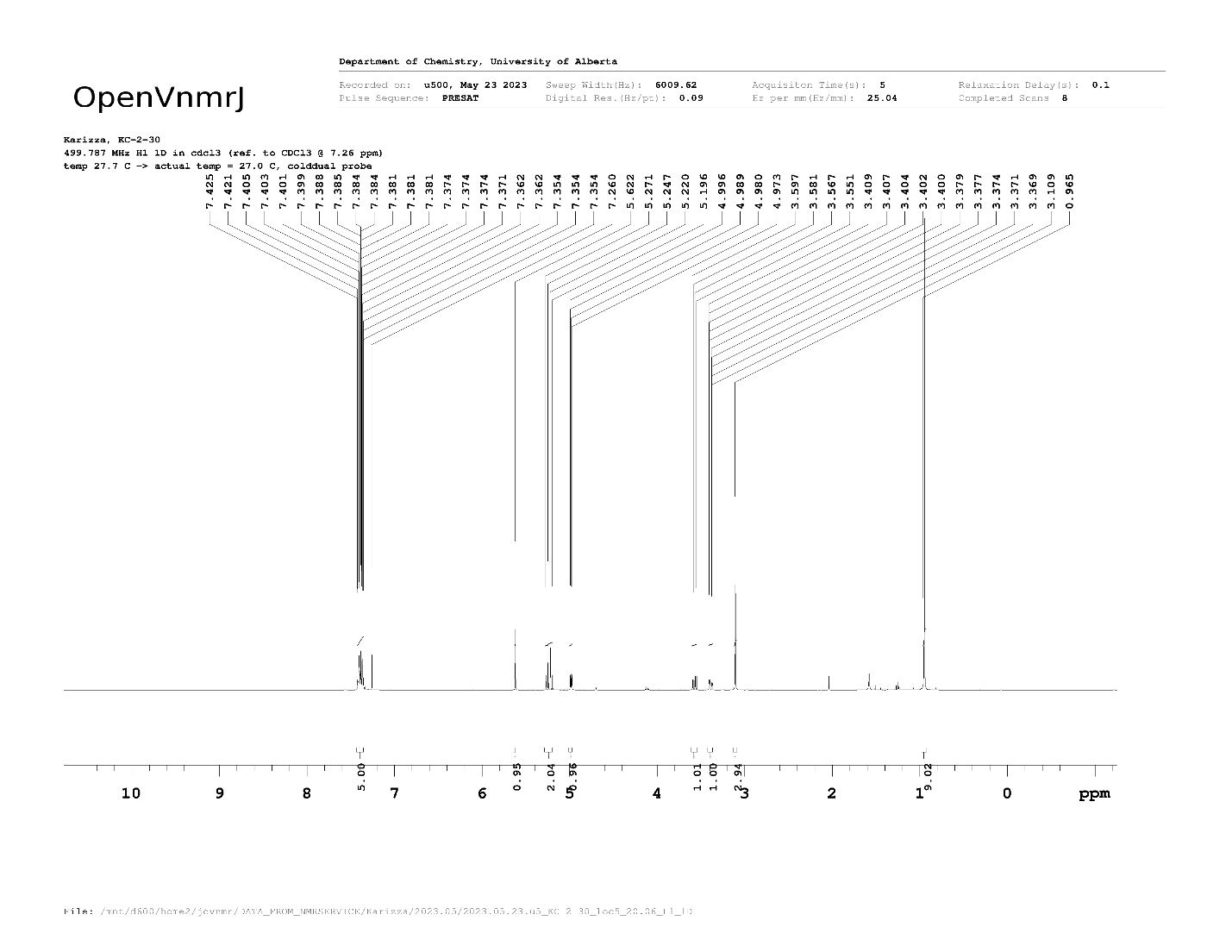


**10b – ^13^C NMR (125 MHz, CDCl_3_)**


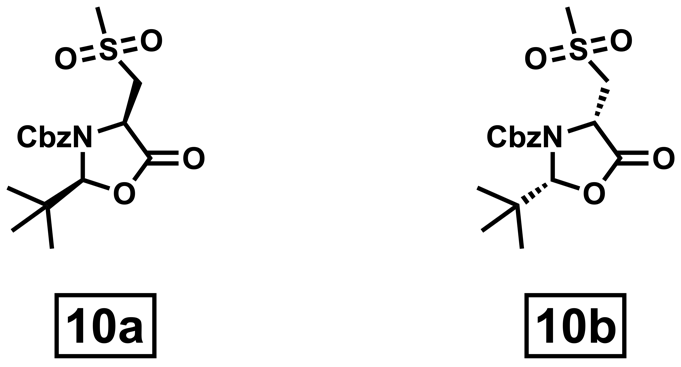

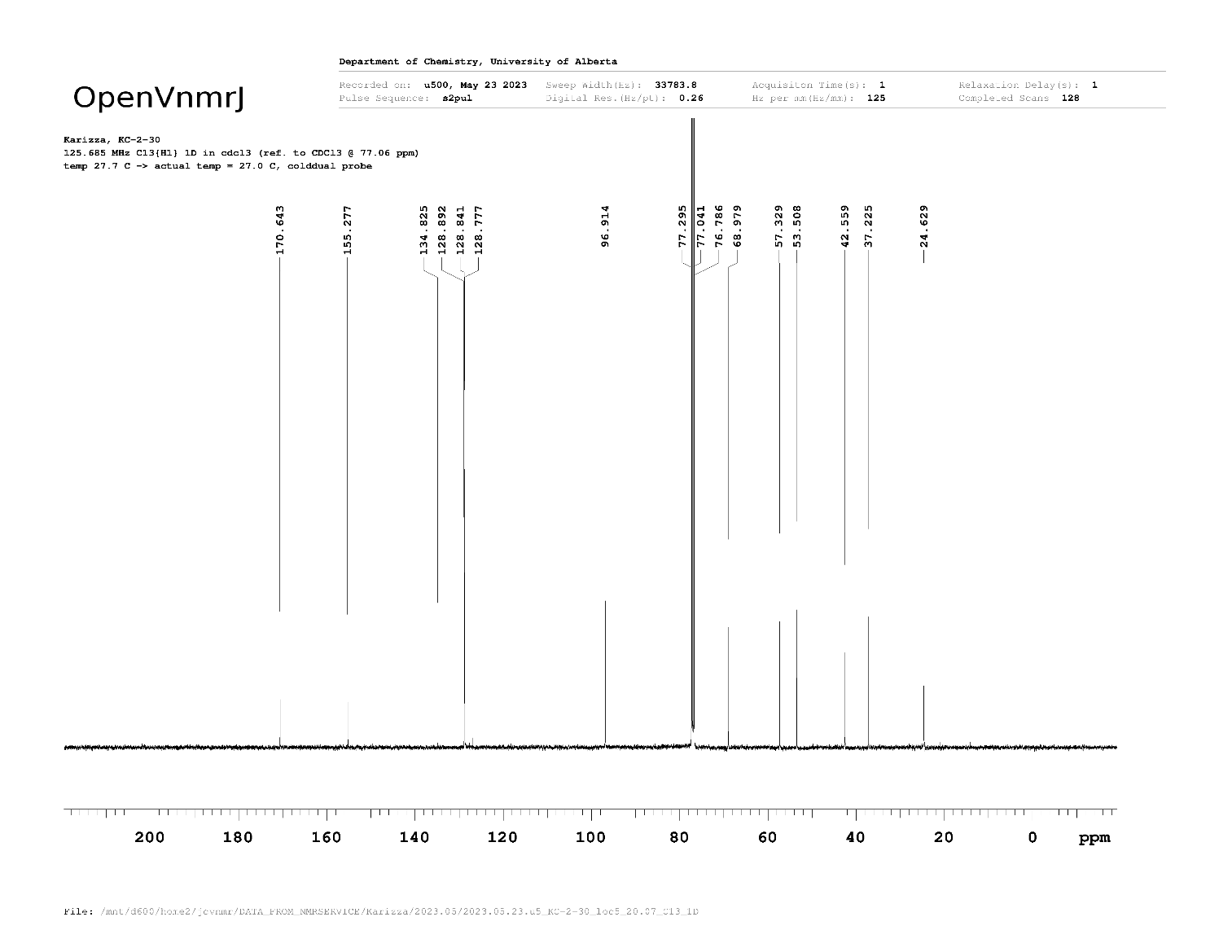


**11b - ^1^H NMR (500 MHz, CDCl_3_)**


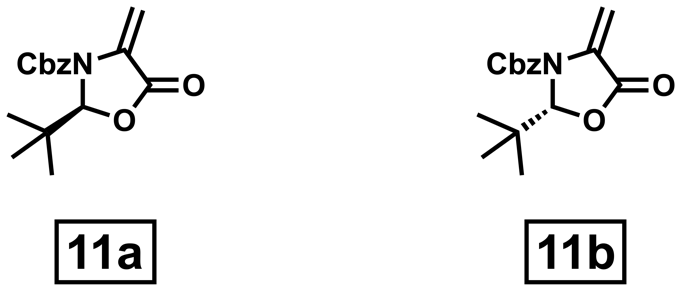

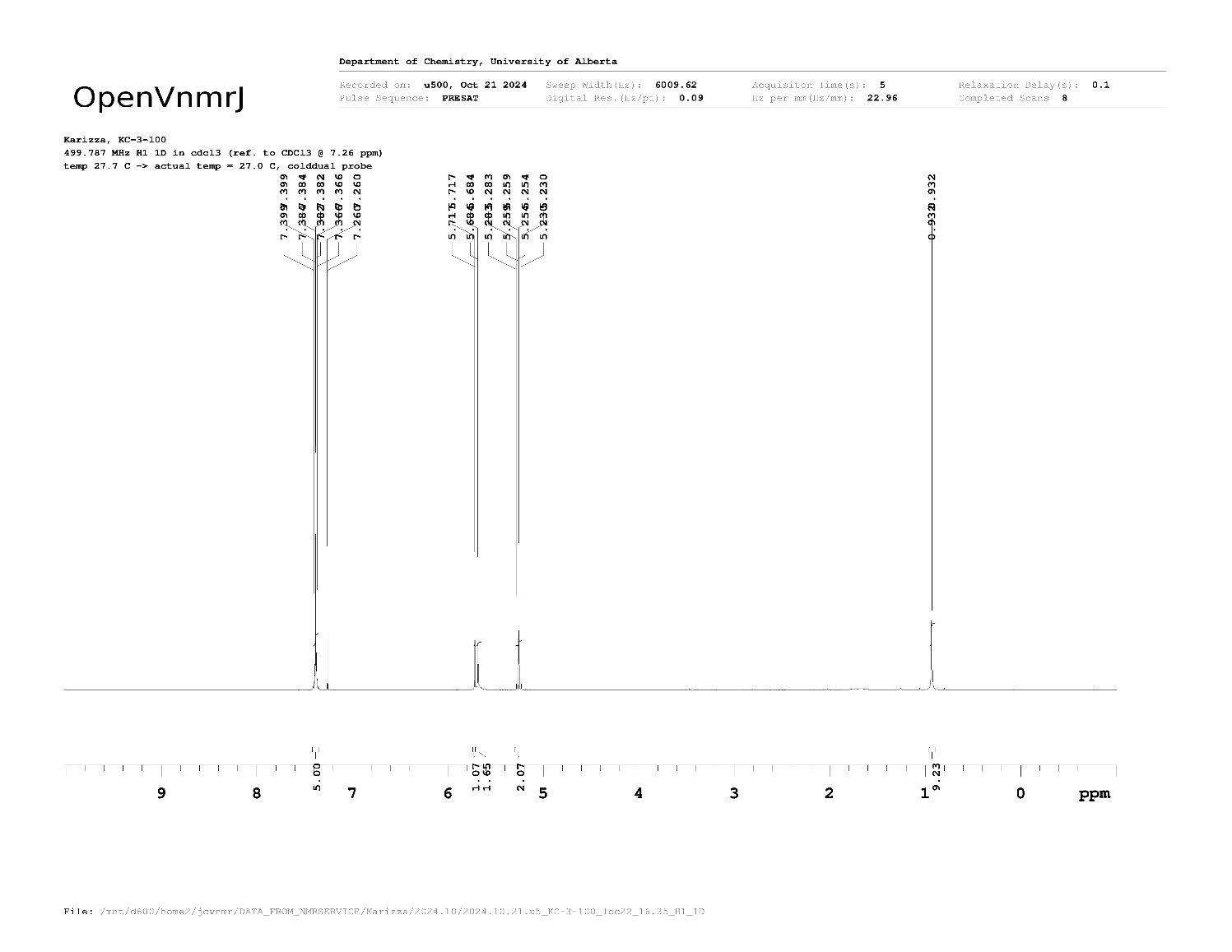


**11b – ^13^C NMR (125 MHz, CDCl_3_)**


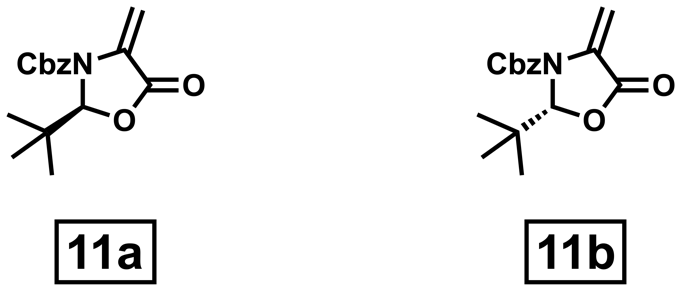

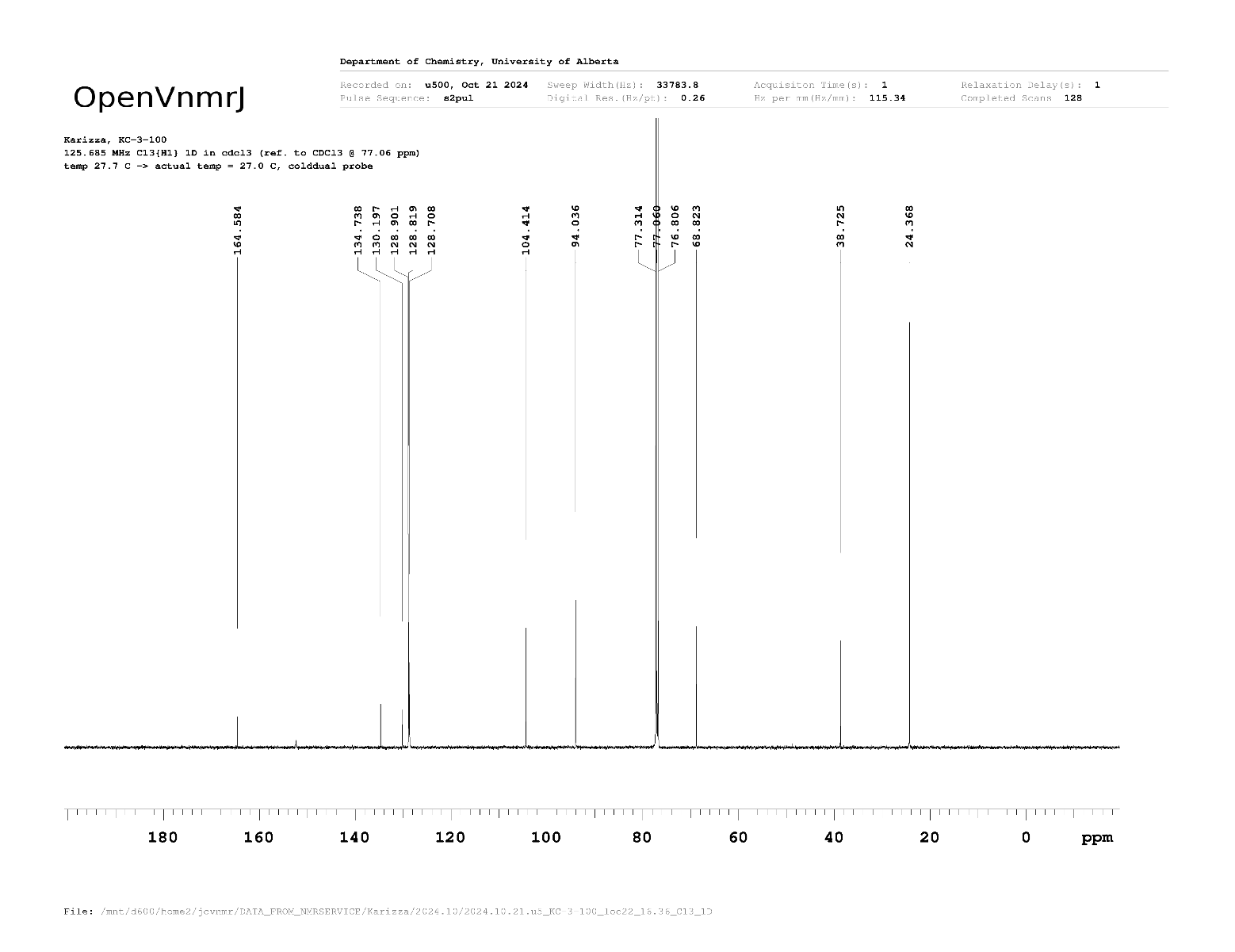


**12 - ^1^H NMR (500 MHz, CDCl_3_)**


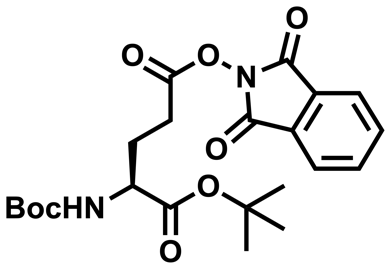

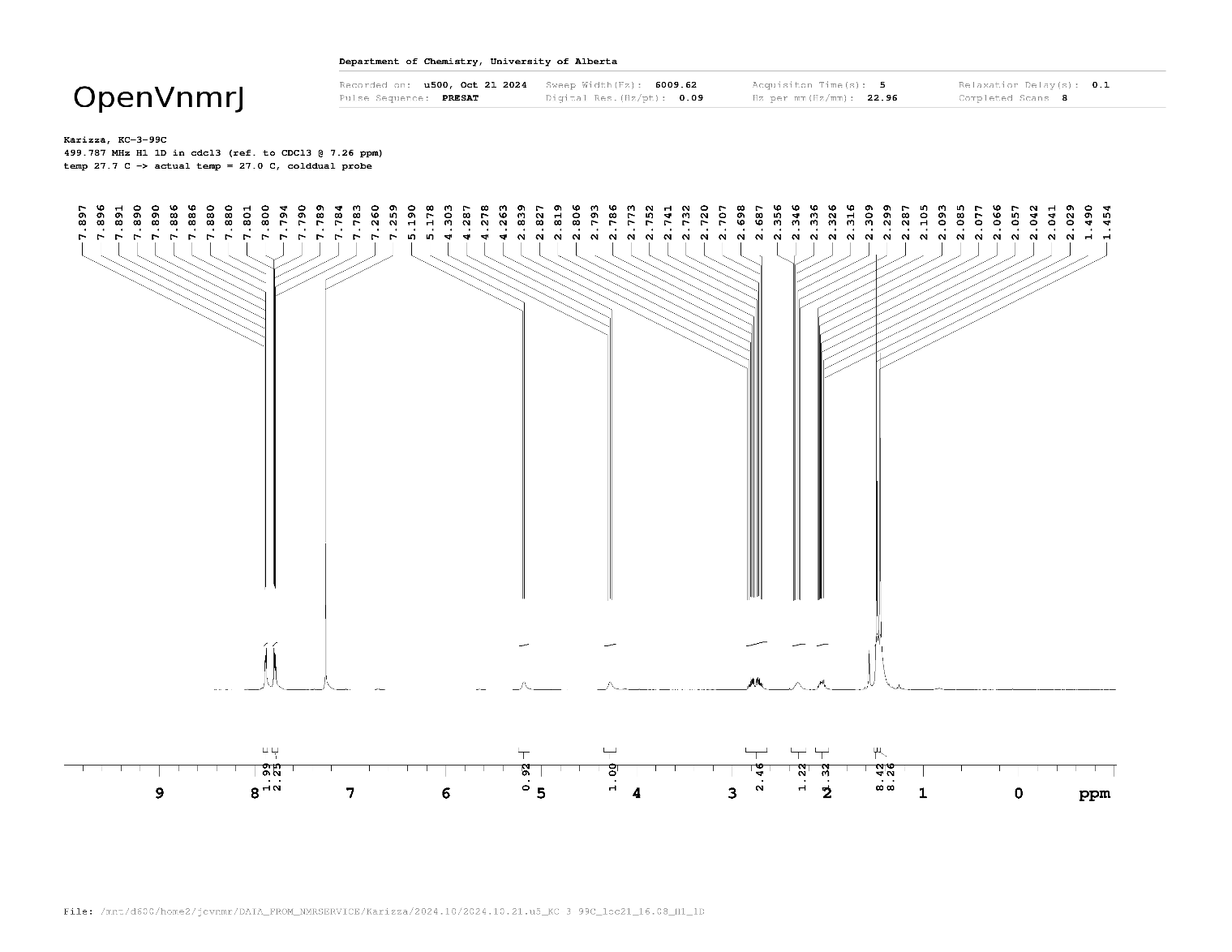


**12 – ^13^C NMR (125 MHz, CDCl_3_)**


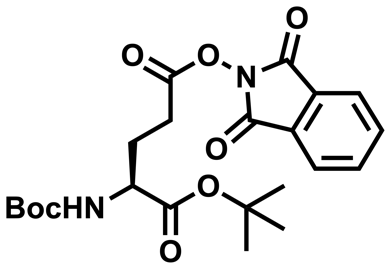

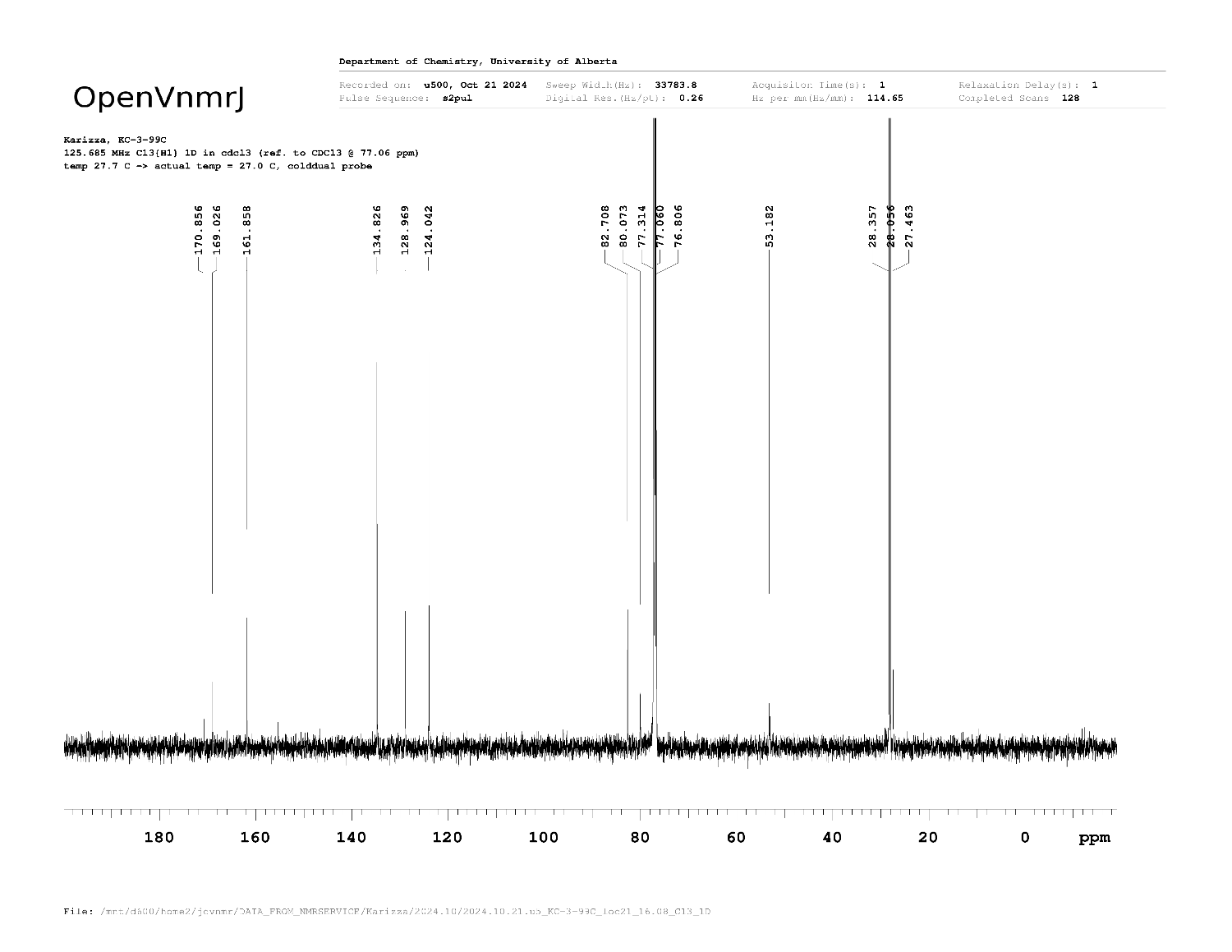


**13a - ^1^H NMR (500 MHz, CDCl_3_)**


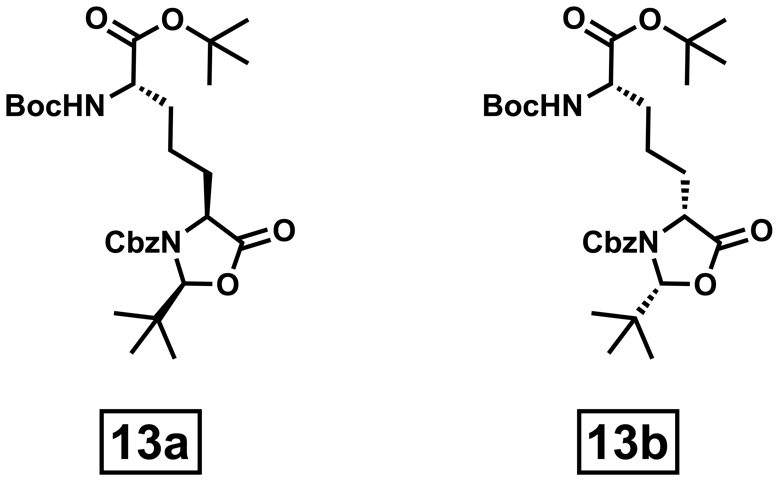

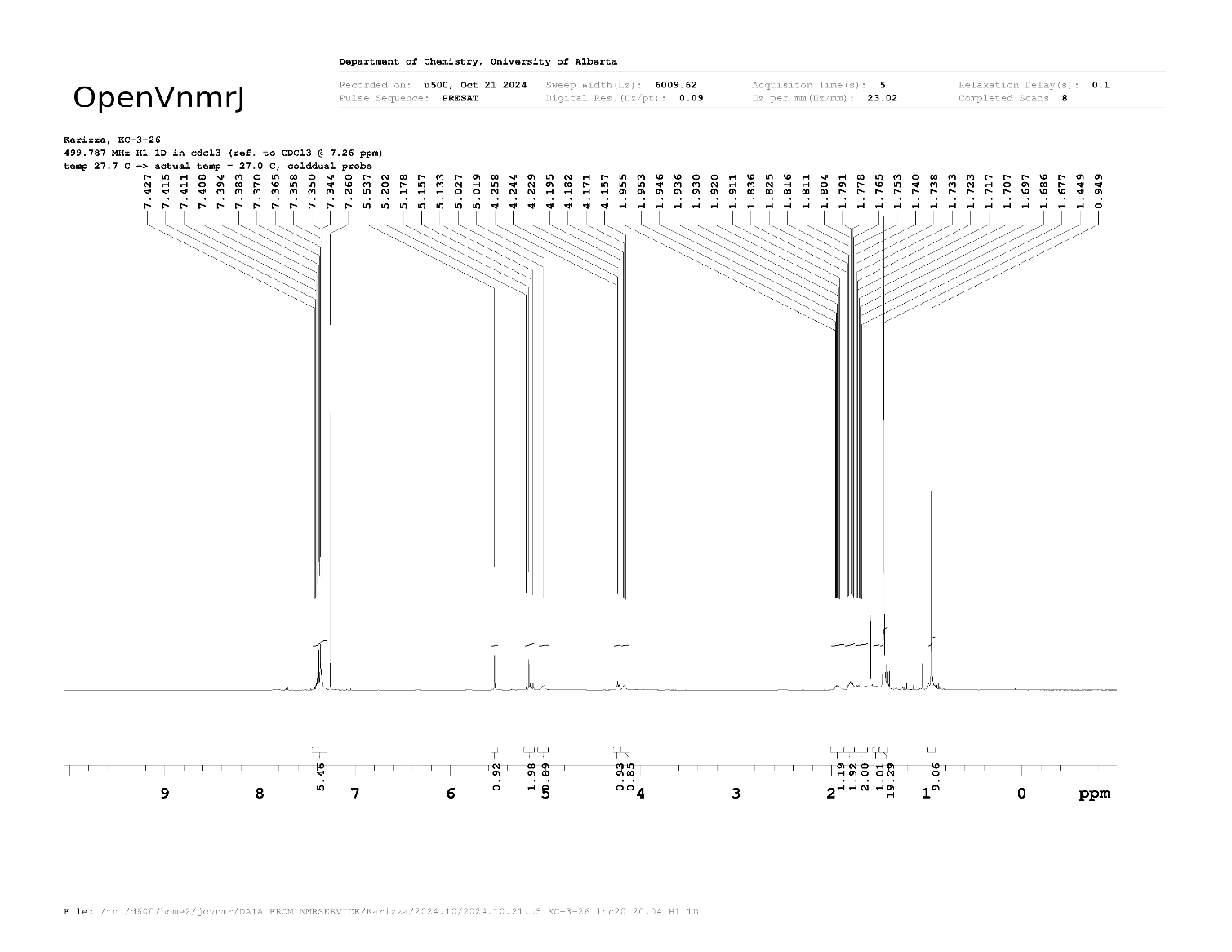


**13b – ^13^C NMR (125 MHz, CDCl_3_)**

**14a - ^1^H NMR (500 MHz, CDCl_3_)**

**14a – ^13^C NMR (125 MHz, CDCl_3_)**

**14b - ^1^H NMR (600 MHz, CDCl_3_)**

**14b – ^13^C NMR (125 MHz, CDCl_3_)**

**15b - ^1^H NMR (500 MHz, D_2_O)**

**15b - ^13^C NMR (125 MHz, D_2_O)**
